# The effect of weight loss before in vitro fertilization on reproductive outcomes in women with obesity : A systematic review and meta-analysis

**DOI:** 10.7326/ANNALS-24-01025

**Published:** 2025-08-12

**Authors:** Moscho Michalopoulou, Susan Ann Jebb, Alice Hobson, Shen Chuen Khaw, Richard Stevens, Pedro Melo, Stella Jane Pierce Haffner, Kathryn Sheridan Clay, Sarah Mounsey, Ingrid Granne, Lee Lim, Tim Child, Nerys Marie Astbury

**Affiliations:** 1 Nuffield Department of Primary Care Health Sciences, University of Oxford, Oxford, OX2 6GG, United Kingdom; 2 Oxford University Hospitals NHS Foundation Trust, John Radcliffe Hospital, Oxford, OX3 9DU, United Kingdom; 3 Nuffield Department of Women’s and Reproductive Health, University of Oxford, Women’s Centre John Radcliffe Hospital, Oxford,, OX3 9DU, United Kingdom

## Abstract

**Background:**

It is unclear if weight loss prior to IVF improves reproductive outcomes in women with obesity.

**Purpose:**

To assess whether weight loss interventions prior to IVF improve reproductive outcomes.

**Data Sources:**

Five electronic databases through 27 May 2025.

**Study selection:**

Randomized controlled trials (RCTs) in women with obesity offered a weight loss intervention prior to planned IVF.

**Data Extraction:**

Dual independent screening, data extraction, risk of bias (RoB) and certainty of evidence assessment. Primary outcomes were pregnancy and live birth rates. Where appropriate, studies were pooled using random-effects meta-analyses.

**Data Synthesis:**

Twelve RCTs (1,921 participants randomized) were included. Seven of 12 had high RoB. There was moderate certainty that pre-IVF weight loss interventions were associated with an increase in total pregnancy rates (RR 1.21, 95% CI 1.02 to 1.44; 11 studies) and in pregnancies resulting from unassisted conception (RR 1.47, 95% CI 1.26 to 1.73; 10 studies), whereas the effect on pregnancies resulting solely from IVF was uncertain. Weight loss interventions were not associated with pregnancy loss rates (RR 1.05, 95% CI 0.98 to 1.13; 8 studies; moderate certainty), but their effect on live birth rates was unclear (RR 1.15, 95% CI 0.95 to 1.40; 9 studies; very low certainty).

**Limitations:**

Small, high-RoB studies, often not reporting important outcomes such as live births. Substantial clinical and methodological heterogeneity was unexplained by exploratory analyses.

**Conclusions:**

Weight loss interventions before IVF appear to increase the chances of pregnancy, especially unassisted conceptions. However, studies were small, and heterogeneity made it difficult to determine the benefit of any particular intervention.

**Primary Funding Source:**

NIHR Applied Research Collaboration Oxford and Thames Valley (PROSPERO CRD42023441457).

## Introduction

Obesity is associated with increased risk for female infertility (1). Hormone dysregulation (e.g. leptin, ghrelin, resistin, adiponectin), insulin resistance, low levels of sex hormone-binding proteins, high androgen levels, chronic inflammation, and polycystic ovary syndrome (PCOS), are associated with obesity and can lead to ovulatory dysfunction (2,3). Obesity can also negatively affect female fertility even in the presence of regular ovulation (4). As a result, many women with obesity and infertility seek assisted reproductive technology (ART) treatment, including in vitro fertilization (IVF) in order to help them conceive (5), despite observational evidence that IVF outcomes are poorer in women with obesity (1, 6, 7). Obesity is associated with requirement for higher doses of, and reduced responsivity to ovarian stimulation medication, as well as reduced IVF success, increased chances of miscarriage if a pregnancy is achieved, and as a result, lower live birth rates (1, 6, 7). It is plausible that advising women to lose weight before undergoing IVF could improve IVF treatment outcomes. Weight loss improves hormone balance, increases insulin sensitivity, and reduces androgen levels and inflammation (8, 9), which can in turn lead to improved sensitivity and response to ovarian stimulation, improved oocyte quality and uterine function, and higher chances of conception (9). Pre-pregnancy weight loss may also increase the chances of a healthy pregnancy, as it can lower the risk of complications like gestational diabetes and preeclampsia (10, 11).

The National Institute for Health and Care Excellence in England states that women with a body mass index (BMI) ≥30 kg/m^2^ seeking IVF should be advised that losing weight will likely increase their chances of conception and that they may benefit from attending a group-based weight loss program (12). Other organizations have similar recommendations for weight loss before commencing IVF (13–16), but generally women are not offered support to lose weight (17). However, clinical guidelines suggesting weight loss prior to ART were based mainly on observational studies and only a few small randomized controlled trials (RCTs) showing poorer fertility treatment outcomes with increasing BMI, and improved ovulation patterns and higher conception rates following weight loss in women with obesity who are infertile and/or seeking fertility treatment (12–15). A systematic review a decade ago, explored the impact of weight loss interventions in women with overweight or obesity prior to undergoing ART, including IVF. Authors reported that weight loss interventions were associated with increased pregnancy and/or live birth rates in 8 of the 11 included studies, but only two RCTs were included (18). Since then, there have been several additional trials conducted in women with overweight or obesity who were candidates for IVF. A recent systematic review and meta-analysis of a limited group of 6 RCTs testing intensive weight loss programs in women seeking IVF, found no evidence of an effect on pregnancy or live birth rates (19).

We aimed to systematically review and synthesize the available evidence to address whether weight loss interventions of any kind, preceding IVF in women obesity, influence reproductive outcomes.

## Methods

### Search strategy and selection criteria

This systematic review was registered prospectively on PROSPERO (CRD42023441457). We searched five databases from 1980 to 27th May 2025: Medline (OvidSP), Embase (OvidSP), PsycINFO (OvidSP), CINAHL (EBSCOHost), and Cochrane Central Register of Controlled Trials. A subject specialist librarian developed the search strategy using key concepts of obesity, overweight, female infertility, weight loss interventions and pregnancy outcomes (Table S1). Here, we report findings from RCTs only. Papers reporting RCTs conducted in adult women (age ≥18 years) with BMI ≥27 kg/m^2^ (to capture obesity also in non-White ethnicities), seeking IVF with or without intracytoplasmic sperm injection treatment for infertility, comparing any type of weight loss intervention with a comparator which included no/minimal intervention (e.g. brief advice, leaflet) or alternative active weight loss program, and reporting pregnancy outcomes, were eligible for inclusion. No language limits were applied. We also screened reference lists of included studies and previous relevant systematic reviews.

After deduplication, two independent reviewers (NMA, KS, MM, AH, SJPH) screened titles and abstracts, and full-text articles in duplicate, using Covidence software (Veritas Health Innovation). Discrepancies were resolved by discussion. Where eligibility could not be determined based on the information provided in the full-text article, the article’s authors were contacted for clarification.

### Data extraction

One reviewer (AH, SCK, NMA, SM, SJPH) independently extracted key study information, participant characteristics, and outcomes of interest. Intervention characteristics were extracted using the Template for Intervention Description and Replication checklist (20). All extracted data were checked by a second reviewer (MM).

### Outcomes of interest

Outcome data were extracted on numbers of participants achieving pregnancy (defined as pregnancy confirmed by ultra-sonographic visualisation of one or more gestational sacs or definitive clinical signs of pregnancy) without IVF (unassisted pregnancy), with IVF (treatment-induced pregnancy), and in total (unassisted plus treatment-induced), and those delivering a live infant. We also extracted where available, weight change, number of fertility treatment cycles, time to pregnancy, delivery method, number of participants with biochemical pregnancy (pregnancy diagnosed only by detection of beta-human chorionic gonadotropin), with pregnancy loss (including biochemical pregnancy which did not progress to clinical pregnancy, miscarriage, ectopic pregnancy, and stillbirth), and with infants with congenital abnormality and neonatal death.

### Data synthesis

For event-related outcomes we calculated risk ratios (RR) and 95% confidence intervals (95% CI), with the number of participants randomized in each group as the denominator. We also calculated the mean difference in weight change between groups from baseline to follow-up and the accompanying standard error (SE), calculating these when necessary according to Cochrane guidelines (21).

### Statistical analysis

Included studies delivered heterogeneous weight loss interventions. However, our primary analysis aimed to assess the effect of weight loss, and not to evaluate the efficacy of any specific weight loss program on reproductive outcomes. All weight loss interventions act through the same mechanism; attaining and maintaining an energy deficit, which is achieved by reduction in energy intake and/or increase in energy expenditure. There is also no hypothesis why specific interventions may have differential impacts on IVF outcomes. We therefore quantitatively pooled event-related reproductive outcomes where we judged that the direction of the point estimates from individual studies was relatively consistent and 95% CIs adequately overlapped. Where meta-analysis was conducted, we used generic inverse variance random-effects models, applying the Hartung-Knapp-Sidik-Jonkman option to the DerSimonian-Laird simple method of moments estimator (22), and also present the pooled outcomes using fixed-effects meta-analysis with the Mantel-Haenszel, given that some of the meta-analyses included a small number of studies. Studies reporting zero events in both groups were omitted from forest plots and meta-analyses.

The effect of potential modifiers on reproductive outcomes was explored by grouping studies with similar intervention and comparator. We also sorted forest plots by mean difference in weight change between groups, mean age, mean baseline BMI, and proportion of participants with PCOS in the sample, to visually inspect any emerging patterns. Sensitivity analyses excluded studies at overall high risk of bias (RoB). We planned additional sensitivity analyses incorporating time by re-calculating RRs using the number of treatment cycles undertaken as the denominator for event-related outcomes. However, this was only feasible for treatment-induced pregnancy, because most studies did not report data separately for unassisted pregnancies (where there is no longer need for an IVF cycle) leading to live birth or pregnancy loss. Analyses by other important predictors of IVF outcomes, including cause and duration of infertility, and follow-up time, were not possible because of limited reporting.

The difference in weight change between similar intervention and comparator types was meta-analysed using generic inverse variance random-effects models, applying the HKSJ option. Forest plots of weight change were sorted by PCOS, and in a sensitivity analysis, studies with overall high RoB were excluded. In all meta-analyses, the I^2^ statistic was used to quantify relative heterogeneity (21).

We further explored whether the magnitude of weight change was associated with reproductive outcomes, through Sidik Jonkman random-effects meta-regression of the logRRs against the mean difference in weight change between groups, adjusting for comparator type.

Funnel plots and Egger’s test explored publication or small study bias for unassisted, treatment-induced, and total pregnancies, live births, and pregnancy loss. All analyses were conducted in Stata, version 17.0 (StataCorp). Meta-analyses used metan, and meta-regressions, funnel plots, and Egger’s tests used the meta command.

### Critical appraisal

Reviewers (AH, SCK, SM, SH and MM) independently assessed RoB of the RCTs in duplicate, using the Revised Cochrane RoB 2 tool (23), based on total pregnancy and live birth outcomes, in each of the following domains: randomization process, deviations from the intended interventions, missing outcome data, measurement of the outcome, and selection of the reported result. Overall ratings were taken from the most biased rating. Two reviewers (MM and NMA) assessed certainty of evidence, using the GRADE approach (24, 25). Disagreements were discussed until consensus was reached.

### Role of the funding source

Salary support to conduct this review was provided by the National Institute for Health Research Applied Research Collaboration Oxford and Thames Valley, who had no role in the study design, collection, analysis, and interpretation of data, in the writing of the report, and in the decision to submit the article for publication.

## Results

### Characteristics of included studies

We identified 5,748 potentially eligible records and 46 RCT articles were included reporting findings from 12 RCTs (1,921 randomly assigned participants) (26–38) (Figure A1). All studies were conducted in upper-middle or high-income economies. Important sources of heterogeneity are summarized in Table 1 and detailed in Table S3.

Four studies excluded women with higher BMI (>35 or 40) (26–28, 32), but only in one of these the mean baseline BMI was <30 (27). The remaining studies, either set a high limit for eligible BMI (1 study) (34, 35), or did not set a maximum limit (7 studies) (29–31, 33, 36–38). From the latter, mean baseline BMI was <30 in 3 studies (30, 37, 38). Participants were typically women in their early thirties (median [interquartile range – IQR] 31.6 [30.6 – 32.4] years; available from 11 studies), with median (IQR) baseline BMI 33.6 (29.2 – 36.2) kg/m^2^.

Seven studies reported a median (IQR) duration of infertility of at least 3.6 (3.2 – 4.7) years (26, 28, 32, 33, 36–38). Whilst 3 studies (27, 36, 38) excluded women where the male partner had infertility, in 5 studies (26, 28, 32, 33, 37), about 22.1% had a male partner with infertility.

Two studies excluded women with PCOS (26, 30), 2 studies included only women with PCOS (36, 38), and the remaining studies did not restrict eligibility by PCOS. Across 9 studies, a minimum of a quarter of participants (24.2%) had PCOS (26–28, 30, 32–36, 38).

Five studies used **diet modification** inducing a typical energy deficit of 500-800 kcal/day but maintaining total energy intake >1000 kcal/day (26–30). All but one of these (27) were accompanied by advice on physical activity. In one study, the main intervention was an **exercise** program, accompanied by advice to follow standard healthy eating recommendations (31). Three studies tested **low-energy diets**, which typically use liquid formula products to replace real food and induce a much larger energy deficit with total energy intake of ≤1000 kcal/day, followed by re-introduction of foods for weight stabilization before IVF (32–35). All but one (32) low-energy diets were accompanied with physical activity advice. In 3 studies, the main intervention was **pharmacotherapy** (orlistat, liraglutide, exenatide), accompanied by diet and physical activity advice (36–38). The median (range) duration of the active weight loss phase was 12 weeks (5–24).

Comparators comprised usual care, but in 6 studies they involved no/minimal intervention (26–30, 32), with participants in some studies accessing immediate IVF. In the remaining 6 studies (31, 33–38), usual care involved an alternative weight loss program of lower intensity.

Participants across all intervention groups lost 4 kg more than the comparator groups (mean difference in weight change intervention vs comparator: -4.10 kg, 95% CI -6.43 to -1.77; 11 studies; 1,769 participants) (Figure S1). Only 2 studies of low-energy diet had substantial weight loss difference between groups (32, 34, 35). Between-group difference in weight change was larger when interventions were compared with no/minimal intervention than compared with an active comparator (Figures S1-S3).

The average (range) follow-up for reproductive outcomes was 9.8 (1.3-18) months for intervention vs 11.5 (4.3-24) months for comparator groups.

Seven of 12 RCTs (58.3%) were judged to be at overall high RoB (Figure S4, Figure A2, and Table S4).

### Effect of weight loss interventions

#### Unassisted pregnancies

**Figure 1:**
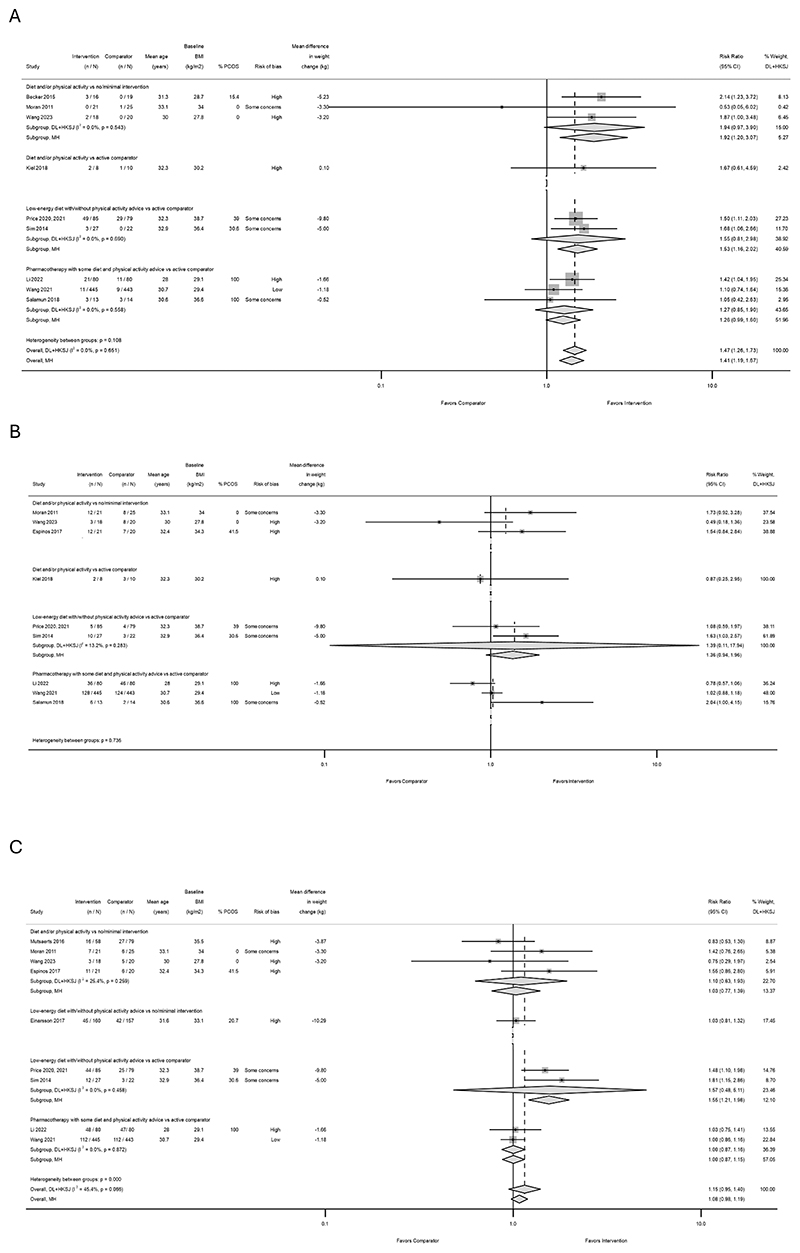
Effect of weight loss interventions on: (A) unassisted pregnancy, (B) treatment-induced pregnancy, and (C) live birth rates, grouped by intervention and comparator type, sorted by mean difference in weight change between groups n: number of events; N: number of participants randomized; CI: confidence interval; BMI: body mass index; PCOS: polycystic ovary syndrome; CI: confidence interval; DL: DerSimonian–Laird; HKSJ: Hartung–Knapp–Sidik–Jonkman; MH: Mantel-Haenszel. All but one (Becker 2015) diet interventions were accompanied by physical ativity advice; In Kiel 2018, the main intervention being tested was a physical activity program, but it was also accompanied by some dietary advice; All but one (Einarsson 2017) low-energy diet interventions were accompanied by some physical activity advice; All pharmacotherapy interventions were accompanied by both diet and physical activity advice. Mean age for women specifically seeking IVF was unknown from Mutsaerts 2016; % PCOS in the sample was unknown from Mutsaerts 2016, Wang 2021 and Kiel 2018, either because it was not reported, or it was not reported specifically for women seeking IVF; Espinos 2017 did not provide weight data at follow-up for the comparator, therefore it was not possible to calculate the mean difference in weight change between groups; Li 2022 reported events and weight data from per protocol analysis only; Price 2020, 2021 reported weight data from per protocol analysis only; We were able to obtain weight and total conception data for women specifically seeking IVF from authors of Mutsaerts 2016. A. Becker 2015 only reported unassisted pregnancy rates, therefore, total pregnancy rates are not known; C. Unassisted pregnancies seem to have been taken into account in live birth rates reported by Einarsson 2017, Sim 2014, Price 2020, 2021, Espinos 2017, Wang 2023, and Wang 2021; For Moran 2011 and Li 2022, it is suggestive that unassisted pregnancies were not taken into account in the reported live birth rates.

Ten studies (1,466 participants randomized) reported unassisted pregnancy rates (26–28, 30, 31, 33–38) (Figure 1A). The direction of the effect favored the intervention in 8 studies. However, most studies had a few unassisted pregnancies, resulting in wide CIs.

Overall, weight loss interventions prior to IVF were associated with greater unassisted pregnancy rates (RR 1.47, 95% CI 1.26 to 1.73; I^2^=0.0%). The effect size was greater in studies with comparators receiving no/minimal intervention (compared to an active weight loss comparator), although formal comparison was limited due to the small number of studies and events. However, both low-energy diet studies saw a larger amount of weight change and an improvement in number of events vs active comparator.

No consistent pattern was observed when studies were sorted by the magnitude of difference in weight change between groups (Figures 1A and S5), age (Figure S6), or baseline BMI (Figure S7), but we observed a tendency for fewer unassisted pregnancies with increasing proportion of women with PCOS in the sample (Figure S8). Findings were not meaningfully changed after excluding studies at high RoB (Figure S9).

#### Treatment-induced pregnancies

Nine studies (1,431 participants randomized) reported pregnancies following IVF treatment (26, 28, 30, 31, 33–38) (Figure 1B). The direction of the effect favored the weight loss intervention in 5 studies, favored the comparator in 3 studies, and was close to null in 1 study. CIs were wide throughout, with the exception of a single large study (Wang et al. 2021) (37), which saw almost no difference between pharmacotherapy intervention and active comparator (RR 1.02, 95% CI 0.88 to 1.18). The numerical heterogeneity within subgroups, as well as clinical and methodological heterogeneity, precluded pooling across all studies for this outcome.

There was no evidence of a difference in effects between intervention-comparator subgroups. No clear pattern was observed when studies were sorted by weight loss magnitude (Figures 1B and S10), age, BMI, or proportion of PCOS in the sample (Figures S11-S13).

After excluding studies at high RoB, 5 studies (1,174 participants randomized) remained (26, 33, 34–37) (Figure S14). The direction of the effect was more consistent and in favor of the intervention in all of these studies. CIs remained wide but overlapped to a greater extent than the primary analysis. As such, we meta-analysed these 5 studies. The overall summary estimate had wide CI and crossed the null (RR 1.33, 95% CI 0.91 to 1.95; I^2^=54.2%) (Figure S14). Sensitivity analysis using the number of treatment cycles in the denominator to calculate RRs, did not meaningfully change the results (Figure S15).

#### Live births

Nine studies (1,840 participants randomized) followed-up pregnancies until birth to report live birth outcomes (26, 28, 29, 30, 32–35, 37, 38), but only 6 of these included live births resulting from unassisted conceptions in the live birth outcomes (28, 29, 32–35, 37) (Figure 1C). The direction of the effect favored the weight loss intervention in 4 studies, favored the comparator in 2 studies, and was equal or close to null in 3 studies. The overall summary estimate crossed the null (RR 1.15, 95% CI 0.95 to 1.40; I^2^=45.4%).

There were more live births in the low-energy diet versus active comparator subgroup, compared with other subgroups, but only two studies were included in this subgroup. No pattern was observed when studies were sorted by weight loss difference between groups, age, BMI, or PCOS (Figures 1C and S16-S19). After excluding studies at high RoB, 4 studies remained (26, 33, 34, 35, 37), which precluded meaningful conclusions, despite the direction of point estimates being more consistent compared to the primary analysis (Figure S20).

#### Total pregnancies

Total pregnancy rates were available from 11 studies (1,885 participants randomized) (26, 28–38) (Figure A3). The direction of the effect favored the intervention in 8 studies, favored the comparator in 1 study, and was equal or close to null in 2 studies. Overall, weight loss interventions were associated with higher likelihood of total pregnancies (RR 1.21, 95% CI 1.02 to 1.44; I^2^=38.0%), with moderate heterogeneity.

Total pregnancy rates tended to be higher in the low-energy diet versus active comparator subgroup compared with other intervention-comparator subgroups, however, only two studies were included in this subgroup (33, 34, 35).

No pattern was observed when sorting by weight loss magnitude, age, BMI, and proportion of pre-existing PCOS (Figures A3 and S21-S24). After excluding studies at high RoB, the RR was greater than in the primary analysis, but I^2^ increased to a value which may represent substantial heterogeneity (RR 1.46, 95% CI 1.02 to 2.09; I^2^=69.1%; 5 studies; 1,173 participants randomized) (Figure S25).

#### Pregnancy loss

Eight studies (1,794 participants randomized) reported pregnancy loss data (28–30, 32–35, 37, 38) (Figure A4). There did not seem to be evidence of a difference in pregnancy loss rates between groups (RR 1.05, 95% CI 0.98 to 1.13; I^2^=0.0%). There were no differences between intervention-comparator subgroups. No consistent patterns were observed when sorting studies by weight loss magnitude, age, BMI, or PCOS (Figures A4 and S26-S29). Findings were not meaningfully changed in sensitivity analysis excluding studies at high RoB (Figure S30).

#### Other reproductive and neonatal outcomes

By in large, data were reported for one IVF treatment cycle, with only two studies specifying that they reported rates across multiple cycles (29, 33). Only a few studies reported data on other reproductive and neonatal outcomes and are narratively presented in the Supplement.

#### Effect of magnitude of weight loss

When forest plots of reproductive outcomes were sorted by the magnitude of the difference in weight change between groups above, we did not observe clear trends. There was also no evidence of a dose-response relationship in exploratory meta-regressions of the effect of the magnitude of weight loss. However, these analyses could not be meaningfully interpreted because fewer studies were analysed, most interventions, apart from low-energy diets, led to similarly modest weight loss, and in some cases observed trends were driven by extreme values (Figure S31).

### Publication/small study bias

We did not observe asymmetry in the funnel plots, suggesting no publication or small study bias (Figure S32).

### Certainty of evidence

We judged the certainty of evidence as moderate for total pregnancies, unassisted pregnancies, and pregnancy loss, and very low for treatment-induced pregnancies and live births. The most frequent reasons for downgrading were concerns about RoB and imprecision due to wide 95% CIs (Table 2).

## Discussion

### Principal findings

Here, we present the findings from a comprehensive systematic review across a diverse range of studies on the effectiveness of various types of weight loss interventions prior to IVF on reproductive outcomes. Our findings indicate that there is moderate certainty that for women with obesity, participating in a weight loss program prior to IVF increases the likelihood of becoming pregnant, especially through unassisted conception. There was inconclusive evidence on the effect of weight loss interventions on treatment-induced pregnancies. Overall, evidence on the association of weight loss interventions before IVF on live births was uncertain, although there was moderate certainty of no association with pregnancy loss. Unfortunately, fewer studies reported live birth outcomes, not all studies followed-up unassisted conceptions to determine live birth, and evidence on live births was further limited by heterogeneity in study design and clinical characteristics of recruited populations. Only a few studies contributed data to each intervention and comparator subgroup, making it difficult to determine the benefit of any particular weight loss intervention. It was not possible to determine if the magnitude of weight change influences reproductive outcomes, because most studies reported similarly modest weight losses. There were also no clear patterns when studies were sorted by other potential effect modifiers including age, BMI, or presence of PCOS, but there tended to be fewer unassisted pregnancies with increasing number of women with PCOS. The interpretation results was unchanged in sensitivity analyses.

### Strengths and limitations

Our analyses followed established Cochrane methods (21), and searches were comprehensive and not limited by language. Unlike a previous systematic review which was limited to intensive weight loss interventions (19), we included all types of weight loss interventions, recognising that despite variability, all weight loss approaches, and by extent their potential benefits on reproductive outcomes, act commonly through reduction in energy intake. This can help clinicians advising patients on the effectiveness of weight loss before IVF, regardless of method used.

However, the review is also limited by the primary data. In particular, fewer studies reported live birth or pregnancy loss data, and not all studies followed-up unassisted conceptions to term, therefore, analyses of these outcomes did not always include the same participants. The follow-up timeframe of reproductive outcomes was not well-reported, and, in some studies, it was inconsistent between intervention and comparator groups. Moreover, there was marked variability in eligibility and characteristics of participants which affect IVF success and could have influenced the effect of weight loss interventions on outcomes. For example, duration and type of infertility are among the most important predictors of IVF success, but only a small number of studies reported this data. Also, it would be reasonable to exclude participants for whom male factor infertility was also present, but this would require an individual patient meta-analysis, which was beyond the scope of this study.

### Comparison with other studies

The present findings are consistent with a systematic review conducted a decade ago (18), which concluded, mostly from observational data, that most weight loss interventions lead to increased pregnancy and/or live birth rates in women with overweight or obesity seeking IVF (18). However, our findings conflict with those of a more recent review by Jeong et al., which included 6 RCTs and found no evidence that ‘intensive’ weight loss interventions (defined as including at least two out of three components of exercise, pharmacotherapy, and diet) prior to IVF influenced reproductive outcomes (19). Our present review includes all types of weight loss interventions (and not just more intensive interventions), given that less intensive interventions are more common in routine healthcare settings. Unlike Jeong et al., (19) we did not exclude studies that involved only women with PCOS, which is the most common endocrine disorder in women of reproductive age and common cause of infertility that is strongly linked with obesity (39–43). Interestingly, we observed a tendency for fewer unassisted pregnancies with increasing proportion of participants with PCOS.

### Interpretation and implications for future research

Our findings suggest that weight loss interventions prior to IVF increase total pregnancies, mainly through an increase in unassisted pregnancy rates. The limited number of poor-quality studies limits our conclusions about the effect of weight loss interventions before IVF on treatment-induced pregnancies, live births, and pregnancy loss, as well as the effect of the magnitude of weight loss on outcomes, though there was consistent indication from a limited number of studies that there was no difference in pregnancy loss rates between intervention and comparator groups.

There is no clear evidence indicating which weight loss interventions work best and for whom, because of the small number of studies and substantial clinical and methodological heterogeneity. For example, potential effect modifiers such as participant age and baseline BMI, could not be fully explored because it is hard to tease out effects of aggregate characteristics. Weight loss, which was the main difference between intervention and comparator groups, is the factor we hypothesized as the mediator for any effects on reproductive outcomes, and there are documented mechanisms through which weight loss can improve fertility and IVF success (8, 9). However, our results did not support a dose-response relationship between weight loss and reproductive outcomes. This does not mean that such relationship does not exist, but rather, that it is unclear if such a relationship exists, because of the small number of studies and large number of potential effect modifiers, as well as the modest and similar weight loss achieved. Thus any trends were driven by the more extreme values. It is also possible that the association of weight loss with reproductive outcomes may not be linear, or that a threshold effect may be present, but this could not be explored in the current review given the limited dataset.

An individual participant data meta-analysis could help to investigate the role of participant characteristics on the effect of weight loss interventions on reproductive outcomes. Larger RCTs can explore interventions of greater weight loss (for example, by further testing low-energy diets). RCTs should recruit women representative of the population typically seeking IVF, including women with PCOS, but to investigate the effect of weight loss before IVF on female fertility and IVF outcomes, male factor infertility should be excluded. Future RCTs should compare weight loss interventions to usual care or minimal control to disentangle whether the effect is driven by weight loss itself. They should ensure standardized follow-up timeframe of reproductive outcomes between intervention and comparator groups and report all core outcomes of infertility research (44). Importantly, future analyses should assess whether weight loss interventions before IVF are cost-effective, and whether any potential benefit from weight loss interventions before IVF is offset by weight gain during pregnancy.

## Conclusion

In summary, pre-conception weight loss in women with obesity seeking IVF increases the chances of pregnancy, especially through unassisted conception, which may negate the need for treatment, and does not seem to increase the risk for pregnancy loss, though evidence of the effect on live births was unclear. Most studies were small and led to only modest weight loss, precluding conclusions of which interventions and what magnitude of weight loss are most beneficial.

## Supplementary Material

Appendix

Supplement

## Figures and Tables

**Table 1:** Summary of sources of heterogeneity in the included studies

	Population							Intervention	Comparator	Outcome			
Study & Country	N randomized	Main eligibility criteria	Age mean (SD) years	Baseline BMI mean (SD) kg/m^2^	Duration of infertility mean (SD) years	Cause of infertility (% of participants analyzed)	PCOS (% of participants analyzed)	Intervention content (key features)	Comparator content (main features)	Intervention follow-up time for reproductive outcomes (months)	Comparator follow-up time for reproductive outcomes (months)	Difference in weight change intervention vs comparator (kg)	Overall risk of bias
**Diet and/or physical activity vs no/minimal intervention**													
Moran 2011 (26) Australia	46	18-40 years of age; BMI ≥28 and <40 kg/m^2^; No PCOS; Referred for first IVF cycle; At least one previous ART cycle	33.1 (3.4)	34.0 (4.4)	4.7 (2.4)	Female: 26.3 Male: 44.7 Combined: 15.8 Unexplained/ Other: 5.3	0	Hypocaloric diet* + Replacement of one daily meal with liquid formula + Physical conditioning and walking program	One-off standard advice on diet and lifestyle factors influencing fertility, with no active follow-up	NR but similar between groups	NR but similar between groups	-3.30	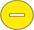
Becker 2015 (27) Brazil	35	>18 and <35 years of age; BMI >25 and ≤40 kg/m^2^; BMI >25 and <30 kg/m^2^ only included if waist circumference >80 cm; Any cause of female infertility	31.3 (3.0)	28.7 (2.8)	NR	Female: 84.6 Male: 0 Combined: 0 Unexplained/ Other: 15.4	≥15.4	Hypocaloric diet* + Supply of olive oil and dried fruit + Recommendation to not start any physical exercise throughout the study period	Usual diet + Recommendation to not start any physical exercise throughout the study period	4.5	4.5	-5.23	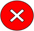
Espinos 2017 (28) Spain	41	18-37 years of age; BMI 30-40 kg/m^2^; Primary infertility; Presenting for first IVF cycle	32.4 (3.5)	34.3 (3.6)	5.0 (2.9)	Female: 75.9 Male: 63.4 Combined: NR Unexplained/ Other: 12.2	41.5	Hypocaloric diet* + Exercise program: walking and pedalling	Immediate IVF	NR but different between groups	NR but different between groups	NR	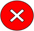
Mutsaerts 2016 (29) The Netherlands	137 (IVF only participants)	18-39 years of age; BMI ≥29 kg/m^2^; Chronic anovulation or oligomenorrhea or amenorrhea or an ovulatory cycle but unsuccessful in conceiving for at least 12 months	NR specifically for IVF participants	35.5 (4.9)	NR specifically for IVF participants	NR specifically for IVF participants	NR specifically for IVF participants	Hypocaloric diet* + Moderate-intensity physical activity + Motivational interviewing	Immediate IVF	18	24	-3.87	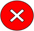
Wang 2023 (30) ^‡^ China	38 met eligibility criteria (groups A and D only)	18-36 years of age; BMI ≥25 kg/m^2^; Insulin resistance; No PCOS; Planning first or second IVF/ICSI	30.0 (3.2)	27.8 (2.4)	NR	NR	0	Hypocaloric diet* + Moderate-intensity physical activity	No intervention (presumably immediate IVF	NR but different between groups	NR but different between groups	-3.20	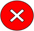
**Diet and/or physical activity vs active comparator**													
Kiel 2018 (31) Norway	18	>18 years of age	32.3 (5.0)	30.2 (2.4)	NR	NR	NR	High-intensity interval training + Encouragement to adhere to national diet recommendations	Regular advice about physical activity + Encouragement to adhere to national diet recommendations	1.8	4.3	0.10	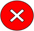
**Low-energy diet with/without physical activity advice vs no/minimal intervention**													
Einarsson 2017 (32) Sweden	317	18-38 years of age; BMI ≥30 and <35 kg/m^2^; Planning first, second, or third IVF	31.6 (4.2)	33.1 (1.4)	3.2 (1.9)	Female: 32.1 Male: 31.2 Combined: 29.8 Unexplained/ Other: 6.6	≥20.7	Low-energy liquid formula diet^†^ with re-introduction of solid foods and weight stabilization before IVF	Immediate IVF	1-1.5	4.5	-10.3	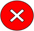
**Low-energy diet with/without physical activity advice vs active comparator**													
Sim 2014 (33) Australia	49	18-37 years of age; BMI ≥30 kg/m^2^; The fertility unit had an upper BMI limit of 40	32.9 (3.2)	36.4 (4.7)	3.6 (1.6)	Female: 91.8 Male: 49.0 Combined: NR Unexplained/ Other: 6.1	≥30.6	Low-energy liquid formula diet^†^ followed by refeeding protocol leading to mild hypocaloric diet before IVF + Moderate-intensity physical activity + Psychological support	GP-led weight loss advice or referral to public weight loss service if BMI ≥ 35 (suggestive that responsibility to seek weight loss advice was on individual participant)	15	15	-5.0	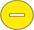
Price 2020, 2021 (34, 35) Australia	164	18-38 years of age; BMI 30-55 kg/m^2^; PCOS eligible regardless of ovulatory status; Excluded known irreversible infertility	32.3 (4.5)	38.7 (1.1)	NR	NR	39.0	Two low-energy liquid formulas^†^ per day plus a third low-calorie meal, followed by weight maintenance before IVF + Moderate-intensity physical activity	Hypocaloric diet* followed by weight maintenance before IVF + Moderate-intensity physical activity	13	13	-9.80	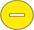
**Pharmacotherapy with some diet and physical activity advice vs active comparator**													
Salamun 2018 (36) Slovenia	27	≤38 years of age; BMI ≥30 kg/m^2^; PCOS; First or second IVF attempt	30.6 (4.2)	36.6 (4.2)	3.7 (2.4)	Female: 100 Male: NR but presumably 0 Combined: NR but presumably 0 Unexplained/ Other: NR but presumably 0	100	Liraglutide plus metformin + Hypocaloric diet* + Moderate-intensity physical activity	Metformin + Hypocaloric diet* + Moderate-intensity physical activity	15	15	-0.50	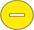
Wang 2021 (37) China	888	20-40 years of age; BMI ≥25 kg/m^2^; Exclude if history of 3 or more previous IVF/ICSI cycles	30.7 (4.0)	29.4 (3.1)	3.3 (2.2)	Female: 58.4 Male: 14.5 Combined: 20.5 Unexplained/ Other: 6.6	NR	Orlistat + Advice on lifestyle modifications aimed at a low-fat diet and high-quality physical activity	Placebo + Advice on lifestyle modifications aimed at a low-fat diet and high-quality physical activity	NR but similar between groups	NR but similar between groups	-1.20	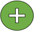
Li 2022 (38) China	160	20-40 years of age; BMI ≥24 kg/m^2^; PCOS; Inability to get pregnant more than 2 years without contraception; No male infertility	28.0 (3.7)	29.1 (4.0)	NR but ≥2.0	Female: 100 Male: NR but presumably 0 Combined: NR but presumably 0 Unexplained/ Other: 0	100	Exenatide and then switch to metformin + Lifestyle advice according to national guidance	Metformin + Lifestyle advice according to national guidance	NR but similar between groups	NR but similar between groups	-1.70	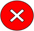

SD: standard deviation; BMI: body mass index; PCOS: polycystic ovary syndrome; ART: assisted reproductive technology; IVF: in vitro fertilization; ICSI: intracytoplasmic sperm injection; NR: not reported

* Hypocaloric diets induced an energy deficit of 500-800 kcal/day but maintained total energy intake >1000 kcal/day.

† Low-energy formula diets induced a large energy deficit with total energy intake of ≤1000 kcal/day.

‡ Wang 2023 included one more potentially eligible comparison: hypocaloric diet + physical activity + metformin vs metformin only, however, we chose to include only the most straightforward comparison (hypocaloric diet + physical activity vs no intervention) in this review. This was decided in order to avoid using the Wang 2023 as two separate studies into the meta-analyses, which could potentially affect confidence intervals due to data from the two separate entries not being completely independent

**Table 2:** GRADE assessment summary of findings table for the effect of weight loss interventions before IVF on reproductive outcomes

	Anticipated absolute effects* (95% CI)					
Outcomes	Risk with Comparator	Risk with Weight loss intervention	Relative effect (95% CI)	N randomized	Certainty of evidence (GRADE)	Comments
Unassisted pregnancies ^†^	7 per 100	**11 per 100** (9 to 13)	**RR 1.47** (1.26 to 1.73)	1466 (10 RCTs)	⨁⨁⨁◯ Moderate^¶ ||^	Weight loss interventions before IVF in women with obesity probably increase unassisted pregnancy rates compared with comparators.
Treatment-induced pregnancies ^†^	29 per 100	**33 per 100** (25 to 44)	**	1431 (9 RCTs)	⨁◯◯◯ Very low^§ †† ¶¶^	It is uncertain if weight loss interventions before IVF in women with obesity have an effect on treatment-induced pregnancy rates compared with comparators.
Total pregnancies^†‡^	37 per 100	**44 per 100** (37 to 53)	**RR 1.21** (1.02 to 1.44)	1885 (11 RCTs)	⨁⨁⨁◯ Moderate^§^	Weight loss interventions before IVF in women with obesity probably increase total pregnancy rates compared with comparators.
Live births	29 per 100	**32 per 100** (28 to 41)	**RR 1.15** (0.95 to 1.40)	1840 (9 RCTs)	⨁◯◯◯ Very low ^§ |||| ¶¶^	It is uncertain if weight loss interventions before IVF in women with obesity have an effect on live birth rates compared with comparators.
Pregnancy loss	13 per 100	**13 per 100** (13 to 14)	**RR 1.05** (0.98 to 1.13)	1794 (8 RCTs)	⨁⨁⨁◯ Moderate^§^	Weight loss interventions before IVF in women with obesity probably have no effect on pregnancy loss rates compared with comparators.

GRADE Working Group grades of evidence High certainty: we are very confident that the true effect lies close to that of the estimate of the effect. Moderate certainty: we are moderately confident in the effect estimate: the true effect is likely to be close to the estimate of the effect, but there is a possibility that it is substantially different. Low certainty: our confidence in the effect estimate is limited: the true effect may be substantially different from the estimate of the effect. Very low certainty: we have very little confidence in the effect estimate: the true effect is likely to be substantially different from the estimate of effect.

* The risk in the intervention group (and its 95% confidence interval) is based on the assumed risk in the comparison group and the relative effect of the intervention (and its 95% CI); RR: risk ratio; IVF: in vitro fertilization; RCT: randomized controlled trial;

† Pregnancy diagnosed by ultra-sonographic visualization of one or more gestational sacs or definitive clinical signs of pregnancy.

‡ Includes both unassisted and treatment-induced conceptions.

¶ Most studies were judged to have some concerns with regards to risk of bias (RoB). The main reason for concern was lack of clarity in the randomization process. It was judged however that these biases were unlikely to lower confidence in the effect estimate.

|| The confidence intervals of individual studies are wide, the total number of events is relatively small, and the confidence interval of the pooled effect includes appreciable benefit (i.e. includes RR >1.25).

§ More than half of the studies were judged to be at overall high risk of bias (RoB). The most frequent reason for high RoB was bias in the measurement of the outcome domain (RoB 4.2) because the follow-up timeframe differed between intervention and comparator groups. Within the studies with high RoB, there was critical limitation for 1 criteria, or some concerns for multiple criteria.

** The numerical, as well as clinical and methodological heterogeneity, precluded pooling across all studies for this outcome.

†† The point estimates varied across studies, the confidence intervals even within intervention-comparator subgroups had small overlap, and heterogeneity was largely unexplained by exploratory analyses.

‡‡ The point estimates varied across studies, the confidence intervals had enough overlap to qualify for pooling across all studies, but there was still some inconsistency, and heterogeneity was largely unexplained by exploratory analyses.

¶¶ Despite that the optimal information size threshold is met, the confidence intervals of individual studies are relatively wide, and/or the confidence interval of the pooled effect includes both no effect and appreciable benefit (i.e. includes RR>1.25).

|||| The point estimates varied across studies and the CIs had enough overlap to qualify for pooling across all studies, but there was still some inconsistency and heterogeneity was largely unexplained by exploratory analyses
