# Supplementary material for: The effect of weight loss before in vitro fertilization on reproductive outcomes in women with obesity : A systematic review and meta-analysis
Source: Ann Intern Med. Author manuscript; Available in PMC 2025 Aug 25. (PMC7618032; doi:10.7326/ANNALS-24-01025)
Supplement: Appendix [file EMS207928-supplement-Appendix.docx]

**Appendix**

[**Figure A1. Study selection** 3](#_Toc199878356)

[**Figure A2: Risk of bias assessment of individual included randomized controlled trials** 4](#_Toc199878359)

[**Figure A3. Total pregnancy rates, intervention vs comparator groups, grouped by intervention and comparator type, and sorted by mean difference in weight change between groups** 5](#_Toc199878360)

[**Figure A4. Pregnancy loss rates, intervention vs comparator groups, grouped by intervention and comparator type, and sorted by mean difference in weight change between groups** 6](#_Toc199878361)

## **Figure A1. Study selection**

**1** potentially eligible record

identified through manual search

**5747** potentially eligible records identified through database search

**3188** duplicates removed

**2560** records screened against title and abstract

**2356** records excluded

**204** full-text records

assessed for eligibility

**131** full-text records excluded

**6** BMI <27 kg/m^2^ or did not report data specifically for those with obesity

**61** not seeking IVF, or did not report data specifically for those seeking IVF

**12** no weight loss intervention or unclear if weight loss was a focus because only intervention was metformin (not an approved weight loss drug, though sometimes leads to weight loss)

**7** did not report any of our primary outcomes, or included only women with a pregnancy and/or live birth (i.e. pregnancy and/or live birth was not an outcome)

**10** wrong study design (e.g. review, commentary, crossover)

**6** non-RCTs did not report weight outcomes at all, or specifically for those with obesity, or those seeking IVF

**25** no results published though not ongoing studies, or published only as a conference abstract

**3** ongoing studies

**1** no abstract or full text could be found, only title

**73** eligible records including both RCTs and non-RCTs (but this publication is only focused on RCTs)

**46** records of RCTs

reporting on **12** RCTs

IVF: in vitro fertilization; BMI: body mass index; RCT: randomized controlled trial

## **Figure A2: Risk of bias assessment of individual included randomized controlled trials**


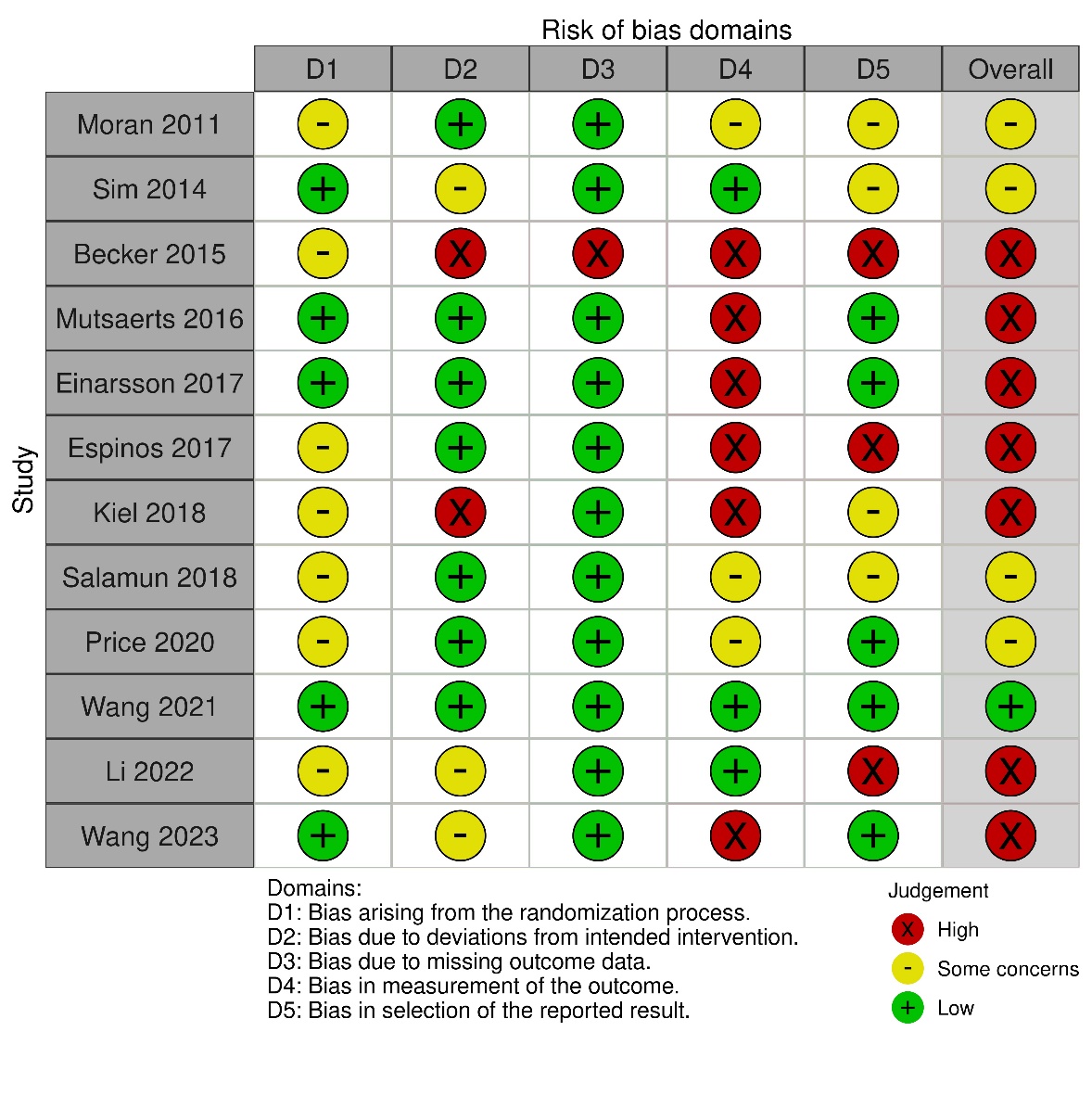


## **Figure A3. Total pregnancy rates, intervention vs comparator groups, grouped by intervention and comparator type, and sorted by mean difference in weight change between groups**


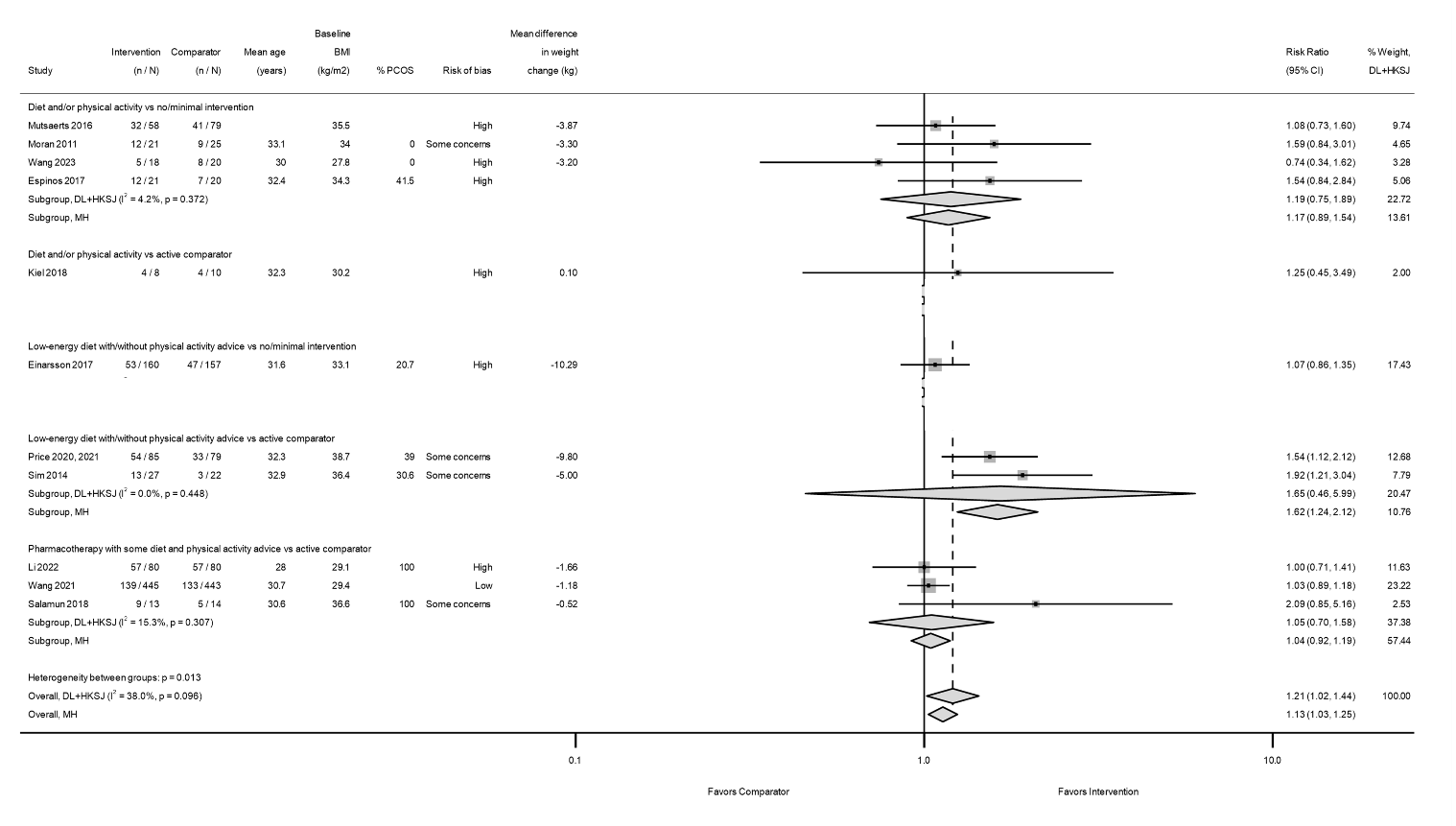


n: number of events; N: number of participants randomized; CI: confidence interval; BMI: body mass index; PCOS: polycystic ovary syndrome; CI: confidence interval; DL: DerSimonian–Laird; HKSJ: Hartung–Knapp–Sidik–Jonkman; MH: Mantel-Haenszel

Mean age for women specifically seeking IVF was unknown from Mutsaerts 2016; % PCOS in the sample was unknown from Mutsaerts 2016, Wang 2021 and Kiel 2018, either because it was not reported, or it was not reported specifically for women seeking IVF; Espinos 2017 did not provide weight data at follow-up for the comparator, therefore it was not possible to calculate the mean difference in weight change between groups; Becker 2015 only reported unassisted pregnancy rates, therefore, total pregnancy rates are not known; Li 2022 reported weight and event data from per protocol analysis only; Price 2020, 2021 reported weight data from per protocol analysis only; We were able to obtain weight and total pregnancy data for women specifically seeking IVF from authors of Mutsaerts 2016.

## **Figure A4. Pregnancy loss rates, intervention vs comparator groups, grouped by intervention and comparator type, and sorted by mean difference in weight change between groups**


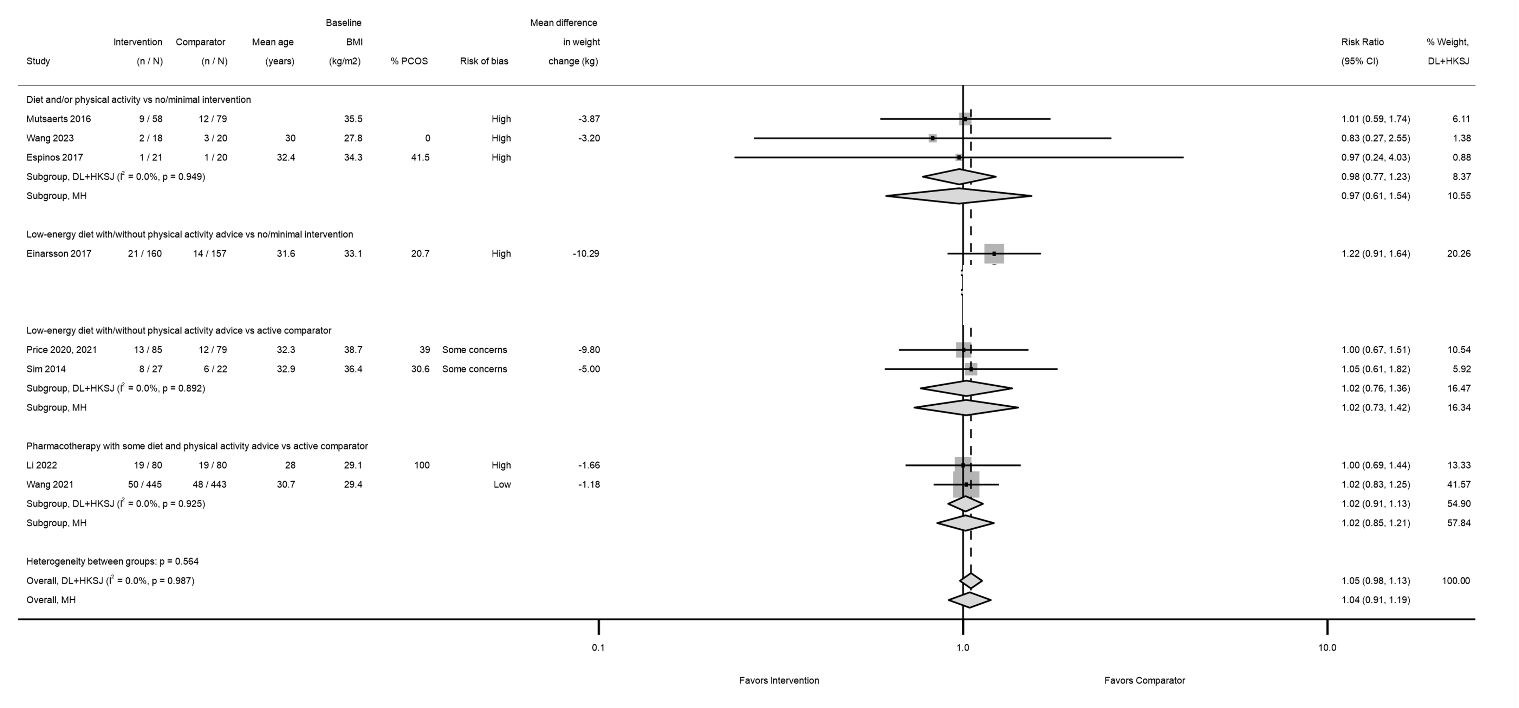


n: number of events; N: number of participants randomized; CI: confidence interval; BMI: body mass index; PCOS: polycystic ovary syndrome; CI: confidence interval; DL: DerSimonian–Laird; HKSJ: Hartung–Knapp–Sidik–Jonkman; MH: Mantel-Haenszel

Mean age for women specifically seeking IVF was unknown from Mutsaerts 2016; % PCOS in the sample was unknown from Mutsaerts 2016 and Wang 2021, either because it was not reported, or it was not reported specifically for women seeking IVF; Espinos 2017 did not provide weight data at follow-up for the comparator, therefore it was not possible to calculate the mean difference in weight change between groups; Li 2022 reported weight and event data from per protocol analysis only; Price 2020, 2021 reported weight data from per protocol analysis only; We were able to obtain weight and pregnancy loss data for women specifically seeking IVF from authors of Mutsaerts 2016. Unassisted pregnancies seem to have been taken into account in pregnancy loss rates reported by Einarsson 2017, Price 2020, 2021, Sim 2014, Espinos 2017, Wang 2023, and Wang 2021; For Li 2022, it is suggestive that unassisted pregnancies were not taken into account in the reported pregnancy loss rates.
