# Supplementary material for: The effect of weight loss before in vitro fertilization on reproductive outcomes in women with obesity : A systematic review and meta-analysis
Source: Ann Intern Med. Author manuscript; Available in PMC 2025 Aug 25. (PMC7618032; doi:10.7326/ANNALS-24-01025)
Supplement: Supplement [file EMS207928-supplement-Supplement.pdf]

Supplement

Table S1. Search strategies 3

Table S2: List of records excluded at full-text screening stage 12

Table S3. Detailed characteristics of included randomized controlled trials 20

Table S4. Detailed risk of bias assessment of included individual randomized controlled trials .. 28

Weight loss 51

Figure S1. Mean difference in weight change between intervention and comparator groups, grouped by intervention and comparator type 52

Figure S2. Mean difference in weight change between intervention and comparator groups, sorted by % PCOS in the sample (A: all studies together; B: grouped by intervention and comparator type) 53

Figure S3. Mean difference in weight change between intervention and comparator groups, grouped by intervention and comparator type, excluding studies at overall high risk of bias 54

Figure S4: Risk of bias of included randomized controlled trials 55

Figure S5. Unassisted pregnancy rates, intervention vs comparator groups, sorted by mean difference in weight change between groups (all studies together) 56

Figure S6. Unassisted pregnancy rates, intervention vs comparator groups, sorted by mean age (A: all studies together; B: grouped by intervention and comparator type) 57

Figure S7. Unassisted pregnancy rates, intervention vs comparator groups, sorted by baseline BMI (A: all studies together; B: grouped by intervention and comparator type) 58

Figure S8. Unassisted pregnancy rates, intervention vs comparator groups, sorted by % PCOS in the sample (A: all studies together; B: grouped by intervention and comparator type) 59

Figure S9. Unassisted pregnancy rates, intervention vs comparator groups, sorted by mean difference in weight change between groups, excluding studies at overall high risk of bias 60

Figure S10. Treatment-induced pregnancy rates, intervention vs comparator groups, sorted by mean difference in weight change between groups (all studies together) 61

Figure S11. Treatment-induced pregnancy rates, intervention vs comparator groups, sorted by mean age (A: all studies together; B: grouped by intervention and comparator type) 62

Figure S12. Treatment-induced pregnancy rates, intervention vs comparator groups, sorted by baseline BMI (A: all studies together; B: grouped by intervention and comparator type) 63

Figure S13. Treatment-induced pregnancy rates, intervention vs comparator groups, sorted by % PCOS in the sample (A: all studies together; B: grouped by intervention and comparator type) 64

Figure S14. Treatment-induced pregnancy rates, intervention vs comparator groups, grouped by intervention and comparator type, sorted by mean difference in weight change between groups, excluding studies at overall high risk of bias 65

Figure S15. Treatment-induced pregnancy rates, intervention vs comparator groups, grouped by intervention and comparator type, using the number of treatment cycles undertaken in the denominator, and sorted by mean difference in weight change between groups 66

Figure S16. Live birth rates, intervention vs comparator groups, sorted by mean difference in weight change between groups (all studies together) ... 67

Figure S17. Live birth rates, intervention vs comparator groups, sorted by mean age (A: all studies together; B: grouped by intervention and comparator type) ... 68

Figure S18. Live birth rates, intervention vs comparator groups, sorted by baseline BMI (A: all studies together; B: grouped by intervention and comparator type) ... 69

Figure S19. Live birth rates, intervention vs comparator groups, sorted by % PCOS in the sample (A: all studies together; B: grouped by intervention and comparator type) ... 70

Figure S20. Live birth rates, intervention vs comparator groups, grouped by intervention and comparator type, sorted by mean difference in weight change between groups, excluding studies at overall high risk of bias ... 71

Figure S21. Total pregnancy rates, intervention vs comparator groups, sorted by mean difference in weight change between groups (all studies together) ... 72

Figure S22. Total pregnancy rates, intervention vs comparator groups, sorted by mean age (A: all studies together; B: grouped by intervention and comparator type) ... 73

Figure S23. Total pregnancy rates, intervention vs comparator groups, sorted by baseline BMI (A: all studies together; B: grouped by intervention and comparator type) ... 74

Figure S24. Total pregnancy rates, intervention vs comparator groups, sorted by % PCOS in the sample (A: all studies together; B: grouped by intervention and comparator type) ... 75

Figure S25. Total pregnancy rates, intervention vs comparator groups, grouped by intervention and comparator type, sorted by mean difference in weight change between groups, excluding studies at overall high risk of bias ... 76

Figure S26. Pregnancy loss rates, intervention vs comparator groups, sorted by mean difference in weight change between groups (all studies together) ... 77

Figure S27. Pregnancy loss rates, intervention vs comparator groups, sorted by mean age (A: all studies together; B: grouped by intervention and comparator type) ... 78

Figure S28. Pregnancy loss rates, intervention vs comparator groups, sorted by baseline BMI (A: all studies together; B: grouped by intervention and comparator type) ... 79

Figure S29. Pregnancy loss rates, intervention vs comparator groups, sorted by % PCOS in the sample (A: all studies together; B: grouped by intervention and comparator type) ... 80

Figure S30. Pregnancy loss rates, intervention vs comparator groups, grouped by intervention and comparator type, sorted by mean difference in weight change between groups, excluding studies at overall high RoB ... 81

Other reproductive outcomes ... 82

Figure S31. Meta-regressions of weight change between intervention and comparator groups, and reproductive outcomes, by comparator type ... 83

Figure S32. Funnel plots of included randomized controlled trials for reproductive outcomes ... 86

Table S1. Search strategies

Medline (Ovid MEDLINE® Epub Ahead of Print, In-Process & Other Non-Indexed Citations, Ovid MEDLINE® Daily and Ovid MEDLINE®)		
	**Searches**	**Results**
1	reproductive techniques, assisted/ or exp fertilization in vitro/	53367
2	Infertility, Female/	32461
3	(Intracytoplasmic sperm injection? or Intra-cytoplasmic sperm injection? or icsi or in vitro fertili* or invitro fertili* or ivf or (assisti* reproduct* adj (therap* or treatment or technolog*))).ti,ab,kf.	52718
4	Preconception Care/	2889
5	((pre-pregnan* or prepregnan* or pre-concept* or preconcept*) and (care or healthcare or service? or therap* or treatment or surg* or program* or intervention)).ti,kf. or ((pre-pregnan* or prepregnan* or pre-concept* or preconcept*) adj5 (care or healthcare or service? or therap* or treatment or surg* or program* or intervention)).ab.	3179
6	1 or 2 or 3 or 4 or 5	101620
7	overweight/ or obesity/ or obesity, abdominal/ or obesity, maternal/ or obesity, morbid/	280501
8	(obes* or overweight).ti,ab,kf.	457664
9	Polycystic Ovary Syndrome/	20001
10	(polycystic ovar* syndrome or poly-cystic ovar* syndrome or pcos).ti,kf.	18538
11	7 or 8 or 9 or 10	525124
12	body weight changes/ or weight loss/	47444
13	((bodyweight or weight) adj3 (loss or losing or lose or lost or reduc* or manage* or control)).ti,ab,kf.	189760
14	diet therapy/ or caloric restriction/ or exp diet, carbohydrate-restricted/ or diet, fat-restricted/ or exp diet, high-protein/ or diet, reducing/ or dietary approaches to stop hypertension/	38657
15	Exercise Therapy/ or exp Exercise/	310733
16	Obesity Management/	339
17	Obesity/dh, th	27446
18	Weight reduction programs/	3096
19	((low* fat or reduc* fat? or low* calorie* or reduc* calorie* or low* energy or reduc* energy or hypocalor* or calori* restrict* or calorie control* or low* glyc* or reduc* glyc* or low* carbohydrate? or reduc* carbohydrate? or high* protein? or increas* protein? or keto* or paleo* or intermittent fasting or meal replacement? or replacement meal?) adj2 (diet? or plan* or regim* or program* or intervention?)).ti,ab,kf.	25564
20	((weight loss or weight control or weight management or weight reduction) adj2 (diet? or plan* or regim* or program* or intervention? or group? or class*)).ti,ab,kf.	14845
21	((exercise or physical activity) adj2 (plan* or regim* or program* or intervention? or train* or group? or class*)).ti,ab,kf.	77941
22	((lifestyle or life style) adj2 (plan* or regim* or program* or intervention? or train* or group? or class*)).ti,ab,kf.	17240
23	exp Anti-Obesity Agents/	21730
24	Obesity/dt	14304
25	((antiobesity or anti-obesity) adj3 (medication? or medicine? or drug? or agent?)).ti,kf.	970
26	(liraglutide or saxenda or victoza or semaglutide or ozempic or wegovy or tirzepatide or exenatide or bydureon or byetta or lixisenatide or albiglutide or tanzeum or eperzan or dulaglutide or trulicity or phentermine or adipex-p or suprenza or topiramate or topamax or qsymia or lorcaserin or belviq or (naltrexone and bupropion) or sibutramine or rimonabant or benzphetamine or diethylpropion or tenuate or phendimetrazine or bontril or orlistat or xenical).ti,ab,kf.	21825
27	Obesity/su	8349
28	bariatrics/ or exp bariatric surgery/	37314
29	(bariatric surg* or obesity surg* or metabolic surg* or weight loss surg* or gastric sleeve or intragastric sleeve or sleeve gastr* or ileal sleeve or gastric band* or intragastric band* or gastric bypass* or intragastric bypass* or jejuno* bypass* or ileal bypass* or gastric balloon* or intragastric balloon* or roux-en-y or biliopancreatic diversion or bilio-pancreatic diversion or duodenal bypass*).ti,ab,kf.	49416
30	or/12-29	651491
31	6 and 11 and 30	775
32	pregnancy/ or exp pregnancy outcome/	1042746
33	pregnancy rate/ or birth rate/	24755
34	Time-to-Pregnancy/	353

35	(pregnancy or pregnancies or pregnant).ti,kf. or (pregnancy adj2 (rate? or clinical or outcome? or ongoing or successful or "time to")).ab.	370660
36	(live birth? or birth rate).ti,ab,kf.	42133
37	(stillbirth? or still birth? or ((foetus or fetus or foetal or fetal or pregnan*) adj (death? or mortality or loss)) or miscarriage? or spontaneous abortion?).ti,ab,kf.	67056
38	32 or 33 or 34 or 35 or 36 or 37	1126659
39	6 and 11 and 30 and 38	598
40	(baboon$1 or bovine$1 or canine$1 or cat$1 or chimpanzee$1 or cow$1 or dog$1 or feline$1 or goat$1 or hens or macque$1 or mice or monkey$1 or (mouse adj2 model$1) or murine$1 or ovine or pig$1 or porcine or (non-human adj2 primate$1) or sheep or rabbit$1 or rat or rats or rattus or rhesus or rodent$1 or zebrafish).ti.	2256480
41	39 not 40	585
42	limit 41 to yr="1980 -Current"	581
43	(2024* or 2025*).ed,ez,yr.	2437416
44	42 and 43	67
**Embase**		
1	in vitro fertilization/ or intracytoplasmic sperm injection/ or infertility therapy/	109556
2	exp female infertility/	58677
3	(Intracytoplasmic sperm injection? or Intra-cytoplasmic sperm injection? or icsi or in vitro fertili* or invitro fertili* or ivf or (assisti* reproduct* adj (therap* or treatment or technolog*))).ti,ab,kf.	89366
4	prepregnancy care/	3635
5	((pre-pregnan* or prepregnan* or pre-concept* or preconcept*) and (care or healthcare or service? or therap* or treatment or surg* or program* or intervention)).ti,kf. or ((pre-pregnan* or prepregnan* or pre-concept* or preconcept*) adj5 (care or healthcare or service? or therap* or treatment or surg* or program* or intervention)).ab.	4592
6	1 or 2 or 3 or 4 or 5	179504
7	*obesity/ or *abdominal obesity/ or *morbid obesity/	251839
8	(obes* or overweight).ti,ab,kf.	689233
9	*ovary polycystic disease/	26265
10	(polycystic ovar* syndrome or poly-cystic ovar* syndrome or pcos).ti,kf.	27427
11	7 or 8 or 9 or 10	735203
12	*body weight change/ or *body weight loss/	38176
13	((bodyweight or weight) adj3 (loss or losing or lose or lost or reduc* or manage* or control)).ti,ab,kf.	298724
14	*diet therapy/ or exp *dash diet/ or exp *diet restriction/ or exp *low calorie diet/ or *low fat diet/ or *modified atkins diet/ or exp *protein diet/ or exp *ketogenic diet/	63976
15	exp *exercise/ or exp *physical therapy/	238645
16	*Obesity Management/ or *Lifestyle Modification/	8411
17	obesity/dm, th	23550
18	*weight loss program/	1658
19	((low* fat or reduc* fat? or low* calorie* or reduc* calorie* or low* energy or reduc* energy or hypocalor* or calori* restrict* or calorie control* or low* glyc* or reduc* glyc* or low* carbohydrate? or reduc* carbohydrate? or high* protein? or increas* protein? or keto* or paleo* or intermittent fasting or meal replacement? or replacement meal?) adj2 (diet? or plan* or regim* or program* or intervention?)).ti,ab,kf.	36488
20	((weight loss or weight control or weight management or weight reduction) adj2 (diet? or plan* or regim* or program* or intervention? or group? or class*)).ti,ab,kf.	22662
21	((exercise or physical activity) adj2 (plan* or regim* or program* or intervention? or train* or group? or class*)).ti,ab,kf.	110284
22	((lifestyle or life style) adj2 (plan* or regim* or program* or intervention? or train* or group? or class*)).ti,ab,kf.	25542
23	exp *antiobesity agent/	19847
24	obesity/dt	19587
25	((antiobesity or anti-obesity) adj3 (medication? or medicine? or drug? or agent?)).ti,kf.	1402
26	(liraglutide or saxenda or victoza or semaglutide or ozempic or wegovy or tirzepatide or exenatide or bydureon or byetta or lixisenatide or albiglutide or tanzeum or eperzan or dulaglutide or trulicity or phentermine or adipex-p or suprenza or topiramate or topamax or qsymia or lorcaserin or belviq or (naltrexone and bupropion) or sibutramine or rimonabant or	37413

	benzphetamine or diethylpropion or tenuate or phendimetrazine or bontril or orlistat or xenical).ti,ab,kf.	
27	Obesity/su	14261
28	exp *bariatric surgery/	56599
29	(bariatric surg* or obesity surg* or metabolic surg* or weight loss surg* or gastric sleeve or intragastric sleeve or sleeve gastr* or ileal sleeve or gastric band* or intragastric band* or gastric bypass* or intragastric bypass* or jejuno* bypass* or ileal bypass* or gastric balloon* or intragastric balloon* or roux-en-y or biliopancreatic diversion or bilio-pancreatic diversion or duodenal bypass*).ti,ab,kf.	87410
30	or/12-29	775684
31	pregnancy/ or birth rate/ or fetus mortality/ or fetus outcome/ or live birth/ or pregnancy outcome/ or pregnancy rate/ or prenatal mortality/ or time to pregnancy/ or exp spontaneous abortion/ or exp fetus death/	897652
32	(pregnancy or pregnancies or pregnant).ti,kf. or (pregnancy adj2 (rate? or clinical or outcome? or ongoing or successful or "time to")).ab.	444870
33	(live birth? or birth rate).ti,ab,kf.	60741
34	(stillbirth? or still birth? or ((foetus or fetus or foetal or fetal or pregnan*) adj (death? or mortality or loss)) or miscarriage? or spontaneous abortion?).ti,ab,kf.	98657
35	31 or 32 or 33 or 34	1021331
36	6 and 11 and 30 and 35	950
37	(baboon$1 or bovine$1 or canine$1 or cat$1 or chimpanzee$1 or cow$1 or dog$1 or feline$1 or goat$1 or hens or macque$1 or mice or monkey$1 or (mouse adj2 model$1) or murine$1 or ovine or pig$1 or porcine or (non-human adj2 primate$1) or sheep or rabbit$1 or rat or rats or rattus or rhesus or rodent$1 or zebrafish).ti.	2430889
38	36 not 37	939
39	limit 38 to yr="1980 -Current"	938
40	(2024* or 2025*).dc,dd,yr.	3262532
41	39 and 40	153
**PsycINFO**		
1	reproductive technology/	2386
2	Human Females/ and Infertility/	490
3	(Intracytoplasmic sperm injection? or Intra-cytoplasmic sperm injection? or icsi or in vitro fertili* or invitro fertili* or ivf or (assisti* reproduct* adj (therap* or treatment or technolog*))).tw,id.	1224
4	((pre-pregnan* or prepregnan* or pre-concept* or preconcept*) and (care or healthcare or service? or therap* or treatment or surg* or program* or intervention)).ti,id. or ((pre-pregnan* or prepregnan* or pre-concept* or preconcept*) adj5 (care or healthcare or service? or therap* or treatment or surg* or program* or intervention)).ab.	542
5	1 or 2 or 3 or 4	3735
6	obesity/	30859
7	(obes* or overweight).tw,id.	55949
8	(polycystic ovar* syndrome or poly-cystic ovar* syndrome or pcos).mp.	669
9	6 or 7 or 8	56871
10	weight loss/	5060
11	((bodyweight or weight) adj3 (loss or losing or lose or lost or reduc* or manage* or control)).tw,id.	24052
12	diets/ or dietary treatment/	16956
13	exercise therapy/ or exp exercise/ or exp physical activity/	59665
14	weight control/	5722
15	lifestyle changes/	1694
16	((low* fat or reduc* fat? or low* calorie* or reduc* calorie* or low* energy or reduc* energy or hypocalor* or calori* restrict* or calorie control* or low* glyc* or reduc* glyc* or low* carbohydrate? or reduc* carbohydrate? or high* protein? or increas* protein? or keto* or paleo* or intermittent fasting or meal replacement? or replacement meal?) adj2 (diet? or plan* or regim* or program* or intervention?)).tw,id.	1932
17	((weight loss or weight control or weight management or weight reduction) adj2 (diet? or plan* or regim* or program* or intervention? or group? or class*)).tw,id.	4356
18	((exercise or physical activity) adj2 (plan* or regim* or program* or intervention? or train* or group? or class*)).tw,id.	15813
19	((lifestyle or life style) adj2 (plan* or regim* or program* or intervention? or train* or group? or class*)).tw,id.	3615
20	Obesity/ and Drug Therapy/	843

21	((antiobesity or anti-obesity) adj3 (medication? or medicine? or drug? or agent?)).ti,id.	43
22	(liraglutide or saxenda or victoza or semaglutide or ozempic or wegovy or tirzepatide or exenatide or bydureon or byetta or lixisenatide or albiglutide or tanzeum or eperzan or dulaglutide or trulicity or phentermine or adipex-p or suprenza or topiramate or topamax or qsymia or lorcaserin or belviq or (naltrexone and bupropion) or sibutramine or rimonabant or benzphetamine or diethylpropion or tenuate or phendimetrazine or bontril or orlistat or xenical).tw,id.	3170
23	bariatrics/ or exp bariatric surgery/	1537
24	(bariatric surg* or obesity surg* or metabolic surg* or weight loss surg* or gastric sleeve or intragastric sleeve or sleeve gastr* or ileal sleeve or gastric band* or intragastric band* or gastric bypass* or intragastric bypass* or jejuno* bypass* or ileal bypass* or gastric balloon* or intragastric balloon* or roux-en-y or biliopancreatic diversion or bilio-pancreatic diversion or duodenal bypass*).tw,id.	2094
25	or/10-24	105202
26	5 and 9 and 25	27
27	(baboon$1 or bovine$1 or canine$1 or cat$1 or chimpanzee$1 or cow$1 or dog$1 or feline$1 or goat$1 or hens or macque$1 or mice or monkey$1 or (mouse adj2 model$1) or murine$1 or ovine or pig$1 or porcine or (non-human adj2 primate$1) or sheep or rabbit$1 or rat or rats or rattus or rhesus or rodent$1 or zebrafish).ti.	168850
28	26 not 27	27
29	limit 28 to yr="1980 -Current"	27
30	(2024* or 2025*).up,yr.	301471
31	29 and 30	1
**CINAHL**		
S1	(MH "Fertilization in Vitro") OR (MH "Reproduction Techniques") OR (MH "Infertility") OR (MH "Prepregnancy Care")	25,209
S2	((TI "Intracytoplasmic sperm injection#" OR AB "Intracytoplasmic sperm injection#" OR SU "Intracytoplasmic sperm injection#") OR (TI "Intra-cytoplasmic sperm injection#" OR AB "Intra-cytoplasmic sperm injection#" OR SU "Intra-cytoplasmic sperm injection#") OR (TI icsi OR AB icsi OR SU icsi) OR (TI "in vitro fertili*" OR AB "in vitro fertili*" OR SU "in vitro fertili*") OR (TI "invitro fertili*" OR AB "invitro fertili*" OR SU "invitro fertili*") OR (TI ivf OR AB ivf OR SU ivf) OR ((TI "assisti* reproduct*" OR AB "assisti* reproduct*" OR SU "assisti* reproduct*") W1 ((TI therap* OR AB therap* OR SU therap*) OR (TI treatment OR AB treatment OR SU treatment) OR (TI technolog* OR AB technolog* OR SU technolog*)))) OR (((TI pre-pregnan*) OR (TI prepregnan*) OR (TI pre-concept*) OR (TI preconcept*)) AND ((TI care) OR (TI healthcare) OR (TI service#) OR (TI therap*) OR (TI treatment) OR (TI surg*) OR (TI program*) OR (TI intervention))) ,kf. OR (((AB pre-pregnan*) OR (AB prepregnan*) OR (AB pre-concept*) OR (AB preconcept*)) N5 ((AB care) OR (AB healthcare) OR (AB service#) OR (AB therap*) OR (AB treatment) OR (AB surg*) OR (AB program*) OR (AB intervention))) ((MH "reproductive techniques, assisted") OR (MH "fertilization in vitro+")	12,199
S3	S1 OR S2	28,637
S4	(MH "Obesity") OR (MH "Obesity, Maternal") OR (MH "Obesity, Morbid") OR (MH "Polycystic Ovary Syndrome")	104,837
S5	((TI obes* OR AB obes* OR SU obes*) OR (TI overweight OR AB overweight OR SU overweight)) OR ((TI "polycystic ovar* syndrome") OR (TI "poly-cystic ovar* syndrome") OR (TI pcos))	171,185
S6	S4 OR S5	172,340
S7	(MH "Obesity/DH/DT/TH/SU")	15,453
S8	((TI bodyweight OR AB bodyweight OR SU bodyweight) OR (TI weight OR AB weight OR SU weight)) N3 ((TI loss OR AB loss OR SU loss) OR (TI losing OR AB losing OR SU losing) OR (TI lose OR AB lose OR SU lose) OR (TI lost OR AB lost OR SU lost) OR (TI reduc* OR AB reduc* OR SU reduc*) OR (TI manage* OR AB manage* OR SU manage*) OR (TI control OR AB control OR SU control))) OR ((TI bmi) OR (TI "body mass index"))	78,350
S9	(MH "Diet Therapy") OR (MH "DASH Diet") OR (MH "Diet, Ketogenic") OR (MH "Diet, Nordic") OR (MH "Diet, Paleolithic") OR (MH "Diet, Reducing") OR (MH "Restricted Diet+") OR (MH "Obesity/DT/DH/TH") OR (MH "Weight Reduction Programs") OR (MH "Antiobesity Agents+") OR (MH "Bariatric Surgery+") OR (MH "Therapeutic Exercise+") OR (MH "Exercise+") OR (MH "Life Style Changes") OR (MH "Weight Loss")	232,569
S10	(((TI "low* fat" OR AB "low* fat" OR SU "low* fat") OR (TI "reduc* fat#" OR AB "reduc* fat#" OR SU "reduc* fat#") OR (TI "low* calorie*" OR AB "low*	69,826

	calorie*" OR SU "low* calorie*") OR (TI "reduc* calorie*" OR AB "reduc* calorie*" OR SU "reduc* calorie*") OR (TI "low* energy" OR AB "low* energy" OR SU "low* energy") OR (TI "reduc* energy" OR AB "reduc* energy" OR SU "reduc* energy") OR (TI hypocalor* OR AB hypocalor* OR SU hypocalor*) OR (TI "calori* restrict*" OR AB "calori* restrict*" OR SU "calori* restrict*") OR (TI "calorie control*" OR AB "calorie control*" OR SU "calorie control*") OR (TI "low* glyc*" OR AB "low* glyc*" OR SU "low* glyc*") OR (TI "reduc* glyc*" OR AB "reduc* glyc*" OR SU "reduc* glyc*") OR (TI "low* carbohydrate#" OR AB "low* carbohydrate#" OR SU "low* carbohydrate#") OR (TI "reduc* carbohydrate#" OR AB "reduc* carbohydrate#" OR SU "reduc* carbohydrate#") OR (TI "high* protein#" OR AB "high* protein#" OR SU "high* protein#") OR (TI "increas* protein#" OR AB "increas* protein#" OR SU "increas* protein#") OR (TI paleo* OR AB paleo* OR SU paleo*) OR (TI "intermittent fasting" OR AB "intermittent fasting" OR SU "intermittent fasting") OR (TI "meal replacement#" OR AB "meal replacement#" OR SU "meal replacement#") OR (TI "replacement meal#" OR AB "replacement meal#" OR SU "replacement meal#")) N2 ((TI diet# OR AB diet# OR SU diet#) OR (TI plan* OR AB plan* OR SU plan*) OR (TI regim* OR AB regim* OR SU regim*) OR (TI program* OR AB program* OR SU program*) OR (TI intervention# OR AB intervention# OR SU intervention#))) OR (((TI "weight loss" OR AB "weight loss" OR SU "weight loss") OR (TI "weight control" OR AB "weight control" OR SU "weight control") OR (TI "weight management" OR AB "weight management" OR SU "weight management") OR (TI "weight reduction" OR AB "weight reduction" OR SU "weight reduction")) N2 ((TI diet# OR AB diet# OR SU diet#) OR (TI plan* OR AB plan* OR SU plan*) OR (TI regim* OR AB regim* OR SU regim*) OR (TI program* OR AB program* OR SU program*) OR (TI intervention# OR AB intervention# OR SU intervention#))) OR (((TI antiobesity) OR (TI anti-obesity)) N3 ((TI medication#) OR (TI medicine#) OR (TI drug#) OR (TI agent#))) OR ((TI liraglutide OR AB liraglutide OR SU liraglutide) OR (TI saxenda OR AB saxenda OR SU saxenda) OR (TI victoza OR AB victoza OR SU victoza) OR (TI semaglutide OR AB semaglutide OR SU semaglutide) OR (TI ozempic OR AB ozempic OR SU ozempic) OR (TI wegovy OR AB wegovy OR SU wegovy) OR (TI tirzepatide OR AB tirzepatide OR SU tirzepatide) OR (TI exenatide OR AB exenatide OR SU exenatide) OR (TI bydureon OR AB bydureon OR SU bydureon) OR (TI byetta OR AB byetta OR SU byetta) OR (TI lixisenatide OR AB lixisenatide OR SU lixisenatide) OR (TI albiglutide OR AB albiglutide OR SU albiglutide) OR (TI tanzeum OR AB tanzeum OR SU tanzeum) OR (TI eperzan OR AB eperzan OR SU eperzan) OR (TI dulaglutide OR AB dulaglutide OR SU dulaglutide) OR (TI trulicity OR AB trulicity OR SU trulicity) OR (TI phentermine OR AB phentermine OR SU phentermine) OR (TI adipex-p OR AB adipex-p OR SU adipex-p) OR (TI suprenza OR AB suprenza OR SU suprenza) OR (TI topiramate OR AB topiramate OR SU topiramate) OR (TI topamax OR AB topamax OR SU topamax) OR (TI qsymia OR AB qsymia OR SU qsymia) OR (TI lorcaserin OR AB lorcaserin OR SU lorcaserin) OR (TI belviq OR AB belviq OR SU belviq) OR ((TI naltrexone OR AB naltrexone OR SU naltrexone) AND (TI bupropion OR AB bupropion OR SU bupropion)) OR (TI sibutramine OR AB sibutramine OR SU sibutramine) OR (TI rimonabant OR AB rimonabant OR SU rimonabant) OR (TI benzphetamine OR AB benzphetamine OR SU benzphetamine) OR (TI diethylpropion OR AB diethylpropion OR SU diethylpropion) OR (TI tenuate OR AB tenuate OR SU tenuate) OR (TI phendimetrazine OR AB phendimetrazine OR SU phendimetrazine) OR (TI bontril OR AB bontril OR SU bontril) OR (TI orlistat OR AB orlistat OR SU orlistat) OR (TI xenical OR AB xenical OR SU xenical)) OR (((TI lifestyle OR AB lifestyle OR SU lifestyle) OR (TI "life style" OR AB "life style" OR SU "life style")) N2 ((TI plan* OR AB plan* OR SU plan*) OR (TI regim* OR AB regim* OR SU regim*) OR (TI program* OR AB program* OR SU program*) OR (TI intervention# OR AB intervention# OR SU intervention#) OR (TI train* OR AB train* OR SU train*) OR (TI group# OR AB group# OR SU group#) OR (TI class* OR AB class* OR SU class*))) OR (((TI exercise OR AB exercise OR SU exercise) OR (TI "physical activity" OR AB "physical activity" OR SU "physical activity")) N2 ((TI plan* OR AB plan* OR SU plan*) OR (TI regim* OR AB regim* OR SU regim*) OR (TI program* OR AB program* OR SU program*) OR (TI intervention# OR AB intervention# OR SU intervention#) OR (TI train* OR AB train* OR SU train*) OR (TI group# OR AB group# OR SU group#) OR (TI class* OR AB class* OR SU class*)))	
S11	((TI "obesity surg*") OR (TI "metabolic surg*") OR (TI "weight loss surg*") OR (TI "gastric sleeve") OR (TI "intragastric sleeve") OR (TI "sleeve gastr*") OR (TI "ileal sleeve") OR (TI "gastric band*") OR (TI "intragastric band*") OR (TI "gastric bypass*") OR (TI "intragastric bypass*") OR (TI "jejuno*	9,745

	bypass*") OR (TI "ileal bypass*") OR (TI "gastric balloon*") OR (TI "intragastric balloon*") OR (TI roux-en-y) OR (TI "biliopancreatic diversion") OR (TI "bilio-pancreatic diversion") OR (TI "duodenal bypass*")) or ((TI "bariatric surg*") NOT ((TI nonbariatric) OR (TI non-bariatric)))	
S12	S7 OR S8 OR S9 OR S10 OR S11	301,768
S13	(MH "Pregnancy") OR (MH "Pregnancy Outcomes") OR (MH "Birth Rate")	229,950
S14	((TI pregnancy) OR (TI pregnancies) OR (TI pregnant)) OR ((AB pregnancy) N2 ((AB rate#) OR (AB clinical) OR (AB outcome#) OR (AB ongoing) OR (AB successful) OR (AB "time to"))) OR ((TI "live birth#" OR AB "live birth#" OR SU "live birth#") OR (TI "birth rate" OR AB "birth rate" OR SU "birth rate")) OR ((TI stillbirth# OR AB stillbirth# OR SU stillbirth#) OR (TI "still birth#" OR AB "still birth#" OR SU "still birth#") OR (((TI foetus OR AB foetus OR SU foetus) OR (TI fetus OR AB fetus OR SU fetus) OR (TI foetal OR AB foetal OR SU foetal) OR (TI fetal OR AB fetal OR SU fetal) OR (TI pregnan* OR AB pregnan* OR SU pregnan*)) W1 ((TI death# OR AB death# OR SU death#) OR (TI mortality OR AB mortality OR SU mortality) OR (TI loss OR AB loss OR SU loss))) OR (TI miscarriage# OR AB miscarriage# OR SU miscarriage#) OR (TI "spontaneous abortion#" OR AB "spontaneous abortion#" OR SU "spontaneous abortion#"))	116,984
S15	S13 OR S14	264,039
S16	S3 AND S6 AND S12 AND S15	356
S17	S3 AND S6 AND S12 AND S15 Limiters - Publication Date: 19800101-20241231	356
S20	S17 OR S19	36
S19	S16 AND S18	36
S18	(ZD "2024*") or (ZD "2025*")	329,143
S17	S3 AND S6 AND S12 AND S15 Limiters - Publication Date: 20240101-20251231	34
S16	S3 AND S6 AND S12 AND S15	378
S15	S13 OR S14	270,155
S14	((TI pregnancy) OR (TI pregnancies) OR (TI pregnant)) OR ((AB pregnancy) N2 ((AB rate#) OR (AB clinical) OR (AB outcome#) OR (AB ongoing) OR (AB successful) OR (AB "time to"))) OR ((TI "live birth#" OR AB "live birth#" OR SU "live birth#") OR (TI "birth rate" OR AB "birth rate" OR SU "birth rate")) OR ((TI stillbirth# OR AB stillbirth# OR SU stillbirth#) OR (TI "still birth#" OR AB "still birth#" OR SU "still birth#") OR (((TI foetus OR AB foetus OR SU foetus) OR (TI fetus OR AB fetus OR SU fetus) OR (TI foetal OR AB foetal OR SU foetal) OR (TI fetal OR AB fetal OR SU fetal) OR (TI pregnan* OR AB pregnan* OR SU pregnan*)) W1 ((TI death# OR AB death# OR SU death#) OR (TI mortality OR AB mortality OR SU mortality) OR (TI loss OR AB loss OR SU loss))) OR (TI miscarriage# OR AB miscarriage# OR SU miscarriage#) OR (TI "spontaneous abortion#" OR AB "spontaneous abortion#" OR SU "spontaneous abortion#"))	122,590
S13	(MH "Pregnancy") OR (MH "Pregnancy Outcomes") OR (MH "Birth Rate")	232,146
S12	S7 OR S8 OR S9 OR S10 OR S11	310,981
S11	((TI "obesity surg*") OR (TI "metabolic surg*") OR (TI "weight loss surg*") OR (TI "gastric sleeve") OR (TI "intragastric sleeve") OR (TI "sleeve gastr*") OR (TI "ileal sleeve") OR (TI "gastric band*") OR (TI "intragastric band*") OR (TI "gastric bypass*") OR (TI "intragastric bypass*") OR (TI "jejuno* bypass*") OR (TI "ileal bypass*") OR (TI "gastric balloon*") OR (TI "intragastric balloon*") OR (TI roux-en-y) OR (TI "biliopancreatic diversion") OR (TI "bilio-pancreatic diversion") OR (TI "duodenal bypass*")) or ((TI "bariatric surg*") NOT ((TI nonbariatric) OR (TI non-bariatric)))	9,160
S10	(((TI "low* fat" OR AB "low* fat" OR SU "low* fat") OR (TI "reduc* fat#" OR AB "reduc* fat#" OR SU "reduc* fat#") OR (TI "low* calorie*" OR AB "low* calorie*" OR SU "low* calorie*") OR (TI "reduc* calorie*" OR AB "reduc* calorie*" OR SU "reduc* calorie*") OR (TI "low* energy" OR AB "low* energy" OR SU "low* energy") OR (TI "reduc* energy" OR AB "reduc* energy" OR SU "reduc* energy") OR (TI hypocalor* OR AB hypocalor* OR SU hypocalor*) OR (TI "calori* restrict*" OR AB "calori* restrict*" OR SU "calori* restrict*") OR (TI "calorie control*" OR AB "calorie control*" OR SU "calorie control*") OR (TI "low* glyc*" OR AB "low* glyc*" OR SU "low* glyc*") OR (TI "reduc* glyc*" OR AB "reduc* glyc*" OR SU "reduc* glyc*") OR (TI "low* carbohydrate#" OR AB "low* carbohydrate#" OR SU "low* carbohydrate#") OR (TI "reduc* carbohydrate#" OR AB "reduc* carbohydrate#" OR SU "reduc* carbohydrate#") OR (TI "high* protein#" OR AB "high* protein#" OR SU "high* protein#") OR (TI "increas* protein#" OR AB "increas* protein#" OR SU "increas* protein#") OR (TI paleo* OR AB paleo* OR SU paleo*) OR (TI "intermittent fasting" OR AB "intermittent fasting" OR SU "intermittent fasting") OR (TI "meal replacement#" OR AB	72,906

	"meal replacement#" OR SU "meal replacement#") OR (TI "replacement meal#" OR AB "replacement meal#" OR SU "replacement meal#")) N2 ((TI diet# OR AB diet# OR SU diet#) OR (TI plan* OR AB plan* OR SU plan*) OR (TI regim* OR AB regim* OR SU regim*) OR (TI program* OR AB program* OR SU program*) OR (TI intervention# OR AB intervention# OR SU intervention#))) OR (((TI "weight loss" OR AB "weight loss" OR SU "weight loss") OR (TI "weight control" OR AB "weight control" OR SU "weight control") OR (TI "weight management" OR AB "weight management" OR SU "weight management") OR (TI "weight reduction" OR AB "weight reduction" OR SU "weight reduction")) N2 ((TI diet# OR AB diet# OR SU diet#) OR (TI plan* OR AB plan* OR SU plan*) OR (TI regim* OR AB regim* OR SU regim*) OR (TI program* OR AB program* OR SU program*) OR (TI intervention# OR AB intervention# OR SU intervention#))) OR (((TI antiobesity) OR (TI anti-obesity)) N3 ((TI medication#) OR (TI medicine#) OR (TI drug#) OR (TI agent#))) OR ((TI liraglutide OR AB liraglutide OR SU liraglutide) OR (TI saxenda OR AB saxenda OR SU saxenda) OR (TI victoza OR AB victoza OR SU victoza) OR (TI semaglutide OR AB semaglutide OR SU semaglutide) OR (TI ozempic OR AB ozempic OR SU ozempic) OR (TI wegovy OR AB wegovy OR SU wegovy) OR (TI tirzepatide OR AB tirzepatide OR SU tirzepatide) OR (TI exenatide OR AB exenatide OR SU exenatide) OR (TI bydureon OR AB bydureon OR SU bydureon) OR (TI byetta OR AB byetta OR SU byetta) OR (TI lixisenatide OR AB lixisenatide OR SU lixisenatide) OR (TI albiglutide OR AB albiglutide OR SU albiglutide) OR (TI tanzeum OR AB tanzeum OR SU tanzeum) OR (TI eperzan OR AB eperzan OR SU eperzan) OR (TI dulaglutide OR AB dulaglutide OR SU dulaglutide) OR (TI trulicity OR AB trulicity OR SU trulicity) OR (TI phentermine OR AB phentermine OR SU phentermine) OR (TI adipex-p OR AB adipex-p OR SU adipex-p) OR (TI suprenza OR AB suprenza OR SU suprenza) OR (TI topiramate OR AB topiramate OR SU topiramate) OR (TI topamax OR AB topamax OR SU topamax) OR (TI qsymia OR AB qsymia OR SU qsymia) OR (TI lorcaserin OR AB lorcaserin OR SU lorcaserin) OR (TI belviq OR AB belviq OR SU belviq) OR ((TI naltrexone OR AB naltrexone OR SU naltrexone) AND (TI bupropion OR AB bupropion OR SU bupropion)) OR (TI sibutramine OR AB sibutramine OR SU sibutramine) OR (TI rimonabant OR AB rimonabant OR SU rimonabant) OR (TI benzphetamine OR AB benzphetamine OR SU benzphetamine) OR (TI diethylpropion OR AB diethylpropion OR SU diethylpropion) OR (TI tenuate OR AB tenuate OR SU tenuate) OR (TI phendimetrazine OR AB phendimetrazine OR SU phendimetrazine) OR (TI bontril OR AB bontril OR SU bontril) OR (TI orlistat OR AB orlistat OR SU orlistat) OR (TI xenical OR AB xenical OR SU xenical)) OR (((TI lifestyle OR AB lifestyle OR SU lifestyle) OR (TI "life style" OR AB "life style" OR SU "life style")) N2 ((TI plan* OR AB plan* OR SU plan*) OR (TI regim* OR AB regim* OR SU regim*) OR (TI program* OR AB program* OR SU program*) OR (TI intervention# OR AB intervention# OR SU intervention#) OR (TI train* OR AB train* OR SU train*) OR (TI group# OR AB group# OR SU group#) OR (TI class* OR AB class* OR SU class*))) OR (((TI exercise OR AB exercise OR SU exercise) OR (TI "physical activity" OR AB "physical activity" OR SU "physical activity")) N2 ((TI plan* OR AB plan* OR SU plan*) OR (TI regim* OR AB regim* OR SU regim*) OR (TI program* OR AB program* OR SU program*) OR (TI intervention# OR AB intervention# OR SU intervention#) OR (TI train* OR AB train* OR SU train*) OR (TI group# OR AB group# OR SU group#) OR (TI class* OR AB class* OR SU class*)))	
S9	(MH "Diet Therapy") OR (MH "DASH Diet") OR (MH "Diet, Ketogenic") OR (MH "Diet, Nordic") OR (MH "Diet, Paleolithic") OR (MH "Diet, Reducing") OR (MH "Restricted Diet+") OR (MH "Obesity/DT/DH/TH") OR (MH "Weight Reduction Programs") OR (MH "Antiobesity Agents+") OR (MH "Bariatric Surgery+") OR (MH "Therapeutic Exercise+") OR (MH "Exercise+") OR (MH "Life Style Changes") OR (MH "Weight Loss")	238,913
S8	((TI bodyweight OR AB bodyweight OR SU bodyweight) OR (TI weight OR AB weight OR SU weight)) N3 ((TI loss OR AB loss OR SU loss) OR (TI losing OR AB losing OR SU losing) OR (TI lose OR AB lose OR SU lose) OR (TI lost OR AB lost OR SU lost) OR (TI reduc* OR AB reduc* OR SU reduc*) OR (TI manage* OR AB manage* OR SU manage*) OR (TI control OR AB control OR SU control))) OR ((TI bmi) OR (TI "body mass index"))	81,238
S7	(MH "Obesity/DH/DT/TH/SU")	15,974
S6	S4 OR S5	178,654
S5	((TI obes* OR AB obes* OR SU obes*) OR (TI overweight OR AB overweight OR SU overweight)) OR ((TI "polycystic ovar* syndrome") OR (TI "poly-cystic ovar* syndrome") OR (TI pcos))	177,449
S4	(MH "Obesity") OR (MH "Obesity, Maternal") OR (MH "Obesity, Morbid") OR (MH "Polycystic Ovary Syndrome")	107,247

S3	S1 OR S2	29,587
S2	((TI "Intracytoplasmic sperm injection#" OR AB "Intracytoplasmic sperm injection#" OR SU "Intracytoplasmic sperm injection#") OR (TI "Intra-cytoplasmic sperm injection#" OR AB "Intra-cytoplasmic sperm injection#" OR SU "Intra-cytoplasmic sperm injection#") OR (TI icsi OR AB icsi OR SU icsi) OR (TI "in vitro fertili*" OR AB "in vitro fertili*" OR SU "in vitro fertili*") OR (TI "invitro fertili*" OR AB "invitro fertili*" OR SU "invitro fertili*") OR (TI ivf OR AB ivf OR SU ivf) OR ((TI "assisti* reproduct*" OR AB "assisti* reproduct*" OR SU "assisti* reproduct*") W1 ((TI therap* OR AB therap* OR SU therap*) OR (TI treatment OR AB treatment OR SU treatment) OR (TI technolog* OR AB technolog* OR SU technolog*)))) OR (((TI pre-pregnan*) OR (TI prepregnan*) OR (TI pre-concept*) OR (TI preconcept*)) AND ((TI care) OR (TI healthcare) OR (TI service#) OR (TI therap*) OR (TI treatment) OR (TI surg*) OR (TI program*) OR (TI intervention))) ,kf. OR (((AB pre-pregnan*) OR (AB prepregnan*) OR (AB pre-concept*) OR (AB preconcept*)) N5 ((AB care) OR (AB healthcare) OR (AB service#) OR (AB therap*) OR (AB treatment) OR (AB surg*) OR (AB program*) OR (AB intervention))) ((MH "reproductive techniques, assisted") OR (MH "fertilization in vitro+")	12,664
S1	(MH "Fertilization in Vitro") OR (MH "Reproduction Techniques") OR (MH "Infertility") OR (MH "Prepregnancy Care")	25,871
**Cochrane**		
1	[mh ^"reproductive techniques, assisted"] OR [mh "fertilization in vitro"] OR [mh ^"Infertility, Female"] OR [mh ^"Preconception Care"]	4599
2	(("Intracytoplasmic sperm" NEXT injection?):ti,ab,kw OR ("Intra-cytoplasmic sperm" NEXT injection?):ti,ab,kw OR icsi:ti,ab,kw OR ("in vitro" NEXT fertili*):ti,ab,kw OR ("invitro" NEXT fertili*):ti,ab,kw OR ivf:ti,ab,kw OR ((assisti* NEXT reproduct*):ti,ab,kw NEXT (therap*:ti,ab,kw OR treatment:ti,ab,kw OR technolog*:ti,ab,kw))) OR ((pre-pregnan*:ti OR prepregnan*:ti OR pre-concept*:ti OR preconcept*:ti) AND (care:ti OR healthcare:ti OR service?:ti OR therap*:ti OR treatment:ti OR surg*:ti OR program*:ti OR intervention:ti)) OR ((pre-pregnan*:ab OR prepregnan*:ab OR pre-concept*:ab OR preconcept*:ab) NEAR/5 (care:ab OR healthcare:ab OR service?:ab OR therap*:ab OR treatment:ab OR surg*:ab OR program*:ab OR intervention:ab))	10694
3	#1 OR #2	12302
4	[mh ^overweight] OR [mh ^obesity] OR [mh ^"obesity, abdominal"] OR [mh ^"obesity, maternal"] OR [mh ^"obesity, morbid"] OR [mh ^"Polycystic Ovary Syndrome"]	25649
5	(obes*:ti,ab,kw OR overweight:ti,ab,kw) OR (("polycystic" NEXT ovar* NEXT "syndrome"):ti OR ("poly-cystic" NEXT ovar* NEXT "syndrome"):ti OR pcos:ti)	67322
6	#4 OR #5	67545
7	MeSH descriptor: [Obesity] explode all trees and with qualifier(s): [drug therapy - DT, therapy - TH, surgery - SU, diet therapy - DH]	10117
8	[mh ^"body weight changes"] OR [mh ^"weight loss"] OR [mh ^"diet therapy"] OR [mh ^"caloric restriction"] OR [mh "diet, carbohydrate-restricted"] OR [mh ^"diet, fat-restricted"] OR [mh "diet, high-protein"] OR [mh ^"diet, reducing"] OR [mh ^"dietary approaches to stop hypertension"] OR [mh Exercise] OR [mh "Exercise Therapy"] OR [mh ^"Obesity Management"] OR [mh "Anti-Obesity Agents"] OR [mh "bariatric surgery"]	62086
9	((bodyweight:ti,ab,kw OR weight:ti,ab,kw) NEAR/3 (loss:ti,ab,kw OR losing:ti,ab,kw OR lose:ti,ab,kw OR lost:ti,ab,kw OR reduc*:ti,ab,kw OR manage*:ti,ab,kw OR control:ti,ab,kw)) OR (((low* NEXT "fat"):ti,ab,kw OR (reduc* NEXT fat?):ti,ab,kw OR (low* NEXT calorie*):ti,ab,kw OR (reduc* NEXT calorie*):ti,ab,kw OR (low* NEXT "energy"):ti,ab,kw OR (reduc* NEXT "energy"):ti,ab,kw OR hypocalor*:ti,ab,kw OR (calori* NEXT restrict*):ti,ab,kw OR ("calorie" NEXT control*):ti,ab,kw OR (low* NEXT glyc*):ti,ab,kw OR (reduc* NEXT glyc*):ti,ab,kw OR (low* NEXT carbohydrate?):ti,ab,kw OR (reduc* NEXT carbohydrate?):ti,ab,kw OR (high* NEXT protein?):ti,ab,kw OR (increas* NEXT protein?):ti,ab,kw OR keto*:ti,ab,kw OR paleo*:ti,ab,kw OR "intermittent fasting":ti,ab,kw OR ("meal" NEXT replacement?):ti,ab,kw OR ("replacement" NEXT meal?):ti,ab,kw) NEAR/2 (diet?:ti,ab,kw OR plan*:ti,ab,kw OR regim*:ti,ab,kw OR program*:ti,ab,kw OR intervention?:ti,ab,kw)) OR (("weight loss":ti,ab,kw OR "weight control":ti,ab,kw OR "weight management":ti,ab,kw OR "weight reduction":ti,ab,kw) NEAR/2 (diet?:ti,ab,kw OR plan*:ti,ab,kw OR regim*:ti,ab,kw OR program*:ti,ab,kw OR intervention?:ti,ab,kw OR group?:ti,ab,kw OR class*:ti,ab,kw)) OR ((exercise:ti,ab,kw OR "physical activity":ti,ab,kw) NEAR/2 (plan*:ti,ab,kw	104919

	OR regim*:ti,ab,kw OR program*:ti,ab,kw OR intervention?:ti,ab,kw OR train*:ti,ab,kw OR group?:ti,ab,kw OR class*:ti,ab,kw)) OR ((lifestyle:ti,ab,kw OR "life style":ti,ab,kw) NEAR/2 (plan*:ti,ab,kw OR regim*:ti,ab,kw OR program*:ti,ab,kw OR intervention?:ti,ab,kw OR train*:ti,ab,kw OR group?:ti,ab,kw OR class*:ti,ab,kw)) OR ((antiobesity:ti OR anti-obesity:ti) NEAR/3 (medication?:ti OR medicine?:ti OR drug?:ti OR agent?:ti)) OR (liraglutide:ti,ab,kw OR saxenda:ti,ab,kw OR victoza:ti,ab,kw OR semaglutide:ti,ab,kw OR ozempic:ti,ab,kw OR wegovy:ti,ab,kw OR tirzepatide:ti,ab,kw OR exenatide:ti,ab,kw OR bydureon:ti,ab,kw OR byetta:ti,ab,kw OR lixisenatide:ti,ab,kw OR albiglutide:ti,ab,kw OR tanzeum:ti,ab,kw OR eperzan:ti,ab,kw OR dulaglutide:ti,ab,kw OR trulicity:ti,ab,kw OR phentermine:ti,ab,kw OR adipex-p:ti,ab,kw OR suprenza:ti,ab,kw OR topiramate:ti,ab,kw OR topamax:ti,ab,kw OR qsymia:ti,ab,kw OR lorcaserin:ti,ab,kw OR belviq:ti,ab,kw OR (naltrexone:ti,ab,kw AND bupropion:ti,ab,kw) OR sibutramine:ti,ab,kw OR rimonabant:ti,ab,kw OR benzphetamine:ti,ab,kw OR diethylpropion:ti,ab,kw OR tenuate:ti,ab,kw OR phendimetrazine:ti,ab,kw OR bontril:ti,ab,kw OR orlistat:ti,ab,kw OR xenical:ti,ab,kw) OR (("bariatric" NEXT surg*):ti,ab,kw OR ("obesity" NEXT surg*):ti,ab,kw OR ("metabolic" NEXT surg*):ti,ab,kw OR ("weight loss" NEXT surg*):ti,ab,kw OR "gastric sleeve":ti,ab,kw OR "intragastric sleeve":ti,ab,kw OR ("sleeve" NEXT gastr*):ti,ab,kw OR "ileal sleeve":ti,ab,kw OR ("gastric" NEXT band*):ti,ab,kw OR ("intragastric" NEXT band*):ti,ab,kw OR ("gastric" NEXT bypass*):ti,ab,kw OR ("intragastric" NEXT bypass*):ti,ab,kw OR (jejuno* NEXT bypass*):ti,ab,kw OR ("ileal" NEXT bypass*):ti,ab,kw OR ("gastric" NEXT balloon*):ti,ab,kw OR ("intragastric" NEXT balloon*):ti,ab,kw OR roux-en-y:ti,ab,kw OR "biliopancreatic diversion":ti,ab,kw OR "bilio-pancreatic diversion":ti,ab,kw OR ("duodenal" NEXT bypass*):ti,ab,kw)	
10	#7 OR #8 OR #9	138921
11	#3 AND #6 AND #10 with Publication Year from 1980 to 2025, in Trials	201

Table S2: List of records excluded at full-text screening stage

Study ID	Identified record/reference	Reason for exclusion
Muesella 2012	Musella M, Milone M, Bellini M, et al. Effect of bariatric surgery on obesity-related infertility. Surg Obes Relat Dis.2012;8(4):445-9.	Not seeking IVF
Zhang 2021	Zhang YF, Luo HN, Shi R, et al. [Effect of body mass index on the assisted reproductive outcome of frozen-thawed embryo transfer in patients with polycystic ovary syndrome]. Zhonghua Fu Chan Ke Za Zhi. 2021;56(4):257-63.	No weight loss intervention
Lyo 2024	Lyo V. Comment on: The impact of metabolic surgery on natural conception rates in women with infertility, obesity, and polycystic ovary syndrome: a retrospective study. Surg Obes Relat Dis. 2024;20(3):243-244.	Commentary
Sang 2022	Sang M, Wu Q, Tao Y, et al. Usage of mobile health interventions among overweight/obese PCOS patients undergoing assisted reproductive technology treatment during the COVID-19 pandemic. Gynecol Endocrinol. 2022;38(9):776-80.	Average BMI <27 kg/m^2
Cutler 2018	Cutler DA, Shaw AK, Pride SM, et al. A randomized controlled trial comparing lifestyle intervention to letrozole for ovulation in women with polycystic ovary syndrome: a study protocol. Trials. 2018;19(1):632.	Not seeking IVF
Chang 2020	Chang JJ, Lathi RB, Kim SH. A Retrospective Study Examining Phentermine on Preconception Weight Loss and Pregnancy Outcomes. Endocr Pract. 2020;26(9):990-6.	Data not available specifically for women seeking IVF
Jamal 2012<br><br>Gunay 2011	Jamal M, Gunay Y, Capper A, et al. Roux-en-Y gastric bypass ameliorates polycystic ovary syndrome and dramatically improves conception rates: a 9-year analysis. Surg Obes Relat Dis. 2012;8(4):440-4.<br><br>Gunay Y, Jamal MK, Capper A, et al. Roux-en-Y gastric bypass (RYGB) ameliorates polycystic ovarian syndrome (PCOS) and dramatically improves conception rates: A nine-year analysis. SOARD. 2011;7(3):343-344.	Not seeking IVF
Liu 2024	Liu X, Chen P, Wang M, et al. Association between pre-gravid body mass index and clinical outcomes in in vitro fertilization: a multicentered retrospective cohort study. BMC Pregnancy Childbirth. 2024;24(1):469.	No weight loss intervention
Crosigniani 2003	Crosignani PG, Colombo M, Vegetti W, et al. Overweight and obese anovulatory patients with polycystic ovaries: parallel improvements in anthropometric indices, ovarian physiology and fertility rate induced by diet. Hum Reprod. 2003;18(9):1928-32.	Not seeking IVF
LeBlanc 2016<br><br>Leblanc 2019<br><br>LeBlanc 2021<br><br>LeBlanc 2021	LeBlanc ES, Vesco KK, Funk KL, et al. Prepare, a randomized trial to promote and evaluate weight loss among overweight and obese women planning pregnancy: Study design and rationale. Contemp Clin Trials. 2016; 49: 174–80.<br><br>Leblanc ES, Smith N, Vesco K, et al. How does a prepregnancy weight loss program affect gestational diabetes risk? Diabetes 2019;68():<br><br>LeBlanc ES, Smith NX, Vesco, K, et al. Weight loss prior to pregnancy and subsequent gestational weight gain: Prepare, a randomized clinical trial. AJOG. 2021;224(1):99.e1-99.e14.<br><br>LeBlanc ES, Smith NX, Vesco KK, et al. Weight Loss Prior to Pregnancy and Early Gestational Glycemia: Prepare, a Randomized Clinical Trial. J Clin Endocrinol Metab. 2021;106(12):e5001-e10.	Not seeking IVF
Hathorn 2020<br><br>Corwin 2020	Hathorn K, McCarty TR, Corwin A, et al. 187 PREGNANCY AND ASSOCIATED OUTCOMES AMONG PATIENTS FOLLOWING SLEEVE GASTRECTOMY: A RETROSPECTIVE COMPARATIVE ANALYSIS OF BARIATRIC SURGERY PATIENTS FOLLOWED IN A REPRODUCTIVE INFERTILITY CLINIC. Gastroenterology 2020;158(6 Supplement 1):S-1501.<br><br>Corwin A, Hathorn K, McCarty T, et al. FERTILITY OUTCOMES AMONG PATIENTS FOLLOWING ROUX-EN-Y GASTRIC BYPASS: A RETROSPECTIVE COMPARATIVE ANALYSIS OF BARIATRIC	Published only as a conference abstract

Study ID	Identified record/reference	Reason for exclusion
	SURGERY PATIENTS FOLLOWED IN A REPRODUCTIVE INFERTILITY CLINIC. Gastroenterology 2020;158(6 Supplement 1):S-1560.	
Azami 2020	Azami S, Nourizadeh R, Mehrabi E, et al. Effect of Motivational Interviewing on Dietary Intake and Weight Changes Among Preconception Women With Overweight and Obesity: A Randomized Controlled Trial. Crescent J Med Biol. 2020;7(2):260-6.	Not seeking IVF
Hoek 2022	Hoek A, Wang Z, van Oers AM, et al. Effects of preconception weight loss after lifestyle intervention on fertility outcomes and pregnancy complications. Fertil Steril. 2022;118(3):456-62.	Wrong study design (review)
Baker 2020	Baker KM, Dodge LE, Thornton KL. IVF outcomes after bariatric surgery. Fertility and Sterility. 2020; 114(3 SUPPL): e300–e301.	Published only as a conference abstract
Santos-Ribeiro 2021	Santos-Ribeiro S, Rodrigues M, Bellver J, et al. P–787 Impact of delaying ART to promote weight loss: a large multicentre study accounting for the combined effect of female/male age and body mass index (BMI). Human Reproduction. 2021;36(Supplement_1).	No weight loss intervention
Ockhuijsen 2012	Ockhuijsen HD, Gamel CJ, van den Hoogen A, et al. Integrating preconceptional care into an IVF programme. J Adv Nurs. 2012;68(5):1156-65.	Wrong study design
Bojko 2022<br><br>Halperin 2022	Bojko A, Vaughan DA, Sakkas D, et al. An effective telehealth based multidisciplinary approach to weight loss in women with obesity seeking treatment for infertility. Fertility and Sterility. 2022; 118(4 Supplement): e31–e32.<br><br>Halperin F, Axelbaum J, Joshi P, et al. Women With Infertility Referred to Telehealth Obesity Treatment Show High Engagement and Lose Weight. Obesity. 2022; 30(Supplement 1): 273.	Published only as a conference abstract
Abdalmageed 2019	Abdalmageed OS, Farghaly TA, Abdelaleem AA, et al. Impact of Metformin on IVF Outcomes in Overweight and Obese Women With Polycystic Ovary Syndrome: A Randomized Double-Blind Controlled Trial. Reprod Sci. 2019; 26: 1336–42.	Metformin is not an approved weight loss drug and authors do not report weight outcomes. As a result, it is not clear if this can be considered a weight loss intervention.
Benito 2020	Benito E, Gómez-Martin JM, Vega-Piñero B, et al. Fertility and Pregnancy Outcomes in Women with Polycystic Ovary Syndrome Following Bariatric Surgery. J Clin Endocrinol Metab. 2020;105(9).	Mentions "seeking fertility", but does not mention IVF specifically.
Moore 2016	Moore AK, Rasmussen R, Sandberg J, et al. Intensive lifestyle intervention including emotionally-focused couples therapy leads to more pregnancies and weight loss in obese infertile couples. Fertility and Sterility 2016;106(Supplement 3):e101.	No full publication can be found. The abstract does not suggest women were seeking IVF because in the results says some conceived naturally, some with letrozole, and some with IUI.
Kaya 2016	Kaya Y, Kizilkaya Beji N, Aydin Y, et al. The effect of health-promoting lifestyle education on the treatment of unexplained female infertility. Eur J Obstet Gynecol Reprod Biol. 2016; 207: 109–14.	No obesity. Overweight was one of many potential inclusion criteria, average BMI was <27 kg/m^2, whilst data reported for the sample as a whole.
Tong 2022	Tong J, Xiang L, Niu Y, et al. Effect of orlistat intervention on in vitro fertilization/intracytoplasmic sperm injection outcome in overweight/obese infertile women. Gynecol Endocrinol. 2022; 38: 253–7.	Non-RCT which does not report weight outcomes
Jiskoot 2017<br><br>Dietz de Loos 2021<br><br>Van Der Ham 2022	Jiskoot G, de Loos AD, Timman R, et al. Changes in eating behavior through lifestyle treatment in women with polycystic ovary syndrome (PCOS): a randomized controlled trial. J Eat Disord. 2022; 10: 69.<br><br>Dietz de Loos ALP, Jiskoot G, Timman R, et al. Improvements in PCOS characteristics and phenotype severity during a randomized controlled lifestyle intervention. Reprod Biomed Online. 2021;43(2):298-309.<br><br>Van Der Ham K, Jiskoot G, Louwers Y, et al. Pregnancy Rate and Outcomes Following a Randomized Controlled Three-component Lifestyle Intervention in Women with PCOS. J Endocr Soc. 2022;6(Supplement 1):A686.	Not seeking IVF
Consalvo 2017	Consalvo V, Canero A, Salsano V. Bariatric Surgery and Infertility: A Prospective Study. Surg Technol Int. 2017;31:327-30.	Not seeking IVF

Study ID	Identified record/reference	Reason for exclusion
Sujan 2023	Sujan MAJ, Skarstad HMS, Rosvold G, et al. Randomised controlled trial of preconception lifestyle intervention on maternal and offspring health in people with increased risk of gestational diabetes: study protocol for the BEFORE THE BEGINNING trial. BMJ Open. 2023;13(10):e073572.	Not seeking IVF
Vigna 2024	Vigna L, Piontini A, Nicolosi, A, et al. Prospective observational study in women with overweight-obesity and infertility treated with a Very Low Calorie Ketogenic Diet. Obesity Facts 2024;17(Supplement 1):573-574.	Published only as a conference abstract
Valeti 2017	Valet M. Bariatric surgery improves fertility in pre-pregnant women. fertility, pregnancy and bariatric surgery. Obes Surg 2017;27(1 Supplement 1):538.	Cannot find full publication but does not seem to be IVF.
Silvestrim 2019	Silvestrim RL, Bos-Mikich A, Kulmann MIR, et al. The Effects of Overweight and Obesity on Assisted Reproduction Technology Outcomes. JBRA Assist Reprod. 2019; 23: 281–6.	Non-RCT which does not report weight outcomes
Musella 2011	Musella M, Milone M, Bellini M, et al. The Potential Role of Intragastric Balloon in the Treatment of Obese-Related Infertility: Personal Experience. Obes Surg. 2011; **21:** 426-30.	Not all women seeking IVF
Beena 2016	Beena MR, Thomas, K. Outcome of Interventional Programme on Quality of Life of Infertile Women with Polycystic Ovarian Syndrome. Int J Nurs Educ. 2016;8(2):27-33	Not seeking IVF
Karayiannis 2018<br><br>Karayiannis 2017	Karayiannis D, Kontogianni MD, Mendorou C, et al. Adherence to the Mediterranean diet and IVF success rate among non-obese women attempting fertility. Hum Reprod. 2018; 33: 494–502.<br><br>Karayiannis D, Kontogianni M, Mendorou C, et al. In vitro fertilization outcomes in relation to adherence to the Mediterranean diet among women from a fertility clinic. Hum Reprod. 2017; 32(Supplement 1): i295–i296.	No obesity
Legro 2015<br><br>Legro 2016<br><br>Legro 2014	Legro RS, Dodson WC, Kris-Etherton PM, et al. Randomized Controlled Trial of Preconception Interventions in Infertile Women With Polycystic Ovary Syndrome. J Clin Endocrinol Metab. 2015; 100: 4048–58.<br><br>Legro RS, Dodson WC, Kunselman AR, et al. Benefit of Delayed Fertility Therapy With Preconception Weight Loss Over Immediate Therapy in Obese Women With PCOS. J Clin Endocrinol Metab. 2016; 101: 2658-66.<br><br>Legro RS, Dodson WC, Kunselman AR, et al. Effects of preconception intervention on the PCOS phenotype, ovulation, and live birth rates: A multicenter, multi-phase RCT. Fertil Steril 2014;102(3 SUPPL. 1):e2.	Not seeking IVF
Miyako 2010	Miyako F, Sagiri T, Terumi H, et al. Effects of obesity and weight loss on Japanese infertile women. Obes Rev. 2010;11(SUPPL. 1):290	Published only as a conference abstract
Kjotrod 2004	Kjøtrød SB, von Düring V, Carlsen SM. Metformin treatment before IVF/ICSI in women with polycystic ovary syndrome; a prospective, randomized, double blind study. Hum Reprod. 2004;19(6):1315-22.	Metformin is not an approved weight loss drug, although it sometimes leads to weight loss.
Oostingh 2020<br><br>Steegers-Theunissen 2018	Oostingh EC, Koster MPH, van Dijk MR, et al. First effective mHealth nutrition and lifestyle coaching program for subfertile couples undergoing in vitro fertilization treatment: a single-blinded multicenter randomized controlled trial. Fertil Steril. 2020; 114: 945–54.<br><br>Steegers-Theunissen RPM. Preconceptional personalised mHealth lifestyle coaching:\rFirst results of a randomized controlled trial in couples undergoing IVF/ICSI treatment. Hum Reprod 2018; 33(Supplement 1): i74–i75.	No weight loss intervention
Rafael 2023	Rafael F, Rodrigues MD, Bellver J, et al. The combined effect of BMI and age on ART outcomes. Hum Reprod. 2023;38(5):886-94.	No weight loss intervention
Chan 2023	Chan SY, Barton SJ, Loy SL, et al. Time-to-conception and clinical pregnancy rate with a myo-inositol, probiotics, and micronutrient supplement: secondary outcomes of the NiPPeR randomized trial. Fertil Steril. 2023; 119: 1031-42.	Not seeking IVF

Study ID	Identified record/reference	Reason for exclusion
Infante Vincenzo 2009	Infante Vincenzo VI, D'Aniello Gemma DG, Irollo AMIA, et al. Preventive BMI reduction increase ovarian response and pregnancy rate in Myo-inositol and r-FSH in stimulated cycles of anovulatory obese women. Mol Hum Reprod. 2009; 24(SUPPL. 1): i3–i4	Published only as a conference abstract
Christofolini 2014	Christofolini J, Bianco B, Santos G, et al. Bariatric surgery influences the number and quality of oocytes in patients submitted to assisted reproduction techniques. Obesity (Silver Spring). 2014;22(3):939-42.	Not seeking IVF
Samarasinghe 2024	Samarasinghe SNS, Leca B, Alabdulkader S, et al. Bariatric surgery for spontaneous ovulation in women living with polycystic ovary syndrome: the BAMBINI multicentre, open-label, randomised controlled trial. Lancet. 2024;403(10443):2489-503	Not seeking IVF
Phelan 2023<br><br>Phelan 2022	Phelan S, Jelalian E, Coustan D, et al. Randomized controlled trial of prepregnancy lifestyle intervention to reduce recurrence of gestational diabetes mellitus. Am J Obstet Gynecol. 2023;229(2):158.e1-.e14.<br><br>Phelan S, Jelalian E, Coustan D, et al. Pre-pregnancy Lifestyle Intervention to Reduce the Recurrence of Gestational Diabetes. Obesity. 2022;30(Supplement 1):156.	Not seeking IVF
Yang 2022	Yang C, Yang S, Zheng W, et al. Effect of a 60-day weight reduction intervention prior to IVF/ICSI on perinatal outcomes in overweight or obese infertile women. Front Endocrinol (Lausanne). 2022; 13: 1062790.	Included women who already gave birth, therefore, pregnancy and live births were not an outcome.
Sancak 2019	Sancak S, Celer O, Cirak E, et al. Timing of Gestation After Laparoscopic Sleeve Gastrectomy (LSG): Does It Influence Obstetrical and Neonatal Outcomes of Pregnancies?.Obes surg. 2019; 29: 1498–1505.	Not seeking IVF
Burnik Papler 2022	Burnik Papler T, Abdulkhalikova D, Jancar N, et al. Spontaneous pregnancy rates after the weight loss program in infertile PCOS women with obesity. Human Reproduction. 2022; 37 (Supplement 1): i460.	Published only as a conference abstract
Dwivedee 2010<br><br>Mohan 2012	Dwivedee K, Mohan S, Peters ANC. PCOS-A weighty problem. Does bariatric surgery have the answer? Gynecological Surgery 2010;7(SUPPL. 1):S77.<br><br>Mohan S, Dwivedee K, Peters ANC, et al. PCOS - A metabolic problem. Does bariatric surgery hold the key? Obes Surg 2012;22(9):1374.	Published only as a conference abstract
Muirhead 2021<br><br>Gordon 2020	Muirhead R, Kizirian N, Lal R, et al. A Pilot Randomized Controlled Trial of a Partial Meal Replacement Preconception Weight Loss Program for Women with Overweight and Obesity. Nutrients. 2021;13(9).<br><br>Gordon A, Muirhead R, Lal R, et al. Pre-babe: Impact of pre-conception weight loss for women above a healthy weight. J Paediatr Child Health. 2020;56(SUPPL 1):17.	Not seeking IVF
Fawcett 2021	Fawcett K, Martinez A, Crimmins M, et al. Effect of a dietary and exercise intervention in women with overweight and obesity undergoing fertility treatments: protocol for a randomized controlled trial. BMC Nutr. 2021;7(1):51.	No full publication. The trial has been terminated.
Mayor 2016	Mayor S. Weight loss does not improve birth rate in obese women with infertility, study shows. Bmj. 2016;353:i2939.	Commentary
Palomba 2008	Palomba S, Giallauria F, Falbo A, et al. Structured exercise training programme versus hypocaloric hyperproteic diet in obese polycystic ovary syndrome patients with anovulatory infertility: a 24-week pilot study. Hum Reprod. 2008;23(3):642-50.	Not seeking IVF
Chavarro 2012	Chavarro JE, Ehrlich S, Colaci DS, et al. Body mass index and short-term weight change in relation to treatment outcomes in women undergoing assisted reproduction. Fertil Steril. 2012; 98: 109–16.	Number of events for pregnancy and live birth outcomes not available specifically for those with obesity, only an association with BMI was given.
Ahmed 2017	Ahmed HO. Improvement in Fertility after Bariatric Surgery in Obese Females with Polycystic Ovarian Syndrome: Based on Four Years of Experience in Two Centers in Sulaimani Governorate, Kurdistan Region/Iraq. Bariatr Surg Pract Patient Care. 2017;12(4):162-169.	Not seeking IVF

Study ID	Identified record/reference	Reason for exclusion
Blanco-Breindel 2023	Blanco-Breindel M, Rezk A, Liu A, et al. OVULATION INDUCTION OUTCOMES IN INFERTILE OBESE WOMEN WITH A HISTORY OF BARIATRIC SURGERY. Fertil Steril. 2023;120(1 Supplement):e57-e58.	Not seeking IVF
Phelan 2013	Phelan S, Hagobian T, Brannen A. Promoting Weight Loss Before Pregnancy: Feasible or Futile? Calif J Health Promot. 2013;11(2):86-92.	Not seeking IVF
Dayan 2014	Dayan N, Pilote L, Opatrny L, et al. Assisted reproductive therapy in women with higher body mass index. J Obstet Gynaecol Can. 2014; 36: 513–4.	Wrong study design (commentary)
Steegers-Theunissen 2020	Steegers-Theunissen R, Hoek A, Groen H, et al. Pre-Conception Interventions for Subfertile Couples Undergoing Assisted Reproductive Technology Treatment: Modeling Analysis. JMIR Mhealth Uhealth. 2020; 8: e19570.	Wrong study design (modelling analysis of previous interventions)
	Losing weight may not increase chances of pregnancy, says new study. Indian Practitioner. 2022;75(3):18-18.	No abstract or full text could be found, only title.
El Sharkwy 2019	El Sharkwy I, Sharaf El-Din M. l-Carnitine plus metformin in clomiphene-resistant obese PCOS women, reproductive and metabolic effects: a randomized clinical trial. Gynecol Endocrinol. 2019;35(8):701-5.	Not seeking IVF
Sant'Anna 2022	Sant'Anna EM, Paiva SPC, Santos RP, et al. Mindfulness-based program to support lifestyle modification and weight loss in infertile women: randomized controlled trial. J Psychosom Obstet Gynaecol. 2022;43(2):136-44.	Not seeking IVF
Rothberg 2016	Rothberg A, Lanham M, Randolph J, et al. Feasibility of a brief, intensive weight loss intervention to improve reproductive outcomes in obese, subfertile women: a pilot study. Fertil Steril. 2016;106(5):1212-20.	Not seeking IVF
Chan 2022	Chan JKY, Ku CW, Loy SL, et al. Effects of an integrated mobile health lifestyle intervention among overweight and obese women planning for pregnancy in Singapore: protocol for the single-arm healthy early life moments in Singapore (HELMS) study. BMJ Open. 2022;12(12):e061556.	Ongoing study
Johnson 2006	Johnson NP. No more surrogate end-points in randomised trials: the PCOSMIC trial protocol for women with polycystic ovary syndrome using metformin for infertility with clomiphene. Aust N Z J Obstet Gynaecol. 2006;46(2):141-5.	Not seeking IVF
Duval 2015<br><br><br><br>Belan 2019<br><br><br><br>Belan 2019<br><br><br><br>Belan 2021<br><br><br><br>Rouissi 2020<br><br><br><br>Rouissi 2020	Duval K, Langlois MF, Carranza-Mamane B, et al. The Obesity-Fertility Protocol: a randomized controlled trial assessing clinical outcomes and costs of a transferable interdisciplinary lifestyle intervention, before and during pregnancy, in obese infertile women. BMC Obesity. 2015; 2: 47.<br><br>Belan M, Carranza-Mamane B, Ainmelk Y, et al. A Lifestyle Program Targeting Women with Obesity and Infertility Improves Their Fertility: A Randomized Controlled Trial. JES. 2019; 3(Supplement 1).<br><br>Belan M, Carranza-Mamane B, Melk YA, et al. Lifestyle intervention targeting women with obesity and infertility improves their fertility outcomes, especially in women with PCOS: a randomized controlled trial. Fertil Steril. 2019; 112(3 SUPPL): e40.<br><br>Belan M, Carranza-Mamane B, Melk YA, et al. Cost-Effectiveness Analysis of an Interdisciplinary Lifestyle Intervention Targeting Women With Obesity and Infertility in Comparison to Usual Care. JES. 2021; 5(Supplement 1): A730.<br><br>Rouissi M, Levesque MA, Hebert MC, et al. A Preconception Lifestyle Intervention Maintained Throughout Pregnancy Improves Some Gestational and Neonatal Outcomes in Women With Obesity and Infertility. JES. 2020; 4(Supplement 1): A1134.<br><br>Rouissi M, Jean-Denis F, Belan M, et al. A preconception lifestyle intervention improves some gestational outcomes and neonatal markers of adiposity in women with obesity and infertility. Fertil Steril. 2020; 114(3 SUPPL): e466–e467.	Published only as a conference abstract

Study ID	Identified record/reference	Reason for exclusion
Belan 2022	Belan M, Gélinas M, Carranza-Mamane B, et al. Protocol of the Fit-For-Fertility study: a multicentre randomised controlled trial assessing a lifestyle programme targeting women with obesity and infertility. BMJ Open. 2022; 12: e061554.	Ongoing study
Filippone 2024	Filippone M, Lafleche CRD, Belan M, et al. Subgroup Analyses of a Randomized Controlled Trial Evaluating the Effects of a Lifestyle Intervention on Fertility Outcomes in Women With Obesity and Infertility. Canadian J Diabetes 2024;48:S38-S38.	Published only as a conference abstract (probably related to either the study as per Duval et al 2015 protocol above, and/or the study by Belan et al 2022 above)
Palomba 2014	Palomba S, Falbo A, Valli B, et al. Physical activity before IVF and ICSI cycles in infertile obese women: an observational cohort study. Reprod Biomed Online. 2014; 29:72–9.	No weight loss intervention
Kawabata 2023	Kawabata N, Ishida Y, Nagamine C, et al. Clinical, Physical, and Nutritional Effects of Weight Loss Program for Obese and Infertile patients. Ann Nutr Metab. 2023; 79(Supplement 1): 644	Published only as a conference abstract
Palomba 2010	Palomba S, Falbo A, Giallauria F, et al. Six weeks of structured exercise training and hypocaloric diet increases the probability of ovulation after clomiphene citrate in overweight and obese patients with polycystic ovary syndrome: a randomized controlled trial. Hum Reprod. 2010;25(11):2783-91.	Not seeking IVF
Liu 2024	Liu J, Gu J, Zhang J, Xing D, et al. Improvement of Polycystic Ovary Syndrome Symptoms in Obese Patients. Altern Ther Health Med. 2024.	Not seeking IVF
Van Veen 2011	Van Veen LJ, Van Den Dool GC, Rijnsaardt HGM, et al. The development of a life style program aimed at weight reduction in obese patients with subfertility in a large district hospital, results after five years. Hum Reprod. 2011; 26(SUPPL. 1): i57	Published only as a conference abstract
Misharina 2019	Misharina EV, Borovik NV. Retrospective analysis of pregnancies resulted from assisted reproductive technology in women with type 2 diabetes mellitus. Journal of Obstetrics and Women's Diseases. 2019; 68: 25–34	Already included women who were pregnant and with a child and then looked at other outcomes according to who received or not the weight loss intervention. Therefore, by default pregnancy and live births are not an outcome.
Dattilo 2023	Kucuk T, Horozal PE, Karakulak A, et al. Follicular homocysteine as a marker of oocyte quality in PCOS and the role of micronutrients. J Assist Reprod Genet. 2023;40(8):1933-41.	No weight loss intervention
Legro 2022<br><br>Legro 2021<br><br>Legro 2020	Legro RS, Hansen KR, Diamond MP, et al. Effects of preconception lifestyle intervention in infertile women with obesity: The FIT-PLESE randomized controlled trial. PLoS Med. 2022; 19: e1003883.<br><br>Vitek W, Sun F, Cardozo E, et al. Increased physical activity is not detrimental to live birth in women with obesity and infertility. Fertil Steril. 2021; 116(3 SUPPL): e52–e53.<br><br>Legro RS, Hansen KR, Diamond MP, et al. Effect of Preconception Intensive vs. Standard Lifestyle Intervention on Birth Outcomes in Obese Women With Unexplained Infertility: A Multicenter Randomized Trial. JES. 2020; 4(Supplement 1): A1084.	Not seeking IVF
Akbari 2010	Akbari Asbagh F, Papan M, Khazaeipour Z. The effect of metformin treatment on ICSI in infertile polycystic ovary syndrome. Tehran Univ Med J. 2010; 67: 849–55.	Metformin is not an approved weight loss drug and authors do not report weight outcomes. As a result, it is not clear if this can be considered a weight loss intervention.
Tsagareli 2006	Tsagareli V, Noakes M, Norman RJ. Effect of a very-low-calorie diet on in vitro fertilization outcomes. Fertil Steril. 2006; 86: 227–9.	No pregnancy outcomes reported
Timmermans 2019	Timmermans YEG, van de Kant KDG, Reijnders D, et al. Towards Prepared mums (TOP-mums) for a healthy start, a lifestyle intervention for women with overweight and a child wish: study protocol for a randomised controlled trial in the Netherlands. BMJ Open. 2019; 9: e030236.	Ongoing study
Malhotra 2023	Malhotra N, Arora T, Suri V, et al. Individualized lifestyle intervention in PCOS women (IPOS): a study protocol for a multicentric randomized controlled trial for evaluating the effectiveness of an individualized lifestyle intervention in PCOS women who wish to conceive. Trials. 2023;24(1):457.	Not seeking IVF
Sauder 2023	Sauder KA, Gamalski K, DeRoeck J, et al. A pre-conception clinical trial to reduce intergenerational obesity and diabetes risks: The NDPP-NextGen trial protocol. Contemp Clin Trials. 2023;133:107305.	Not seeking IVF

Study ID	Identified record/reference	Reason for exclusion
Al Kuwari 2015	Al Kuwari M, Jabbour G, El Batbouly M. The potential role of bariatric surgery in the treatment of obesity related female infertility: Our experience in Qatar. Obes Surg 2015;25(1 SUPPL. 1):S250-S251.	Published only as a conference abstract
Meneghini 2023<br><br>Meneghini 2024	Meneghini C, Bianco C, Galanti F, et al. The Impact of Nutritional Therapy in the Management of Overweight/Obese PCOS Patient Candidates for IVF. Nutrients. 2023; 15: 4444.<br><br>Meneghini C, Bianco C, Galanti F, et al. Reply to Cetkovic et al. Comment on "Meneghini et al. The Impact of Nutritional Therapy in the Management of Overweight/Obese PCOS Patient Candidates for IVF. Nutrients 2023, 15, 4444". Nutrients. 2024;16(3):439.	Did not report pregnancy or live birth outcomes.
Bazarah 2021	Bazarah MS, Badeghiesh A, Baghlaf H, et al. The effect of bariatric surgery on obese polycystic ovarian syndrome (PCOS) patients' obstetrical and neonatal outcomes: A population based study. Fertil Steril. 2021; 116(3 SUPPL): e119.	Not seeking IVF
Grzegorczyk-Martin 2017<br><br>Grzegorczyk-Martin 2018<br><br>Grzegorczyk-Martin 2020	Grzegorczyk Martin V, Finet A, Landais P, et al. Impact of dramatic weight loss linked to bariatric surgery on ovarian response and pregnancy rates after in vitro fertilization (IVF): A case-control study. Hum Reprod. 2017; 32(Supplement 1): i29–i30.<br><br>Grzegorczyk Martin V, Finet De Bantel A, Bonnet E, et al. In vitro fertilization (IVF) outcomes after dramatic weight, loss linked to bariatric surgery: A case-control study. Hum Reprod. 2018; 33(Supplement 1): i311–i312.<br><br>Grzegorczyk-Martin V, Fréour T, De Bantel Finet A, et al. IVF outcomes in patients with a history of bariatric surgery: a multicenter retrospective cohort study. Hum Reprod. 2020; 35: 2755–62.	Non-RCT which does not report weight outcomes, and all other associated records are published only as conference abstracts.
Weisman 2011	Weisman CS, Hillemeier MM, Downs DS, et al. Improving women's preconceptional health: long-term effects of the Strong Healthy Women behavior change intervention in the central Pennsylvania Women's Health Study. WHI. 2011; **21:** 265–71.	Not seeking IVF
Karlsen 2013	Karlsen K, Humaidan P, Sørensen LH, et al. Motivational interviewing: a part of the weight loss program for overweight and obese women prior to fertility treatment. Gynecol Endocrinol 2013; 29: 839–42.	Did not report pregnancy or live birth outcomes
Clark 1995	Clark AM, Ledger W, Galletly C, et al. Weight loss results in significant improvement in pregnancy and ovulation rates in anovulatory obese women. Hum Reprod. 1995; 10: 2705–12.	Data not available specifically for those seeking IVF
Clark 1998	Clark AM, Thornley B, Tomlinson L, Galletley C, et al. Weight loss in obese infertile women results in improvement in reproductive outcome for all forms of fertility treatment. Hum Reprod. 1998; 13: 1502–5.	Data not available specifically for those seeking IVF
Qublan 2007	Qublan HS, Yannakoula EK, Al-Qudad M, et al. Dietary intervention versus metformin to improve the reproductive outcome in women with polycystic ovary syndrome. A prospective comparative study. Saudi Med J. 2007;28(11):1694-1698.	Not seeking IVF
Galletly 1996	Galletly C, Clark A, Tomlinson L. Evaluation of dexfenfluramine in a weight loss program for obese infertile women. Int J Eat Disord. 1996;19(2):209-12.	Crossover trial
Khazraei 2017	Khazraei H, Hosseini SV, Amini, M, et al. Effect of weight loss after laparoscopic sleeve gastrectomy on infertility of women in shiraz. J Gynecol Surg. 2017;33(2):43-46.	Not seeking IVF
Eden 2003	Eden B. [Overweight and obesity reduce fertility of women. Weight reduction and physical exercise increase the chance to become pregnant]. Lakartidningen. 2003;100(49):4096-9.	Commentary
Zhang 2017	Zhang J, Si Q, Li J. Therapeutic effects of metformin and clomiphene in combination with lifestyle intervention on infertility in women with obese polycystic ovary syndrome. Pak J Med Sci. 2017;33(1):8-12.	Not seeking IVF
Legro 2012	Legro RS, Dodson WC, Gnatuk CL, et al. Effects of gastric bypass surgery on female reproductive function. J Clin Endocrinol Metab. 2012;97(12):4540-8.	Not seeking IVF

Study ID	Identified record/reference	Reason for exclusion
Ahuja 2019	Ahuja A. Role of bariatric surgery in Polycystic Ovarian Syndrome and Infertility. Obes Surg. 2019;29(Supplement 1):S27.	Not seeking IVF
Bivia-Roig 2020	Biviá-Roig G, Blasco-Sanz R, Boldó-Roda A, et al. Efficacy of an Internet-Based Intervention to Promote a Healthy Lifestyle on the Reproductive Parameters of Overweight and Obese Women: Study Protocol for a Randomised Controlled Trial. Int J Environ Res Public Health. 2020;17(22).	No results published, only protocol, though not ongoing study.
Stamets 2004	Stamets K, Taylor DS, Kunselman A, et al. A randomized trial of the effects of two types of short-term hypocaloric diets on weight loss in women with polycystic ovary syndrome. Fertil Steril. 2004;81(3):630-7.	Not seeking IVF
Boedt 2023	Boedt T, Dancet E, De Neubourg D, et al. A blended preconception lifestyle programme for couples undergoing IVF: lessons learned from a multicentre randomized controlled trial. Hum Reprod Open. 2023;2023(4):hoad036.	Average BMI <27 kg/m2
Najmabadi 2023	Najmabadi KM, Amiri B, Asgharipour N, et al. The Effect of Cognitive-Behavioral Counseling on Pre-conception Physical Activity in Women with High Body Mass Index: A Randomized Controlled Trial. JMRH. 2023;11(3):3822-3831.	Not seeking IVF
Hollmann 1996	Hollmann M, Runnebaum B, Gerhard I. Effects of weight loss on the hormonal profile in obese, infertile women. Hum Reprod. 1996;11(9):1884-91.	Not seeking IVF
An 2014	An Y, Sun Z, Zhang Y, Liu B, et al. The use of berberine for women with polycystic ovary syndrome undergoing IVF treatment. Clin Endocrinol (Oxf). 2014;80(3):425-31.	Berberine and metformin are not approved weight loss drugs.
Nilsson-Condori 2022	Nilsson-Condori E, Mattsson K, Thurin-Kjellberg A, et al. Outcomes of in-vitro fertilization after bariatric surgery: a national register-based case–control study. Hum Reprod. 2022; 37: 2474–81.	Non-RCT which does not report weight outcomes
Tsur 2014	Tsur A, Orvieto R, Haas J, et al. Does bariatric surgery improve ovarian stimulation characteristics, oocyte yield, or embryo quality? J Ovarian Res. 2014; 7: 116.	Did not report pregnancy or live birth outcomes
Parent 2016	Parent C, Pigeyre M, Pleuvret A, et al. Infertilité : moment clé pour entreprendre une prise en charge nutritionnelle médicale. Expérience lilloise sur 78 patientes. Gynecol Obstet Fertil. 2016; 44: 218–24.	It is unclear if the BMI data reported specifically for women with excess weight seeking IVF in Table 6 are from baseline or follow-up, and since this is a non-RCT, we cannot include this study in the meta-regression. BMI data at follow-up seem to be reported only for the whole sample (Table 2).
Nair 2020	Nair S, Tamboli R, Marks PA, et al. Roux-en-Y gastric bypass induced weight loss improves reproductive hormone profile in women. Reprod Sci. 2010;17(3 SUPPL. 1):199A-200A.	Published only as a conference abstract
De Cos 2020	De Cos A, Calvo I, De Leon B, et al. Weight control and obstetric outcomes in assisted reproduction. Obes Rev. 2020;21(SUPPL 1):	Published only as a conference abstract
Maloy 2025	Maloy K. Increasing Fertility and Live Birth Babies for Overweight Women with Polycystic Ovary Syndrome: Interventions and Treatments. Nutritional Perspectives: Journal of the Council on Nutrition. 2025;48:15-28.	Review of evidence
Incognito 2024	Incognito GG, Vaiarelli A, Fabozzi G, et al. Effect of weight loss interventions on fertility in overweight or obese women: is it time to change the approach? J Obstet Gynaecol. 2024;44:2420163	Review of evidence
Shan 2024	Shan Y, Han X, Yang C, et al. The impact of metabolic surgery on natural conception rates in women with infertility, obesity and polycystic ovary syndrome: a retrospective study. Surg Obes Relat Dis. 2024;20:237-243.	Suggestive that not everyone was seeking IVF

IVF: in vitro fertilization; RCT: randomized controlled trial; BMI: body mass index

Table S3. Detailed characteristics of included randomized controlled trials

Study & Country	Participants						Interventions								Outcome
	**N randomized**	**Age** mean (SD)	**BMI** mean (SD)	**Ethnicity** % White*	**Infertility** Duration mean (SD) and Cause %*	**Conditions** %*	**Content**	**Provider**	**Mode**	**Level of personal contact**	**Program duration** weeks, unless otherwise specified	**Sessions / Dosage**			**Weight change (kg)**
												**n or amount**	**Frequency**	**Length per session**	
**Diet and/or physical activity vs no/minimal intervention**															
**Moran 2011**<br><br>Australia	46	33.1 (3.4)	34.0 (4.4)	NR	Duration: 4.7 (2.4) years<br><br>Causes:<br>• Female: 26.3<br>• Male: 44.7<br>• Combined: 15.8<br>•Unexplained: 5.3	PCOS: 0	**Intervention:**<br>• High-protein weight loss diet: 5368 kJ/day , 99.7 g protein/day, 35.9 g fat/day, 10.6 g saturated fat/day, 128.8 g carbohydrate/day, 23.2 g fiber/day<br>• Replacement of one daily meal with liquid formula and 200 ml reduced-fat milk<br>• Exercise: home-based physical conditioning and walking program	Researchers	Individual	Face-to-face and telephone	5-9	3 in total	At baseline, 2 weeks, and 4 weeks	NR	-3.8
							**Comparator:**<br>One-off standard advice on diet and lifestyle factors influencing fertility, with no active follow-up	Researchers	Individual	Face-to-face	One-off	1	One-off	NR	-0.5
**Becker 2015**<br><br>Brazil	35	31.3 (3.0)	28.7 (2.8)	NR	Duration: NR<br><br>Causes:<br>• Tubal factor: 50<br>• Tubal factors + PCOS: 15.4<br>•Endometriosis: 19.2<br>• Unexplained: 15.4	PCOS: at least 15.4<br><br>Endometriosis: at least 19.2	**Intervention:**<br>• Hypocaloric, low glycaemic index and low glycaemic load diet: 20 kcal/kg weight, 50% energy from carbohydrate, 20% energy from protein, 30% energy from fat, with high intake of vegetables and salads, and	Dietitians	Individual	Face-to-face	12	3 in total	At weeks 0, 6 and 12	NR	-4.51

							discouragement of added sugars and alcohol<br>• Forms and instructions, including description of food portions<br>• Portfolio with photos on the basis of the Photo-Registry for Dietary Surveys<br>• Supply of olive oil and dried fruit<br>• Recommendation to not start any physical exercise throughout the study period								
							**Comparator:**<br>• Continue with their usual diet<br>• Recommendation to not start any physical exercise throughout the study period	NA	NA	NA	12	NA	NA	NA	0.72
**Espinos 2017**<br><br>Spain	41	32.4 (3.5)	34.3 (3.6)	NR	Duration:<br>5.0 (2.9) years<br><br>Causes:<br>• Tubal factor: 24.4<br>• Male factor: 63.4<br>• Ovulatory: 51.2<br>•Unexplained: 12.2	PCOS: 41.5	**Intervention:**<br>• Standardized weight loss program: calorie deficit of 500-800kcal/day, 50% of energy intake from carbohydrate, <10% of energy intake from saturated fat, 20% of energy intake from mono- or polyunsaturated fat, <300mg cholesterol/day, 1g protein/kg ideal body weight, and at least 15g of fibre per 1000kcal<br>• Exercise program: walking on a treadmill or pedalling 60 min three times per week	Diet:<br>Dietitians<br><br>Exercise:<br>trained staff	Individual	Diet:<br>unclear<br><br>Exercise:<br>face-to-face	12	Diet:<br>NR<br><br>Exercise:<br>36 in total	Diet:<br>NR<br><br>Exercise:<br>3 times/week	Diet:<br>NR<br><br>Exercise:<br>60 min	-6.4
							**Comparator**<br>Immediate IVF	NA	NA	NA	NA	NA	NA	NA	NR

**Mutsaerts 2016**<br><br>Nether-lands	137	NR for those seeking IVF only	35.5 (4.9)	NR for those seeking IVF only	Duration:<br>NR for those seeking IVF only<br><br>Causes:<br>NR for those seeking IVF only	NR for those seeking IVF only	**Intervention:**<br>• Caloric reduction of 600 kcal/day, but total caloric intake no less than 1200 kcal/day<br>• Web-based food diary<br>• Moderate-intensity exercise and aim for 10,000 steps/day<br>• Pedometer<br>• Physical activity diary<br>• Individualized motivational counseling	Intervention coaches with a degree in nursing or dietitians	Individual	Face-to-face plus telephone or email	24	10	NR	Visits:<br>1st visit, 45-60 min<br>2nd visit, 45-60 min<br>3rd visit, 30 min<br>4th visit, 30 min<br>5th visit, 30 min<br>6th visit, 30 min<br>All phone calls 15 min	-4.6
							**Comparator:**<br>Immediate IVF	NA	NA	NA	NA	NA	NA	NA	-0.73
**Wang 2023**[‡]<br><br>China	38 met eligibility criteria	30.0 (3.2)	27.8 (2.4)	NR	NR	PCOS: 0	**Intervention:**<br>• Caloric reduction by 500 kcal/day with the help of a diary, while maintaining a minimum caloric intake of 1200 kcal/day<br>• Engage in moderate-intensity physical activity with a target level of 10,000 steps/day and at least 30 min of moderate-intensity exercise two or three times per week<br>• Encouraged to use application to record daily diet, exercise, and weight in different ways, such as text, voice, picture, and video for self-monitoring, while at the same time being followed-up and guided regularly by dietitians via the app	Dietitians	Individual	Suggestive of face-to-face plus via the app	12	NR but could get follow-up and guidance daily via the app	NR but could get follow-up and guidance daily via the app	NR	-3.7
							**Comparator:**<br>No intervention	NA	NA	NA	NA	NA	NA	NA	-0.5

**Diet and/or physical activity vs active comparator**															
**Kiel 2018**<br><br>Norway	18	32.3 (5.0)	30.2 (2.4)	NR	Duration:<br>NR<br><br>Causes:<br>NR	NR	**Intervention:**<br>• High-intensity interval training<br>• Free membership at the local gym<br>• Heart rate monitors<br>• Training diary<br>• Encouraged to adhere to the current Norwegian diet recommendations	NR	Individual	Face-to-face	13-15	39-45 in total	3 times per week	NR	-0.6
							**Comparator:**<br>• Standard care: regular advice about physical activity, and not discouraged from being physically active<br>• Encouraged to adhere to the current Norwegian diet recommendations	NR	Individual	NR	NR	NR	NR	NR	-0.7
**Low-energy diet with/without physical activity advice vs no/minimal intervention**															
**Einarsson 2017**<br><br>Sweden	317	31.6 (4.2)	33.1 (1.4)	88.3	Duration:<br>38.7 (22.9) months<br><br>Causes:<br>• Tubal factor: 8.5<br>• Male factor: 30.3<br>• PCOS: 19.9<br>•Endometriosis: 2.5<br>• Unexplained: 28.7<br>• Other: 6.3	PCOS:<br>at least 19.9<br><br>Endometriosis:<br>at least 2.5	**Intervention:**<br>• Low calorie liquid formula diet with a daily energy intake of 880 kcal, with re-introduction of solid foods, and weight stabilization before IVF<br>• During and after IVF, also complementary dietary counseling	Dietitians	Individual	Face-to-face	Weight loss phase:<br>12<br><br>Weight control phase:<br>2-5<br><br>During and after IVF: complementary dietary counseling for one year from randomisation	Weight loss phase:<br>5<br><br>Weight control phase:<br>NR<br><br>Prior to IVF treatment, the patient met the dietitian again for a follow-up visit.<br><br>During and after IVF:<br>NR	Weight loss phase: at weeks 0, 2, 5, 8 and 12<br><br>Weight control phase:<br>NR<br><br>Prior to IVF treatment, the patient met the dietitian again for a follow-up visit.<br><br>During and after IVF:<br>NR	NR	-9.1
							**Comparator:**<br>Immediate IVF	NA	NA	NA	NA	NA	NA	NA	-1.19

**Low-energy diet with/without physical activity advice vs active comparator**														
**Sim 2014**<br><br>Australia	49	32.9 (3.2)	36.4 (4.7)	3.6 (1.6)	Duration: NR<br><br>Causes:<br>• Tubal: 16.3<br>• Endometriosis: 18.4<br>• PCOS: 30.6<br>• Male factor: 49.0<br>• Ovulation disorder: 28.6<br>•Unexplained: 6.1	PCOS: at least 30.6<br><br>Endometriosis: at least 18.4	**Intervention:**<br>• Very-low-energy diet, followed by a refeeding protocol leading to mild hypocaloric diet (2500 kJ/day deficit)<br>• A dietary modification tool and printed material<br>• Weekly feedback and encouragement<br>• Increase physical activity ultimately to a target of 10,000 daily steps<br>• Psychological support	Multi-disciplinary therapeutic team: fertility fellow, midwife, fertility counsellor, dietitian	Group	Face-to-face	12	12 in total	Once a week	NR	-6.6
							**Comparator:**<br>• GP-led weight loss advice or referral to public weight loss service if BMI>35 (responsibility for weight loss was placed upon the individual participant)<br>• Same printed material as the intervention group	GP for GP-led weight loss advice; Unclear who delivered the public weight loss service	Likely individual if GP-led weight loss; NR for public weight loss service	NR	NR	NR	NR	NR	-1.6
**Price 2020, 2021**<br><br>Australia	164	32.3 (4.5)	38.7 (1.1)	81.7	Duration: NR<br><br>Causes: NR	PCOS: 39	**Intervention:**<br>• Phase 1: Very-low-energy diet consisting of 2 daily formulas, plus a third meal consisting of 2 cups of low-starch vegetables, 150g lean meat, and 2 teaspoons of oil<br>• Phase 2: caloric intake approximately equal to energy expenditure, without specific macronutrient prescription, to stabilize weight	Dietitian	Individual	Face-to-face and telephone	Phase 1: 12<br><br>Phase 2: 3<br><br>Phase 3: 43	Phase 1: 2 daily formulas<br><br>Phase 2: daily weight maintenance diet<br><br>Phase 3: at least 3	Phase 1: daily formulas<br><br>Phase 2: Daily weight maintenance diet<br><br>Phase 3: study site/phone visits every 3 months	NR	-13.0

							• Phase 3: continue with weight maintenance diet<br>• Physical activity: aim for >10,000 steps/day, throughout						Physical activity: daily		
							**Comparator:**<br>• Phase 1: Standard dietary intervention: 500 kcal less than current daily intake, and with macronutrient composition consistent with national guidelines<br>• Phase 2: caloric intake approximately equal to energy expenditure, without specific macronutrient prescription, to stabilize weight<br>• Phase 3: continue with weight maintenance diet<br>• Physical activity: aim for >10,000 steps/day, throughout	Dietitian	Individual	Face-to-face and telephone	Phase 1: 12<br><br>Phase 2: 3<br><br>Phase 3: 43	Phase 1: daily low-calorie diet<br><br>Phase 2: daily weight maintenance diet<br><br>Phase 3: at least 3<br><br>Physical activity: daily	Phase 1: daily formulas<br><br>Phase 2: daily weight maintenance diet<br><br>Phase 3: study site/phone visits every 3 months<br><br>Physical activity: daily	NR	-3.2
**Pharmacotherapy with some diet and physical activity advice vs active comparator**															
**Salamun 2018**<br><br>Slovenia	27	30.6 (4.2)	36.6 (4.2)	NR	Duration: 3.7 (2.4) years<br><br>Causes: Primary infertility: 59.2	PCOS: 100	**Intervention:**<br>• Liraglutide plus metformin<br>• Active lifestyle intervention: caloric reduction by 500-800 kcal/day, with up to 50% energy intake from low-glycaemic carbohydrates, 20% energy intake from protein, 30% energy intake from fat, and <10% energy intake from saturated fat, and increase in fibers	Medication: clinician<br><br>Lifestyle advice: NR	Individual	NR	12	Medication: metformin 1000 mg plus liraglutide 1.2 mg<br><br>Lifestyle advice: NR	Metformin twice daily, plus liraglutide once daily	Medication: NA<br><br>Lifestyle advice: NR	-7.51

							• Physical activity: at least 30 min moderate/day								
							**Comparator:**<br>• Metformin<br>• Active lifestyle intervention: caloric reduction by 500-800 kcal/day, with up to 50% energy intake from low-glycaemic carbohydrates,<br>20% energy intake from protein, 30% energy intake from fat, and <10% energy intake from saturated fat, and increase in fibers<br>• Physical activity: at least 30 min moderate/day	Medication: clinician<br><br>Lifestyle advice:<br>NR	Individual	NR	12	Medication Metformin 1000 mg<br><br>Lifestyle advice:<br>NR	Metformin twice daily	Medication NA<br><br>Lifestyle advice:<br>NR	-6.99
**Wang 2021**<br><br>China	888	30.7 (4.0)	29.4 (3.1)	NR	Duration:<br>3.5 (2-5) years in intervention, and 3.0 (2-5) years in control<br><br>Causes:<br>• Pelvic factor: 48.3<br>• Male factor: 14.5<br>• Ovulatory dysfunction: 10.0<br>• Combined factors: 20.5<br>•Unexplained: 5.5<br>• Other: 1.1	NR	**Intervention:**<br>• Orlistat<br>• Verbal and written advice on lifestyle modifications aimed at a low-fat diet and high-quality physical activity<br>• Multivitamin supplement	Clinician	Individual	Face-to-face plus written materials	4-12 prior to ovarian hyper-stimulation	Orlistat: 120mg<br><br>Lifestyle advice:<br>NR	Orlistat: thrice daily<br><br>Lifestyle advice:<br>NR	Orlistat: NA<br><br>Lifestyle advice:<br>NR	-2.49
							**Comparator:**<br>• Placebo<br>• Verbal and written advice on lifestyle modifications aimed at a low-fat diet and high-quality physical activity<br>• Multivitamin supplement	Clinician	Individual	Face-to-face plus written materials	4-12 prior to ovarian hyper-stimulation	Placebo: 120mg<br><br>Lifestyle advice:<br>NR	Placebo: thrice daily<br><br>Lifestyle advice:<br>NR	Placebo: NA<br><br>Lifestyle advice:<br>NR	-1.22

**Li 2022**<br><br>China	160	28.0 (3.7)	29.1 (4.0)	0	Duration: more than 2 years<br><br>Causes: NR	PCOS: 100	**Intervention:**<br>• Exenatide and then switch to metformin<br>• Lifestyle advice according to national guidance	Medication: clinician<br><br>Lifestyle advice: NR	Individual	Face-to-face	Exenatide for 12 weeks, and then switch to metformin for another 12, therefore total of 24	Exenatide: 5 mcg initially, and then 10 mcg after 4 weeks<br><br>Metformin: 500 mg and titrated by 500 mg every 3 days up to 1000 mg<br><br>Lifestyle advice: NR	Exenatide: twice daily<br><br>Metformin: twice daily<br><br>Lifestyle advice: NR	Exenatide: NA<br><br>Metformin: NA<br><br>Lifestyle advice: NR	-5.21
							**Comparator:**<br>• Metformin<br>• Lifestyle advice according to national guidance	Medication: clinician<br><br>Lifestyle advice: NR	Individual	Face-to-face	24	Metformin: for 12 weeks, 500 mg, and titrated by 500 mg every 3 days up to 1000 mg, and for another 12 weeks, continue with 1000 mg<br><br>Lifestyle advice: NR	Metformin: twice daily<br><br>Lifestyle advice: NR	Metformin: NA<br><br>Lifestyle advice: NR	-3.55

SD: standard deviation; BMI: body mass index; NR: not reported; PCOS: polycystic ovary syndrome; NA: not applicable; IVF: in vitro fertilisation; OGTT: oral glucose tolerance test

*Denominator is the number of participants for whom authors reported data at baseline.

‡ Wang 2023 included one more potentially eligible comparison: hypocaloric diet + physical activity + metformin vs metformin only, however, we chose to include only the most straightforward comparison (hypocaloric diet + physical activity vs no intervention) in this review. This was decided in order to avoid using the Wang 2023 as two separate studies into the meta-analyses, which could potentially affect confidence intervals due to data and estimates from the two separate entries not being independent

Table S4. Detailed risk of bias assessment of included individual randomized controlled trials

**Becker 2015**		
**Domain 1**		
**1.1 Was the allocation sequence random?**	**1.2 Was the allocation sequence concealed until participants were enrolled and assigned to interventions?**	**1.3 Did baseline differences between intervention groups suggest a problem with the randomization process?**
**NI:** "The study was a randomized controlled trial that followed a randomized block design." No further information given.	**NI**: "The study was a randomized controlled trial that followed a randomized block design." No other further information given.	**N**: No differences in baseline characteristics from Table 2.

**Domain 2**						
**2.1. Were participants aware of their assigned intervention during the trial?**	**2.2. Were carers and people delivering the interventions aware of participants' assigned intervention during the trial?**	**2.3. If Y/PY/NI to 2.1 or 2.2: Were there deviations from the intended intervention that arose because of the trial context?**	**2.4 If Y/PY to 2.3: Were these deviations likely to have affected the outcome?**	**2.5. If Y/PY/NI to 2.4: Were these deviations from intended intervention balanced between groups?**	**2.6 Was an appropriate analysis used to estimate the effect of assignment to intervention?**	**2.7 If N/PN/NI to 2.6: Was there potential for a substantial impact (on the result) of the failure to analyse participants in the group to which they were randomized?**
**Y:** "It was not possible to blind the dietitians who advised on dietary changes and the patients to the treatment allocation because only one of the 2 groups of the study presented modifications in their usual diets."	**Y:** "It was not possible to blind the dietitians who advised on dietary changes and the patients to the treatment allocation because only one of the 2 groups of the study presented modifications in their usual diets."	**PN:** No information given, but there is no evidence of deviation due to the trial context, apart from 1 person who could not comply with the diet. Also, they do describe methods of assessing fidelity.	**N/A**	**N/A**	**N:** ITT analysis. However, they only report spontaneous pregnancies, not the success rate in those who received IVF. They also mention that that these spontaneous pregnancies led to live birth. But, in their trial registration page they mention that they meant to follow-up women up to 18 weeks post IVF and report they clinical pregnancy rates, but they haven't.	**Y:** Although they used ITT analysis, failing to analyse clinical pregnancies after IVF completion, severly impacts the result of the number of clinical pregnancies and live births achieved.

**Domain 3**			
**3.1 Were data for this outcome available for all, or nearly all, participants randomized?**	**3.2 If N/PN/NI to 3.1: Is there evidence that the result was not biased by missing outcome data?**	**3.3 If N/PN to 3.2: Could missingness in the outcome depend on its true value?**	**3.4 If Y/PY/NI to 3.3: Is it likely that missingness in the outcome depended on its true value?**
**N:** Only spontaneous pregnancies reported, not the success rate in those who received IVF. They also mention that that these spontaneous pregnancies led to live birth. But, in their trial registration page they mention	**N:** Only spontaneous pregnancies reported, not the success rate in those who received IVF. They also mention that that these spontaneous pregnancies led to live birth. But, in their trial registration page they mention that they meant	**NI:** No rationale or information given for why they did not report clinical pregnancy and live birth outcomes for all participants.	**NI:** No rational or information given for why they did not report clinical pregnancy and live birth outcomes for all participants.

that they meant to follow-up women up to 18 weeks post IVF and report they clinical pregnancy rates, but they haven't. Therefore, we only have clinical pregnancy and live birth data only for 3 of the 35 participants.	to follow-up women up to 18 weeks post IVF and report they clinical pregnancy rates, but they haven't. Therefore, we only have clinical pregnancy and live birth data only for 3 of the 35 participants.		

Domain 4

**4.1 Was the method of measuring the outcome inappropriate?**	**4.2 Could measurement or ascertainment of the outcome have differed between intervention groups?**	**4.3 If N/PN/NI to 4.1 and 4.2: Were outcome assessors aware of the intervention received by study participants?**	**4.4 If Y/PY/NI to 4.3: Could assessment of the outcome have been influenced by knowledge of intervention received?**	**4.5 If Y/PY/NI to 4.4: Is it likely that assessment of the outcome was influenced by knowledge of intervention received?**
**N:** Live births are objective. They do not specify in the paper how they assessed clinical pregnancies, however, in the trial registration page they mention that they used ultrasound to detect gestational sac or clinical signs of pregnancy.	**PN:** Method of assessment is the same in both groups, but timeline of follow-up for pregnancies/live births is unclear. However, both groups seem to have started IVF at the same time, and in trial registration page, they mention that clinical pregnancies would be assessed up to 18 weeks from baseline in average, two weeks after the end of the in vitro fertilization cycle.	**Y:** "It was not possible to blind the dietitians who advised on dietary changes and the patients to the treatment allocation because only one of the 2 groups of the study presented modifications in their usual diets."	**NI:** No rational or information given for why they did not report clinical pregnancy and live birth outcomes for all participants.	**NI:** No rational or information given for why they did not report clinical pregnancy and live birth outcomes for all participants.

Domain 5

**5.1 Were the data that produced this result analysed in accordance with a pre-specified analysis plan that was finalized before unblinded outcome data were available for analysis?**	**5.2 Is the numerical result being assessed likely to have been selected, on the basis of the results, from...multiple eligible outcome measurements (e.g. scales, definitions, time points) within the outcome domain?**	**5.3 Is the numerical result being assessed likely to have been selected, on the basis of the results, from...multiple eligible analyses of the data?**
**N:** In their trial registration page, they mention they would look at number of clinical pregnancies up to 18 weeks from baseline - two weeks after the end of IVF cycle, but they haven't, they only provide data on spontaneous pregnancies.	**N:** In their trial registration page, they mention they would look at number of clinical pregnancies up to 18 weeks from baseline - two weeks after the end of IVF cycle, but they haven't, they only provide data on spontaneous pregnancies.	**N:** There is only one possible way in which the outcome measurement can be analysed.

Einarsson 2017

Domain 1

**1.1 Was the allocation sequence random?**	**1.2 Was the allocation sequence concealed until participants were enrolled and assigned to interventions?**	**1.3 Did baseline differences between intervention groups suggest a problem with the randomization process?**
**Y:** "Randomization was performed with a computerized randomization programme with concealed allocation of patients and in the proportion of 1:1."	**Y:** "Randomization was performed with a computerized randomization programme with concealed allocation of patients and in the proportion of 1:1."	**N**: "Baseline characteristics were similar in the two groups, except that more terminations of pregnancies had occurred in the control group" (see Table 1)

Domain 2

**2.1. Were participants aware of their assigned**	**2.2. Were carers and people delivering the interventions**	**2.3. If Y/PY/NI to 2.1 or 2.2: Were there deviations from the intended**	**2.4 If Y/PY to 2.3: Were these deviations**	**2.5. If Y/PY/NI to 2.4: Were these deviations**	**2.6 Was an appropriate analysis used to estimate the**	**2.7 If N/PN/NI to 2.6: Was there potential for a substantial impact**

intervention during the trial?	aware of participants' assigned intervention during the trial?	intervention that arose because of the trial context?	likely to have affected the outcome?	from intended intervention balanced between groups?	effect of assignment to intervention?	(on the result) of the failure to analyse participants in the group to which they were randomized?
**Y:** Intervention group received a low-calorie diet intervention before IVF, while control group started IVF immediately: "The study was not powered to detect a small increase in live births due to weight reduction and was not blinded for the patients or physician."	**Y:** Intervention group received a low-calorie diet intervention before IVF, while control group started IVF immediately: "The study was not powered to detect a small increase in live births due to weight reduction and was not blinded for the patients or physician."	**PN:** No information given, but no evidence that there were deviations from the intended intervention due to trial context.	**N/A**	**N/A**	**PY:** "The main analysis was performed on the full analysis set (FAS) population and consisted of all randomized women having at least one follow-up variable and having started the IVF treatment (defined as having started stimulation with follitropin alfa) or having achieved a spontaneous pregnancy."	**N/A**

Domain 3

3.1 Were data for this outcome available for all, or nearly all, participants randomized?	3.2 If N/PN/NI to 3.1: Is there evidence that the result was not biased by missing outcome data?	3.3 If N/PN to 3.2: Could missingness in the outcome depend on its true value?	3.4 If Y/PY/NI to 3.3: Is it likely that missingness in the outcome depended on its true value?
**Y:** Randomised N=317<br>Full analysis done in n=305<br>Therefore participants with missing outcome data n=12<br>Clinical pregnancy events = 100<br>Live birth events = 87<br><br>Number of events (clinical pregnancies, live births) much greater than number of people with missing outcome data.	**N/A**	**N/A**	**N/A**

Domain 4

4.1 Was the method of measuring the outcome inappropriate?	4.2 Could measurement or ascertainment of the outcome have differed between intervention groups?	4.3 If N/PN/NI to 4.1 and 4.2: Were outcome assessors aware of the intervention received by study participants?	4.4 If Y/PY/NI to 4.3: Could assessment of the outcome have been influenced by knowledge of intervention received?	4.5 If Y/PY/NI to 4.4: Is it likely that assessment of the outcome was influenced by knowledge of intervention received?
**N:** Live births are objective. In footnotes of Table II, they also mention that clinical pregnancy is defined as "Amniotic sac, with or without fetus, observed at sonography in gestational week 7",	**Y:** Method of assessment is the same, however --<br><br>Intervention group:<br>Weight loss phase: 12 weeks<br>Weight control phase: 2 to 5 weeks, i.e. ~4 weeks<br>Then they had IVF	**N/A**	**N/A**	**N/A**

therefore appropriate method.	Control group:<br>Immediate IVF after randomisation<br><br>Pregnancy was tested 14 days after embryo transfer in the IVF process. Therefore, if the intervention period was up to 16 weeks, then the pregnancy test was done up to 18 weeks from baseline, whereas in the control group, pregnancy test was done soon after randomisation.			

**Domain 5**		
**5.1 Were the data that produced this result analysed in accordance with a pre-specified analysis plan that was finalized before unblinded outcome data were available for analysis?**	**5.2 Is the numerical result being assessed likely to have been selected, on the basis of the results, from...multiple eligible outcome measurements (e.g. scales, definitions, time points) within the outcome domain?**	**5.3 Is the numerical result being assessed likely to have been selected, on the basis of the results, from...multiple eligible analyses of the data?**
**Y:** Analysis according to published protocol.	**N:** There is only one possible way in which the outcome domain can be measured (hence there is no opportunity to select from multiple measures).	**N:** There is only one possible way in which the outcome domain can be measured (hence there is no opportunity to select from multiple measures).

**Espinos 2017**		
**Domain 1**		
**1.1 Was the allocation sequence random?**	**1.2 Was the allocation sequence concealed until participants were enrolled and assigned to interventions?**	**1.3 Did baseline differences between intervention groups suggest a problem with the randomization process?**
**Y:** "A physician (A.P.) checked the patients for eligibility and these were then randomly allocated to the study or control group using a computer-generated list."	**NI:** The old Cochrane tool specifies that allocation concealment and blinding are different, but this was the only information available on pre- and post-randomisation concealment: "The team of fertility specialists was blinded to the group assignment."	**N:** "The baseline characteristics of the women in the two groups did not show statistically significant differences in age, BMI, body composition, menstrual history, infertility factors, duration of infertility, antral follicle count, or prevalence of polycystic ovarian morphology (PCOM) (Table 1)."

**Domain 2**						
**2.1. Were participants aware of their assigned intervention during the trial?**	**2.2. Were carers and people delivering the interventions aware of participants' assigned intervention during the trial?**	**2.3. If Y/PY/NI to 2.1 or 2.2: Were there deviations from the intended intervention that arose because of the trial context?**	**2.4 If Y/PY to 2.3: Were these deviations likely to have affected the outcome?**	**2.5. If Y/PY/NI to 2.4: Were these deviations from intended intervention balanced between groups?**	**2.6 Was an appropriate analysis used to estimate the effect of assignment to intervention?**	**2.7 If N/PN/NI to 2.6: Was there potential for a substantial impact (on the result) of the failure to analyse participants in the group to which they were randomized?**
**Y:** "The participants were randomized into two groups: a study group that underwent a 12-week diet and	**Y:** "The participants were randomized into two groups: a study group that underwent a 12-week diet and exercise	**PN:** Not enough information, BUT "dietary intake was assessed by self-reporting every 15 days and if no weight loss was	**N/A**	**N/A**	**Y:** "Primary analyses were performed on an intention-to-treat basis."	**N/A**

exercise programme before starting an IVF/ICSI cycle and a control group who started with no previous interventions."	programme before starting an IVF/ICSI cycle and a control group who started with no previous interventions."	observed at the follow-up visit, the diet was re-evaluated by the dietician and adjusted accordingly."				

Domain 3

**3.1 Were data for this outcome available for all, or nearly all, participants randomized?**	**3.2 If N/PN/NI to 3.1: Is there evidence that the result was not biased by missing outcome data?**	**3.3 If N/PN to 3.2: Could missingness in the outcome depend on its true value?**	**3.4 If Y/PY/NI to 3.3: Is it likely that missingness in the outcome depended on its true value?**
**Y:** a) number of people randomised: 21 treatment, 20 control<br>b) number of people included in the analysis: 21 treatment, 20 control<br>"Primary analyses were performed on an intention-to-treat basis."<br>c) number of people not included in the analysis: 0<br>d) number of events:<br>live birth -- 11 treatment, 6 control<br>pregnancy -- 12 treatment, 7 control	**N/A**	**N/A**	**N/A**

Domain 4

**4.1 Was the method of measuring the outcome inappropriate?**	**4.2 Could measurement or ascertainment of the outcome have differed between intervention groups?**	**4.3 If N/PN/NI to 4.1 and 4.2: Were outcome assessors aware of the intervention received by study participants?**	**4.4 If Y/PY/NI to 4.3: Could assessment of the outcome have been influenced by knowledge of intervention received?**	**4.5 If Y/PY/NI to 4.4: Is it likely that assessment of the outcome was influenced by knowledge of intervention received?**
**N:** Live births are objective. They also define clinical pregnancy as "ultrasound visualization of a gestational sac."	**Y:** "...our control group did not have to wait for 12 weeks to undergo IVF following inclusion in the study."<br>Both groups were assessed on the primary outcome of pregnancy rate, however, the intervention group underwent a 12-week diet and weight programme whereas the control group started IVF immediately. Therefore, the time points at measurement were not comparable.	**N/A**	**N/A**	**N/A**

Domain 5

**5.1 Were the data that produced this result analysed in accordance with a pre-specified analysis plan that was finalized before unblinded outcome data were available for analysis?**	**5.2 Is the numerical result being assessed likely to have been selected, on the basis of the results, from...multiple eligible outcome measurements (e.g. scales, definitions, time points) within the outcome domain?**	**5.3 Is the numerical result being assessed likely to have been selected, on the basis of the results, from...multiple eligible analyses of the data?**
**PY:** Cannot locate protocol. However, on the trial registration page mentioned that they will analyse pregnancy rates by cycle, which they have done.	**N:** There is only one way to measure live births and the authors define clinical pregnancy rate as "ultrasound visualization of a gestational sac."	**N:** There is only one way to measure live births and the authors define clinical pregnancy rate as "ultrasound visualization of a gestational sac."

**Li 2022**		
**Domain 1**		
**1.1 Was the allocation sequence random?**	**1.2 Was the allocation sequence concealed until participants were enrolled and assigned to interventions?**	**1.3 Did baseline differences between intervention groups suggest a problem with the randomization process?**
**Y:** "A randomization list was generated using Statistics Analysis System."	**NI**	**N:** No differences in baseline characteristics from Table 1.

**Domain 2**						
**2.1. Were participants aware of their assigned intervention during the trial?**	**2.2. Were carers and people delivering the interventions aware of participants' assigned intervention during the trial?**	**2.3. If Y/PY/NI to 2.1 or 2.2: Were there deviations from the intended intervention that arose because of the trial context?**	**2.4 If Y/PY to 2.3: Were these deviations likely to have affected the outcome?**	**2.5. If Y/PY/NI to 2.4: Were these deviations from intended intervention balanced between groups?**	**2.6 Was an appropriate analysis used to estimate the effect of assignment to intervention?**	**2.7 If N/PN/NI to 2.6: Was there potential for a substantial impact (on the result) of the failure to analyse participants in the group to which they were randomized?**
**Y:** Two different drugs in different doses were trialed.	**Y:** Two different drugs in different doses were trialed.	**PN:** "After randomization, 147 patients who completed the first stage treatment were proceed to second stage treatment and included in PP analysis (Fig. 1). In MET group, 2 patients discontinued intervention for moderate gastrointestinal discomfort, and 3 patients were lost to follow-up in the first 12 weeks. In EXE group, 5 patients failed to complete the study (3 for moderate gastrointestinal discomfort and 2 for subcutaneous induration with rash at the injection site), and 3 patients were lost to follow-up in the first stage of treatment (Fig. 1). However, discontinuation rate was still similar in both groups (5 of 80 women [6.3%] vs. 8 of 80	**N/A**	**N/A**	**N:** For the outcomes of interest, the data are only given per protocol.	**PN:** Per protocol data for 90% of people randomised. In terms of number of pregnancies or live births, even a few events more or less can impact the results/conclusions, however, there were far more pregnancies than missing data (e.g. question 3.1), therefore, it likely did not have a substantial impact on the result.

		women [10.0%], χ2=0.754, p=0.385)."				

Domain 3

3.1 Were data for this outcome available for all, or nearly all, participants randomized?	3.2 If N/PN/NI to 3.1: Is there evidence that the result was not biased by missing outcome data?	3.3 If N/PN to 3.2: Could missingness in the outcome depend on its true value?	3.4 If Y/PY/NI to 3.3: Is it likely that missingness in the outcome depended on its true value?
**PY:** a) number of people randomised: 80 treatment, 80 control<br>b) number of people included in the analysis: 72 treatment, 75 control<br>c) number of people not included in the analysis: 13<br>d) number of events:<br>live birth -- 48 treatment, 47 control<br>pregnancy -- 57 treatment, 57 control	**N/A**	**N/A**	**N/A**

Domain 4

4.1 Was the method of measuring the outcome inappropriate?	4.2 Could measurement or ascertainment of the outcome have differed between intervention groups?	4.3 If N/PN/NI to 4.1 and 4.2: Were outcome assessors aware of the intervention received by study participants?	4.4 If Y/PY/NI to 4.3: Could assessment of the outcome have been influenced by knowledge of intervention received?	4.5 If Y/PY/NI to 4.4: Is it likely that assessment of the outcome was influenced by knowledge of intervention received?
**N:** Live births are objective.<br>"Pregnancy was dated by any mode of conception with a singleton viable fetus (determined by transvaginal ultrasound) between gestational week 6 and week 9 plus 6 days."	**PN:** Method of measurement/ascertainment was the same.<br>In terms of follow-up timelines:<br>Phase 1: exenatide bid for 12 weeks; were followed up every 4 weeks<br><br>Phase 2: metformin bid for 12 weeks; If no pregnancy at week 24, ovulation induction for 3 cycles; If still no pregnancy, IVF + embryo transfer for one cycle. This stage lasted until patients were pregnant with a viable foetus between week 6 and week 10 within 52 weeks. Medication and follow up ceased when patients did not become pregnant after 64 weeks. Patients were followed up at 4-week intervals until identified pregnancy or week 64."	**Y:** Two different drugs in different doses were trialed.	**Y:** In terms of clinical pregnancy assessment, there is theoretically the possibility of assessors to read the ultrasound differently if they are biased, however, since ultrasound is used, this is unlikely.	**PN:**<br>In terms of clinical pregnancy assessment, there is theoretically the possibility of assessors to read the ultrasound differently if they are biased, however, since ultrasound is used, this is unlikely.

Domain 5

5.1 Were the data that produced this result analysed in accordance with a pre-specified analysis plan that was finalized before unblinded outcome data were available for analysis?	5.2 Is the numerical result being assessed likely to have been selected, on the basis of the results, from...multiple eligible outcome measurements (e.g. scales, definitions, time points) within the outcome domain?	5.3 Is the numerical result being assessed likely to have been selected, on the basis of the results, from...multiple eligible analyses of the data?

**NI:** No protocol available, and no information on trial registration page.	**N:** There is only one way to measure live births, and pregnancies were assessed via ultrasound.	**Y:** They chose to report pregnancies and live births only per protocol, whereas they could also have done ITT analysis.

Moran 2011

Domain 1

**1.1 Was the allocation sequence random?**	**1.2 Was the allocation sequence concealed until participants were enrolled and assigned to interventions?**	**1.3 Did baseline differences between intervention groups suggest a problem with the randomization process?**
**Y:** "Women were randomised using a computer generated randomisation sequence to active dietary modification and exercise or standard treatment, with age stratification."	**NI**	**N:** Differences between baseline characteristics can be found in Table 1 - no significant differences. Dropout rate (not included in Table 1) is also not significantly different between treatment groups. "...(respective dropout rate 14 and 20%, P = 0.710 for difference between treatments). Baseline characteristics are reported in Table 1 with no differences between groups."

Domain 2

**2.1. Were participants aware of their assigned intervention during the trial?**	**2.2. Were carers and people delivering the interventions aware of participants' assigned intervention during the trial?**	**2.3. If Y/PY/NI to 2.1 or 2.2: Were there deviations from the intended intervention that arose because of the trial context?**	**2.4 If Y/PY to 2.3: Were these deviations likely to have affected the outcome?**	**2.5. If Y/PY/NI to 2.4: Were these deviations from intended intervention balanced between groups?**	**2.6 Was an appropriate analysis used to estimate the effect of assignment to intervention?**	**2.7 If N/PN/NI to 2.6: Was there potential for a substantial impact (on the result) of the failure to analyse participants in the group to which they were randomized?**
**Y: Treatment group** activities: "Active treatment consisted of a nutritionally adequate reduced energy diet (5368 kJ, 99.7 g protein, 35.9 g fat, 10.6 g saturated fat, 128.8 g carbohydrate, 23.2 g fibre) consistent with the CSIRO Total Wellbeing Diet. One daily meal was replaced with the liquid meal replacement Optifast (Novartis Consumer Health, Mulgrave, Vic., Australia) and 200 mL reduced fat milk (1057 kJ, 25.4 g protein, 5.2 g fat, 26 g CHO) with Optifast provided to all women. The exercise	**Y: Treatment group** activities: "Active treatment consisted of a nutritionally adequate reduced energy diet (5368 kJ, 99.7 g protein, 35.9 g fat, 10.6 g saturated fat, 128.8 g carbohydrate, 23.2 g fibre) consistent with the CSIRO Total Wellbeing Diet. One daily meal was replaced with the liquid meal replacement Optifast (Novartis Consumer Health, Mulgrave, Vic., Australia) and 200 mL reduced fat milk (1057 kJ, 25.4 g protein, 5.2 g fat, 26 g CHO) with Optifast provided to all women. The exercise	**PN:** No information given, but there is no evidence of deviation due to the trial context, apart from 2 people who could not comply with the assigned intervention. Also, "Participants were actively encouraged to contact investigators to report progress and seek follow-up".	**N/A**	**N/A**	**PY:** ITT for pregnancy and both an ITT and completers analysis for live birth - however it is not always clear when they are reporting from the ITT and when from the completers analysis.<br><br>"Intention to treat (ITT) analysis was conducted for pregnancy, and live birth was assessed by using a completers analysis (analysing only subjects who competed the study) and a baseline ITT analysis	**N/A**

component consisted of a homebased physical conditioning and walking program...3 Women received one initial education visit, one face-to-face follow-up two weeks later and one phone follow-up a further two weeks later and were actively encouraged to contact investigators to report progress and seek follow-up."<br><br>vs **control group**: "The standard treatment group consisted of standard advice on appropriate diet and lifestyle factors influencing fertility provided face-to-face at one session with no active follow-up."	component consisted of a homebased physical conditioning and walking program...3 Women received one initial education visit, one face-to-face follow-up two weeks later and one phone follow-up a further two weeks later and were actively encouraged to contact investigators to report progress and seek follow-up."<br><br>vs **control group**: "The standard treatment group consisted of standard advice on appropriate diet and lifestyle factors influencing fertility provided face-to-face at one session with no active follow-up."				(including dropouts with the baseline values from dropouts carried forward) or a carried-forward ITT analysis (including dropouts with the last clinical value from dropouts carried forward)."<br><br>"There were no significant differences in any variable for ITT analysis for live birth."	

**Domain 3**			
**3.1 Were data for this outcome available for all, or nearly all, participants randomized?**	**3.2 If N/PN/NI to 3.1: Is there evidence that the result was not biased by missing outcome data?**	**3.3 If N/PN to 3.2: Could missingness in the outcome depend on its true value?**	**3.4 If Y/PY/NI to 3.3: Is it likely that missingness in the outcome depended on its true value?**
**PY:** d is larger than c, however it is a small sample to have 7 people missing.<br><br>a) number of people randomised: 21 treatment, 25 control<br>b) number of people included in the analysis: 18 treatment, 20 control<br>c) number of people not included in the analysis: 3 treatment, 4 control<br>d) number of events:<br>live birth - 7 treatment, 5 control<br>pregnancy - 12 treatment, 8 control	**N/A**	**N/A**	**N/A**

**Domain 4**				
**4.1 Was the method of measuring the outcome inappropriate?**	**4.2 Could measurement or ascertainment of the outcome have differed between intervention groups?**	**4.3 If N/PN/NI to 4.1 and 4.2: Were outcome assessors aware of the intervention received by study participants?**	**4.4 If Y/PY/NI to 4.3: Could assessment of the outcome have been influenced by**	**4.5 If Y/PY/NI to 4.4: Is it likely that assessment of the outcome was influenced by knowledge of intervention received?**

			**knowledge of intervention received?**	
**NI:** Live birth is an objective measure. On the other hand, this paper does not explain how they measured pregnancy. Additionally, no distinction is made between clinical and biochemical pregnancies. Given the study took place in a centre for reproductive health, it is likely that the measure was appropriate - however the information is not available.	**PN:** "For all women, the intervention was commenced at the start of the period prior to the IVF cycle (i.e. total therapy of 5–9 weeks before oocyte pick-up)."<br><br>The time to IVF was balanced, however, they do not specify for how long after the IVF people were followed-up for. Presumably there is a point where they just had to stop the study, but this is not specified. There is no evidence however that this might have differed by group.	**Y:** Low calorie diet vs standard advice	**Y:** Particularly because this is a study comparing a strict reduced energy diet to standard care (as opposed to another experimental diet), it is likely that if the assessors were unblind, they would be influenced by the knowledge of patient allocation.	**PN:** Given that significant differences were not found between the groups, it seems unlikely that knowledge of the intervention has influenced the results. There is no significant difference between live birth (p=0.483) or pregnancies (p=0.119) between groups.

**Domain 5**		
**5.1 Were the data that produced this result analysed in accordance with a pre-specified analysis plan that was finalized before unblinded outcome data were available for analysis?**	**5.2 Is the numerical result being assessed likely to have been selected, on the basis of the results, from...multiple eligible outcome measurements (e.g. scales, definitions, time points) within the outcome domain?**	**5.3 Is the numerical result being assessed likely to have been selected, on the basis of the results, from...multiple eligible analyses of the data?**
**NI:** No protocol available, and no results when I search for the trial on the trial registry.	**PN:** There is only one way to measure live births - however the authors do not provide a definition for pregnancy nor do they offer a distinction between clinical and biochemical pregnancy.	**PN**

**Price 2020**		
**Domain 1**		
**1.1 Was the allocation sequence random?**	**1.2 Was the allocation sequence concealed until participants were enrolled and assigned to interventions?**	**1.3 Did baseline differences between intervention groups suggest a problem with the randomization process?**
**Y:** "Computer-generated block randomization within strata based<br>on age, BMI, and parity was used to allocate women to a 12-<br>week intervention: a standard dietary intervention (SDI) or a<br>VLED."	**NI**	**N:** "As noted in Table 1, there were no significant differences between the SDI and VLED groups at baseline."

**Domain 2**						
**2.1. Were participants aware of their assigned intervention during the trial?**	**2.2. Were carers and people delivering the interventions aware of participants' assigned intervention during the trial?**	**2.3. If Y/PY/NI to 2.1 or 2.2: Were there deviations from the intended intervention that arose because of the trial context?**	**2.4 If Y/PY to 2.3: Were these deviations likely to have affected the outcome?**	**2.5. If Y/PY/NI to 2.4: Were these deviations from intended intervention balanced between groups?**	**2.6 Was an appropriate analysis used to estimate the effect of assignment to intervention?**	**2.7 If N/PN/NI to 2.6: Was there potential for a substantial impact (on the result) of the failure to analyse participants in the group to which**

						**they were randomized?**
**PY:** This was a behavioural trial necessitating that patients are aware of their intervention. Furthermore, more women in the standard dietary intervention dropped out. However, patients in the standard dietary intervention were still on a reduced energy diet and may not have known they were in the control group.	**Y:** "Women in the SDI group were instructed to eat an energy-reduced diet. A dietitian calculated current caloric intake based on the basal metabolic rate (calculated by the Harris Benedict equation) and physical activity level...Women in the VLED group were instructed to eat a VLED program consisting of two daily meals of a VLED formulation (Optifast VLCD, Nestle) and to eat a third meal consisting of 2 cups of low-starch vegetables, 150 g lean meat, and 2 teaspoons of oil."	**PN:** Methods consistent with protocol (Price et al., 2018).<br><br>However authors do not give information about for example adherence of the participants to the intervention. However, there is no evidence of deviation from the intended intervention due to the trial context.	**N/A**	**N/A**	**PY:** The authors say they used an ITT analysis: "analysis was performed on an intention-to-treat basis according to the treatment group at randomization." However they also say: "Any pregnancy conceived during the 12-week intervention was excluded from analysis."<br>All patients were included as long as they had the opportunity to receive the intervention.	**N/A**

Domain 3

**3.1 Were data for this outcome available for all, or nearly all, participants randomized?**	**3.2 If N/PN/NI to 3.1: Is there evidence that the result was not biased by missing outcome data?**	**3.3 If N/PN to 3.2: Could missingness in the outcome depend on its true value?**	**3.4 If Y/PY/NI to 3.3: Is it likely that missingness in the outcome depended on its true value?**
**Y:** a) number of people randomised: 164<br>b) number of people included in the analysis: 164<br>c) number of people not included in the analysis: 0<br>d) number of events: 54 treatment pregnancies, 33 control pregnancies<br><br>Note: In this ITT analysis, it looks like they included both 'completers' and 'non-completers,' in addition to a separate analysis with only completers.	**N/A**	**N/A**	**N/A**

Domain 4

**4.1 Was the method of measuring the outcome inappropriate?**	**4.2 Could measurement or ascertainment of the outcome have differed between intervention groups?**	**4.3 If N/PN/NI to 4.1 and 4.2: Were outcome assessors aware of the intervention received by study participants?**	**4.4 If Y/PY/NI to 4.3: Could assessment of the outcome have been influenced by knowledge of intervention received?**	**4.5 If Y/PY/NI to 4.4: Is it likely that assessment of the outcome was influenced by knowledge of intervention received?**
**N:** Women either go into the study site every 12 weeks for a urine test or do	**N:** All patients were on the same timeline: 12 week intervention and then four	**PY:** Because this was a behavioural intervention, patients	**PY:** Pregnancies were confirmed through an ultrasound, however, it	**PN:** Pregnancies were confirmed through an ultrasound, however, it is

an at home pregnancy test and advise the study site if it was positive.<br><br>They also mention that "The date of conception was a derived date, taking into consideration the date of the last menstrual period and/or a date of conception based on an early obstetric ultrasound." Therefore they confirmed the pregnancies in an objective way.	week weight maintenance: "all women who completed the 12-week intervention and conceived between week 13 and week 60 were considered in the analysis" Big time frame, however, it was consistent between the groups.	were likely aware however those analysing the urine samples at the study site were likely not aware.<br><br>Meanwhile, pregnancies were verified by ultrasound, and it is likely that assessors were aware, due to the behavioural nature of the intervention.	is theoretically possible to be biased when you know the allocation, but since it's an ultrasound, it is probably not likely.	theoretically possible to be biased when you know the allocation, but since it's an ultrasound, it is probably not likely.

**Domain 5**		
**5.1 Were the data that produced this result analysed in accordance with a pre-specified analysis plan that was finalized before unblinded outcome data were available for analysis?**	**5.2 Is the numerical result being assessed likely to have been selected, on the basis of the results, from...multiple eligible outcome measurements (e.g. scales, definitions, time points) within the outcome domain?**	**5.3 Is the numerical result being assessed likely to have been selected, on the basis of the results, from...multiple eligible analyses of the data?**
**Y:** Consistent with published protocol. https://pubmed.ncbi.nlm.nih.gov/29690917/	**N:** Pregnancies were confirmed by ultrasound. Strictly speaking, there were multiple measures available: either study site urine tests or at-home pregnancy tests. Based on the rest of the paper however, it seems unlikely that this measure was choosen selectively.	**N:** There were no multiple eligible analyses, and they only analysed ITT.
**Salamun 2018**		
**Domain 1**		
**1.1 Was the allocation sequence random?**	**1.2 Was the allocation sequence concealed until participants were enrolled and assigned to interventions?**	**1.3 Did baseline differences between intervention groups suggest a problem with the randomization process?**
**NI:** "Patients were randomized to one of the two treatment arms." No information about how patients were randomized given.	**NI:** "PCOS patients diagnosed according to the revised Rotterdam criteria (2) participated in a 12-week open-label prospective randomized outpatient clinical study"<br><br>Open label refers to un-blinding. It does not refer to how the allocation was concealed until it was revealed.	**N:** "There were no significant differences in patients' characteristics of infertility history between the treatment groups (Table 1). There were no significant differences at baseline in any of the anthropometric, metabolic and endocrine parameters between the groups except in 120min overload insulin levels (Table 2)."

**Domain 2**						
**2.1. Were participants aware of their assigned intervention during the trial?**	**2.2. Were carers and people delivering the interventions aware of participants' assigned intervention during the trial?**	**2.3. If Y/PY/NI to 2.1 or 2.2: Were there deviations from the intended intervention that arose because of the trial context?**	**2.4 If Y/PY to 2.3: Were these deviations likely to have affected the outcome?**	**2.5. If Y/PY/NI to 2.4: Were these deviations from intended intervention balanced between groups?**	**2.6 Was an appropriate analysis used to estimate the effect of assignment to intervention?**	**2.7 If N/PN/NI to 2.6: Was there potential for a substantial impact (on the result) of the failure to analyse participants in the group to which they were randomized?**

**Y:** "PCOS patients diagnosed according to the revised Rotterdam criteria (2) participated in a 12-week open-label prospective randomized outpatient clinical study"	**Y:** "PCOS patients diagnosed according to the revised Rotterdam criteria (2) participated in a 12-week open-label prospective randomized outpatient clinical study"	**PN:** Because patient engagement is clearly reported, it seems unlikely that there were deviations to the protocol that wouldn't be expected outside a trial context. However, one patient left after a protocol violation. "Twenty-eight patients started the study, and 27 (14 on MET, 13 on COMBI) completed the treatment according to the protocol and were included in the analysis (Fig. 1). One patient in the COMBI group discontinued the study because of protocol violation, 2 patients in the MET group refused IVF. One patient in the COMBI and 1 in the MET group conceived spontaneously immediately after the completed treatment. The remaining 23 patients (11 in the MET and 12 in the COMBI group) attended an IVF after the completed treatment."	**N/A**	**N/A**	**PY:** The authors used all but one randomised patient in their analysis. It is not reported why this patient had to leave the study: "One patient in the COMBI group discontinued the study because of protocol violation." However, this analysis is better described as ITT than PP, especially considering that the authors used data from two patients who did not get pregnant spontaneous but choose to decline IVF.	**N/A**

Domain 3			
**3.1 Were data for this outcome available for all, or nearly all, participants randomized?**	**3.2 If N/PN/NI to 3.1: Is there evidence that the result was not biased by missing outcome data?**	**3.3 If N/PN to 3.2: Could missingness in the outcome depend on its true value?**	**3.4 If Y/PY/NI to 3.3: Is it likely that missingness in the outcome depended on its true value?**
**Y:** Only one patient missing from analysis, which is far less than the number of events.<br><br>a) number of people randomised: 28<br>b) number of people included in the analysis: 27<br>c) number of people not included	**N/A**	**N/A**	**N/A**

in the analysis: 1<br>d) number of events: 14 pregnancies			

Domain 4

4.1 Was the method of measuring the outcome inappropriate?	4.2 Could measurement or ascertainment of the outcome have differed between intervention groups?	4.3 If N/PN/NI to 4.1 and 4.2: Were outcome assessors aware of the intervention received by study participants?	4.4 If Y/PY/NI to 4.3: Could assessment of the outcome have been influenced by knowledge of intervention received?	4.5 If Y/PY/NI to 4.4: Is it likely that assessment of the outcome was influenced by knowledge of intervention received?
**N:** The authors clearly define their criteria for assessing clinical pregnancy: "clinical pregnancies [were] confirmed by ultrasound visualization of the fetal cardiac activity."	**N:** The timeline for assessment of clinical pregnancy appears to be the same for both groups.<br>"participated in a 12-week open-label prospective randomized outpatient clinical study."<br>&<br>"The spontaneous PR was followed for 12 months."	**PY:** This is a "a 12-week open-label prospective randomized outpatient clinical study" - patients were aware of their intervention, however, ultrasound technicians may have been blinded to the patient treatment.	**Y:** It is possible, but given the authors' clear working definition for clinical pregnancy, it is unlikely that knowledge of treatment could bias ultrasound technician's assessment of whether the patient was pregnant.<br><br>"...clinical pregnancies [were] confirmed by ultrasound visualization of the fetal cardiac activity."	**Y:** It is possible, but given the authors' clear working definition for clinical pregnancy, it is unlikely that knowledge of treatment could bias ultrasound technician's assessment of whether the patient was pregnant.<br><br>"...clinical pregnancies [were] confirmed by ultrasound visualization of the fetal cardiac activity."

Domain 5

5.1 Were the data that produced this result analysed in accordance with a pre-specified analysis plan that was finalized before unblinded outcome data were available for analysis?	5.2 Is the numerical result being assessed likely to have been selected, on the basis of the results, from...multiple eligible outcome measurements (e.g. scales, definitions, time points) within the outcome domain?	5.3 Is the numerical result being assessed likely to have been selected, on the basis of the results, from...multiple eligible analyses of the data?
**N:** No protocol, but there is a registration page, and the definition of pregnancies (self-reported), does not align with that in the paper (ultrasound). Now this does not mean that the analysis is different, but different definition of the outcome, influences the analysis.	**PN:** The authors used ultrasound visualization of fetal cardiac activity.	**PN:** There were no multiple eligible analyses, and they only analysed ITT.

Sim 2014

Domain 1

1.1 Was the allocation sequence random?	1.2 Was the allocation sequence concealed until participants were enrolled and assigned to interventions?	1.3 Did baseline differences between intervention groups suggest a problem with the randomization process?
**Y:** "Randomization was done by the sequentially numbered,<br>opaque-sealed envelope method (14) by an individual who<br>was independent of the study team."	**Y:** "Randomization was done by the sequentially numbered,<br>opaque-sealed envelope method (14) by an individual who<br>was independent of the study team."	**N**: Baseline differences were<br>not statistically significant between the groups with the exception of a diagnosis of PCOS. When PCOS diagnosis was included as a covariate, results were unchanged, so unadjusted analyses are reported here. Furthermore, there were no statistically significant differences between the groups for ethnicity, marital status, education level, alcohol consumption, tobacco or marijuana use, or type and number of previous fertility treatments undertaken.

Domain 2

2.1. Were participants aware of their assigned intervention during the trial?	2.2. Were carers and people delivering the interventions aware of participants' assigned intervention during the trial?	2.3. If Y/PY/NI to 2.1 or 2.2: Were there deviations from the intended intervention that arose because of the trial context?	2.4 If Y/PY to 2.3: Were these deviations likely to have affected the outcome?	2.5. If Y/PY/NI to 2.4: Were these deviations from intended intervention balanced between groups?	2.6 Was an appropriate analysis used to estimate the effect of assignment to intervention?	2.7 If N/PN/NI to 2.6: Was there potential for a substantial impact (on the result) of the failure to analyse participants in the group to which they were randomized?
**Y:** "The dietitian, midwives, counsellor, fertility fellow and participants were aware of randomization but fertility specialists were not."	**Y:** "The dietitian, midwives, counsellor, fertility fellow and participants were aware of randomization but fertility specialists were not. The fertility fellow who was aware of randomization was not involved with cycle management and did not perform any assisted conception procedures in these patients."	**PY:** No information given, apart from 2 in the intervention group who violated protocol and 5 in the control who refused to attend the visits. So it is possible, but due to the small numbers and the analyses done, it probably did not affect the outcome.	**PN:** No information given, apart from 2 in the intervention group who violated protocol and 5 in the control who refused to attend the visits. So it is possible, but due to the small numbers and the analyses done, it probably did not affect the outcome.	**N/A**	**Y:** "Intention-to-treat analyses were conducted for primary and secondary outcomes."	**N/A**

Domain 3

3.1 Were data for this outcome available for all, or nearly all, participants randomized?	3.2 If N/PN/NI to 3.1: Is there evidence that the result was not biased by missing outcome data?	3.3 If N/PN to 3.2: Could missingness in the outcome depend on its true value?	3.4 If Y/PY/NI to 3.3: Is it likely that missingness in the outcome depended on its true value?
**Y:** a) number of people randomised: 49<br>b) number of people included in the analysis: 48<br>c) number of people not included in the analysis: 1<br>d) number of events:<br>live birth - 12 treatment, 3 control<br>clinical pregnancy - 13 treatment, 3 control	**N/A**	**N/A**	**N/A**

Domain 4

4.1 Was the method of measuring the outcome inappropriate?	4.2 Could measurement or ascertainment of the outcome have differed between intervention groups?	4.3 If N/PN/NI to 4.1 and 4.2: Were outcome assessors aware of the intervention received by study participants?	4.4 If Y/PY/NI to 4.3: Could assessment of the outcome have been influenced by knowledge of intervention received?	4.5 If Y/PY/NI to 4.4: Is it likely that assessment of the outcome was influenced by knowledge of intervention received?
**N:** Live birth is an objective measure and the authors have a clear definition for how they assessed clinical pregnancy: "A clinical	**N:** "Fertility treatment was commenced 12 weeks after study enrolment in both groups."	**N:** "This was a single-centre, evaluator-blinded, randomized controlled trial."<br>AND	**N/A**	**N/A**

pregnancy was defined as a fetal heartbeat at 7-week gestation."	Both groups started treatment after 3 months, and were followed-up until a year after.	"The dietitian, midwives, counsellor, fertility fellow and participants were aware of randomization but fertility specialists were not."		

Domain 5

**5.1 Were the data that produced this result analysed in accordance with a pre-specified analysis plan that was finalized before unblinded outcome data were available for analysis?**	**5.2 Is the numerical result being assessed likely to have been selected, on the basis of the results, from...multiple eligible outcome measurements (e.g. scales, definitions, time points) within the outcome domain?**	**5.3 Is the numerical result being assessed likely to have been selected, on the basis of the results, from...multiple eligible analyses of the data?**
**NI:** No protocol and cannot find the registration page.	**N:** Only one way to define live birth, so no opportunity for multiple measures there, and authors use clear criteria for defining clinical pregnancy: "A clinical pregnancy was defined as a fetal heartbeat at 7-week gestation."<br><br>Also, evaluators were blinded.	**N:** Only one way to define live birth, so no opportunity for multiple measures there, and authors use clear criteria for defining clinical pregnancy: "A clinical pregnancy was defined as a fetal heartbeat at 7-week gestation."<br><br>Also, evaluators were blinded.

Mutsaerts 2016 study

Domain 1

**1.1 Was the allocation sequence random?**	**1.2 Was the allocation sequence concealed until participants were enrolled and assigned to interventions?**	**1.3 Did baseline differences between intervention groups suggest a problem with the randomization process?**
**Y:** "Randomization was performed online and was stratified according to trial center and ovulatory status. The appropriate infertility treatment was determined and recorded before randomization, since blinding of the treatment assignments was not possible."	**PY:** "Randomization was performed online and was stratified according to trial center and ovulatory status. The appropriate infertility treatment was determined and recorded before randomization, since blinding of the treatment assignments was not possible."	**N:** "Baseline characteristics were similar in the two groups (Table 1)."

Domain 2

**2.1. Were participants aware of their assigned intervention during the trial?**	**2.2. Were carers and people delivering the interventions aware of participants' assigned intervention during the trial?**	**2.3. If Y/PY/NI to 2.1 or 2.2: Were there deviations from the intended intervention that arose because of the trial context?**	**2.4 If Y/PY to 2.3: Were these deviations likely to have affected the outcome?**	**2.5. If Y/PY/NI to 2.4: Were these deviations from intended intervention balanced between groups?**	**2.6 Was an appropriate analysis used to estimate the effect of assignment to intervention?**	**2.7 If N/PN/NI to 2.6: Was there potential for a substantial impact (on the result) of the failure to analyse participants in the group to which they were randomized?**
**Y:** It was either weight loss programme or immediate IVF.	**Y:** It was either weight loss programme or immediate IVF.	**PN:** Not really reported, but protocol design suggests enough measures were taken to ensure fidelity and adherence.	**N/A**	**N/A**	**Y:** "Primary analyses were performed on an intention-to-treat basis."	**N/A**

Domain 3

3.1 Were data for this outcome available for all, or nearly all, participants randomized?	3.2 If N/PN/NI to 3.1: Is there evidence that the result was not biased by missing outcome data?	3.3 If N/PN to 3.2: Could missingness in the outcome depend on its true value?	3.4 If Y/PY/NI to 3.3: Is it likely that missingness in the outcome depended on its true value?
**PY:** a) number of people randomised: authors gave us the number of people with IVF, i.e. I think this is the people not necessarily randomised, but those who were included in the analysis. But let's say 137 since we don't have any more data.<br>b) number of people included in the analysis: 137<br>c) number of people not included in the analysis: 0<br>d) number of events: 43 live births, 73 clinical pregnancies	**N/A**	**N/A**	**N/A**

Domain 4

4.1 Was the method of measuring the outcome inappropriate?	4.2 Could measurement or ascertainment of the outcome have differed between intervention groups?	4.3 If N/PN/NI to 4.1 and 4.2: Were outcome assessors aware of the intervention received by study participants?	4.4 If Y/PY/NI to 4.3: Could assessment of the outcome have been influenced by knowledge of intervention received?	4.5 If Y/PY/NI to 4.4: Is it likely that assessment of the outcome was influenced by knowledge of intervention received?
**N:** Live births are objective and clinical pregnancy was defined as: pregnancy in which the gestational sac was visible on ultrasonography.	**Y:** Control group had immediate IVF, whereas intervention group had to wait first. Although outcomes were assessed at a set time point since baseline, in reality the follow-up is shorter for the intervention group.	**N/A**	**N/A**	**N/A**

Domain 5

5.1 Were the data that produced this result analysed in accordance with a pre-specified analysis plan that was finalized before unblinded outcome data were available for analysis?	5.2 Is the numerical result being assessed likely to have been selected, on the basis of the results, from...multiple eligible outcome measurements (e.g. scales, definitions, time points) within the outcome domain?	5.3 Is the numerical result being assessed likely to have been selected, on the basis of the results, from...multiple eligible analyses of the data?
**Y:** Protocol previously published and followed.	**N:** Only one way to define live birth, so no opportunity for multiple measures there, and authors use clear criteria for defining clinical pregnancy.	**N:** Live birth is an objective measure and the authors provide a clear working definition for clinical pregnancy. Plus, there were no multiple eligible analyses and they only did ITT.

Wang 2021

Domain 1

1.1 Was the allocation sequence random?	1.2 Was the allocation sequence concealed until participants were enrolled and assigned to interventions?	1.3 Did baseline differences between intervention groups suggest a problem with the randomization process?
**Y:** "Eligible women were randomly allocated to receive either orlistat or placebo, in a 1:1 ratio, using computer-generated randomization sequences provided by	**PY:** "Study investigators, staff, and participants were all blind to the treatment allocation throughout the trial."	**PN:** "Baseline characteristics were well balanced between the 2 groups in the intention-to-treat set (Table 1)."

statisticians in the data coordinating center at Shandong University."		The authors did not report any statistical testing of the baseline differences.

Domain 2						
**2.1. Were participants aware of their assigned intervention during the trial?**	**2.2. Were carers and people delivering the interventions aware of participants' assigned intervention during the trial?**	**2.3. If Y/PY/NI to 2.1 or 2.2: Were there deviations from the intended intervention that arose because of the trial context?**	**2.4 If Y/PY to 2.3: Were these deviations likely to have affected the outcome?**	**2.5. If Y/PY/NI to 2.4: Were these deviations from intended intervention balanced between groups?**	**2.6 Was an appropriate analysis used to estimate the effect of assignment to intervention?**	**2.7 If N/PN/NI to 2.6: Was there potential for a substantial impact (on the result) of the failure to analyse participants in the group to which they were randomized?**
**N:** "Study investigators, staff, and participants were all blind to the treatment allocation throughout the trial."<br>"The packaging, appearance, taste, and weight of the placebo tablets were indistinguishable from those of the orlistat tablets to ensure blind allocation."<br>Patients in both groups were also prescribed multi-vitamins and lifestyle modifications.	**N:** "Study investigators, staff, and participants were all blind to the treatment allocation throughout the trial."	**N/A**	**N/A**	**N/A**	**PY:** "Primary analysis was conducted in the intention-to-treat population, comprising all randomly assigned participants who received at least one dose of study medication. All spontaneous pregnancies occurring after randomization were included in the intention-to-treat analysis."<br><br>'Probably yes' because the authors refer to this as an ITT however, this only includes patients who received at least one dose - which does not seem like a true ITT. According to their flow chart, 888 patients were randomised but only 877 were included in the primary analysis.	**N/A**

Domain 3			
**3.1 Were data for this outcome available for all, or nearly all, participants randomized?**	**3.2 If N/PN/NI to 3.1: Is there evidence that the result was not biased by missing outcome data?**	**3.3 If N/PN to 3.2: Could missingness in the outcome depend on its true value?**	**3.4 If Y/PY/NI to 3.3: Is it likely that missingness in the outcome depended on its true value?**
**Y:** a) number of people randomised: 888<br>b) number of people included in the analysis: 877<br>c) number of people not included	**N/A**	**N/A**	**N/A**

in the analysis: 11<br>d) number of events: 224 live births, 272 clinical pregnancies			

Domain 4				
**4.1 Was the method of measuring the outcome inappropriate?**	**4.2 Could measurement or ascertainment of the outcome have differed between intervention groups?**	**4.3 If N/PN/NI to 4.1 and 4.2: Were outcome assessors aware of the intervention received by study participants?**	**4.4 If Y/PY/NI to 4.3: Could assessment of the outcome have been influenced by knowledge of intervention received?**	**4.5 If Y/PY/NI to 4.4: Is it likely that assessment of the outcome was influenced by knowledge of intervention received?**
**N:** The authors had clear definitions for both live birth and pregnancy.<br>(1) Live births = "at least one viable neonate at birth"<br>(2) clinical intrauterine pregnancy = "detection of a gestational sac in the uterine cavity by ultrasonography"<br>(3) ongoing pregnancy = "detection of a viable fetus with fetal heartbeat at 11-12 weeks' gestation"	**PN:** Treatment and control groups followed the same schedule to assessment: "The orlistat or placebo treatment was given for 4 to 12 weeks prior to controlled ovarian hyperstimulation (COH) and stopped before the day of fresh ET."<br><br>However, it is unclear for how long each group was followed-up for reproductive outcomes. There is no evidence though that this was different by group, therefore, PN.	**N:** "Study investigators, staff, and participants were all blind to the treatment allocation throughout the trial."	**N/A**	**N/A**

Domain 5		
**5.1 Were the data that produced this result analysed in accordance with a pre-specified analysis plan that was finalized before unblinded outcome data were available for analysis?**	**5.2 Is the numerical result being assessed likely to have been selected, on the basis of the results, from...multiple eligible outcome measurements (e.g. scales, definitions, time points) within the outcome domain?**	**5.3 Is the numerical result being assessed likely to have been selected, on the basis of the results, from...multiple eligible analyses of the data?**
Y: Consistent with published protocol.	**N:** Because of the authors' clear definitions for live birth and clinical pregnancy, there is no room for multiple measures.<br>clinical intrauterine pregnancy = "detection of a gestational sac in the uterine cavity by ultrasonography"	**N:** Because of the authors' clear definitions for live birth and clinical pregnancy, there is no room for multiple measures.<br>clinical intrauterine pregnancy = "detection of a gestational sac in the uterine cavity by ultrasonography"<br><br>Likewise, there were no multiple eligible analyses, and they only did ITT.

Kiel 2018		
**Domain 1**		
**1.1 Was the allocation sequence random?**	**1.2 Was the allocation sequence concealed until participants were enrolled and assigned to interventions?**	**1.3 Did baseline differences between intervention groups suggest a problem with the randomization process?**
**Y:** "Randomization is performed by a web-based randomization system developed and administered by the Unit of Applied Clinical Research, Institute of Cancer Research and Molecular Medicine, NTNU, Trondheim, Norway. The randomization is in blocks with varying block size."	**NI**	**N:** "There were no significant differences between the groups at baseline, except for a lower BMI in the HIT group (28.9±2.4 in the HIT group vs 31.2±1.3 in the control group, p=0.03)."

Lundgren KM, Romundstad LB, During von V, et al. Exercise prior to assisted fertilization in overweight and obese women (FertilEX): study protocol for a randomized controlled trial. Trials 2016;17:268		

Domain 2

2.1. Were participants aware of their assigned intervention during the trial?	2.2. Were carers and people delivering the interventions aware of participants' assigned intervention during the trial?	2.3. If Y/PY/NI to 2.1 or 2.2: Were there deviations from the intended intervention that arose because of the trial context?	2.4 If Y/PY to 2.3: Were these deviations likely to have affected the outcome?	2.5. If Y/PY/NI to 2.4: Were these deviations from intended intervention balanced between groups?	2.6 Was an appropriate analysis used to estimate the effect of assignment to intervention?	2.7 If N/PN/NI to 2.6: Was there potential for a substantial impact (on the result) of the failure to analyse participants in the group to which they were randomized?
**Y:** Because it is a behavioural intervention, patients were likely already aware of their group assignment, however group allocation seemed to affect patient behaviour, leading to a deviation from protocol: "...participants allocated to the control group no longer waited for 10 weeks before the assisted fertilisation treatment started, as we experienced that participants declined to participate if their standard treatment could be delayed."	**Y:** "Participants in the HIT group were encouraged to continue with the exercise during the fertility treatment, until ovulation induction. Participants in the control group received regular advice from the hospital staff about physical activity (usual care)."<br>AND<br>"The investigators were not blinded for group-allocation on measurements of VO2 peak, body composition and height or in intervention administration."	**Y:** "We made the following changes to the protocol after trial initiation: (1) from February 2016, participants allocated to the control group no longer waited for 10 weeks before the assisted fertilisation treatment started, as we experienced that participants declined to participate if their standard treatment could be delayed. (2) From February 2016, we also opened the study to patients from a private fertility clinic in Trondheim (Spiren Fertility Clinic) to increase the recruitment."	**Y:** As a result of the first deviation, the assessment of primary outcomes was not performed in the same timeframe for the treatment and intervention group (leading to a greater risk of bias in RoB criteria 4.2: Could measurement or ascertainment of the outcome have differed between intervention groups?)	**N:** While the new recruitment strategy would have been balanced, the decision to start fertility treatment earlier in control patients but to retain the original timeline for the treatment patients is not balanced.	**Y:** The authors performed an ITT analysis:<br>"All available data were used in both time points."	**N/A**

Domain 3

3.1 Were data for this outcome available for all, or nearly all, participants randomized?	3.2 If N/PN/NI to 3.1: Is there evidence that the result was not biased by missing outcome data?	3.3 If N/PN to 3.2: Could missingness in the outcome depend on its true value?	3.4 If Y/PY/NI to 3.3: Is it likely that missingness in the outcome depended on its true value?
**Y:** a) number of people randomised: 18<br>b) number of people included in the analysis: 18<br>c) number of people not included in the analysis: 0	**N/A**	**N/A**	**N/A**

d) number of events (pregnancies): treatment 4, control 4			

Domain 4

**4.1 Was the method of measuring the outcome inappropriate?**	**4.2 Could measurement or ascertainment of the outcome have differed between intervention groups?**	**4.3 If N/PN/NI to 4.1 and 4.2: Were outcome assessors aware of the intervention received by study participants?**	**4.4 If Y/PY/NI to 4.3: Could assessment of the outcome have been influenced by knowledge of intervention received?**	**4.5 If Y/PY/NI to 4.4: Is it likely that assessment of the outcome was influenced by knowledge of intervention received?**
**N:** Authors are clear about their criteria for the outcome 'ongoing pregnancy': "defined as the sonographic evidence of intrauterine gestational sac and fetal heart activity at week 7 to 8 of gestation."	**PY:** The timeline to fertility treatment and follow-up was different between the two groups: "We made the following changes to the protocol after trial initiation: (1) from February 2016, participants allocated to the control group no longer waited for 10 weeks before the assisted fertilisation treatment started, as we experienced that participants declined to participate if their standard treatment could be delayed."<br><br>‘Probably yes' instead of 'yes' because authors later provide contradictory information, indicating that control patients did in fact wait 10 weeks: "All participants underwent the same assessments at baseline and after the 10 weeks intervention period."	**PN:** If the assessors were the same people delivering the fertility treatment, then the authors indicate that they were not aware of intervention status: "The fertility treatment, blood pressure measurements, blood sampling and hyperinsulinemic euglycaemic clamp assessments were done blinded for group allocation."<br><br>However it may be the case that the ones performing the ultrasounds were not considered part of 'fertility treatment,' therefore 'probably no' instead of 'no.'	**N/A**	**N/A**

Domain 5

**5.1 Were the data that produced this result analysed in accordance with a pre-specified analysis plan that was finalized before unblinded outcome data were available for analysis?**	**5.2 Is the numerical result being assessed likely to have been selected, on the basis of the results, from...multiple eligible outcome measurements (e.g. scales, definitions, time points) within the outcome domain?**	**5.3 Is the numerical result being assessed likely to have been selected, on the basis of the results, from...multiple eligible analyses of the data?**
**N:** "We made the following changes to the protocol after trial initiation: (1) from February 2016, participants allocated to the control group no longer waited for 10 weeks before the assisted fertilisation treatment started, as we experienced that participants declined to participate if their standard treatment could be delayed. (2) From February 2016, we also opened the study to patients from a private fertility clinic in Trondheim (Spiren Fertility Clinic) to increase the recruitment. Changes to the original study protocol were approved by Regional Committee for Medical and Health Research Ethics."	**PN:** "...ongoing pregnancy defined as the sonographic evidence of intrauterine gestational sac and fetal heart activity at week 7 to 8 of gestation."<br>Regardless of whether the outcome is pregnancy rate or ongoing pregnancy, the method of assessing pregnancy is consistent.<br><br>Sonographic evidence is objective.	**PN:** "...ongoing pregnancy defined as the sonographic evidence of intrauterine gestational sac and fetal heart activity at week 7 to 8 of gestation."<br>Regardless of whether the outcome is pregnancy rate or ongoing pregnancy, the method of assessing pregnancy is consistent.<br><br>Sonographic evidence is objective.

Wang 2023

Domain 1

**1.1 Was the allocation sequence random?**	**1.2 Was the allocation sequence concealed until participants were enrolled and assigned to interventions?**	**1.3 Did baseline differences between intervention groups suggest a problem with the randomization process?**
**Y:** "An independent statistician used the block randomisation method (group numbers were 4, the distribution ratio was 1:1:1:1, and the block size was set at 8) to generate a randomised sequence by SAS V9.2 (SAS. Cary, NC, USA)"	**Y:** "The statistician then sealed the random order and group names into opaque envelopes. The randomisation sequence was maintained by the statisticians. The authorized investigator assigned participants to different groups by sequentially opening the envelopes"	**N:** See Table 1

Domain 2

**2.1. Were participants aware of their assigned intervention during the trial?**	**2.2. Were carers and people delivering the interventions aware of participants' assigned intervention during the trial?**	**2.3. If Y/PY/NI to 2.1 or 2.2: Were there deviations from the intended intervention that arose because of the trial context?**	**2.4 If Y/PY to 2.3: Were these deviations likely to have affected the outcome?**	**2.5. If Y/PY/NI to 2.4: Were these deviations from intended intervention balanced between groups?**	**2.6 Was an appropriate analysis used to estimate the effect of assignment to intervention?**	**2.7 If N/PN/NI to 2.6: Was there potential for a substantial impact (on the result) of the failure to analyse participants in the group to which they were randomized?**
**Y:** "We conducted a 2×2 factorial design, randomised, unblinded, external pilot trial."	**Y:** "We conducted a 2×2 factorial design, randomised, unblinded, external pilot trial."	**NI**	**N/A**	**N/A**	**PY:** "Primary analyses were performed on an intention-to-treat basis."	**N/A**

Domain 3

**3.1 Were data for this outcome available for all, or nearly all, participants randomized?**	**3.2 If N/PN/NI to 3.1: Is there evidence that the result was not biased by missing outcome data?**	**3.3 If N/PN to 3.2: Could missingness in the outcome depend on its true value?**	**3.4 If Y/PY/NI to 3.3: Is it likely that missingness in the outcome depended on its true value?**
**PY:** Number of events exceeds amount of participants with missing data - however still unclear from flowchart which people were excluded and if this is true ITT.	**N/A**	**N/A**	**N/A**

Domain 4

**4.1 Was the method of measuring the outcome inappropriate?**	**4.2 Could measurement or ascertainment of the outcome have differed between intervention groups?**	**4.3 If N/PN/NI to 4.1 and 4.2: Were outcome assessors aware of the intervention received by study participants?**	**4.4 If Y/PY/NI to 4.3: Could assessment of the outcome have been influenced by knowledge of intervention received?**	**4.5 If Y/PY/NI to 4.4: Is it likely that assessment of the outcome was influenced by knowledge of intervention received?**
**N:** The primary clinical outcome of the RCT was live birth, defined as the delivery of any viable infant at 28 weeks or more of gestation. If the patient was	**Y:** Intervention group was allowed to start IVF early if they met their weightloss goal: "To enhance adherence to the intervention, once the women achieved their goal, they could start	**N/A**	**N/A**	**N/A**

pregnant, defined as a serum HCG level>10 IU/L, vaginal sonography was performed 4 weeks after ET to confirm clinical pregnancy, and ongoing pregnancy was confirmed by transvaginal ultrasound at approximately 10 weeks of gestation.	their IVF/ICSI treatment even before the end of the 12 weeks"			

**Domain 5**		
**5.1 Were the data that produced this result analysed in accordance with a pre-specified analysis plan that was finalized before unblinded outcome data were available for analysis?**	**5.2 Is the numerical result being assessed likely to have been selected, on the basis of the results, from...multiple eligible outcome measurements (e.g. scales, definitions, time points) within the outcome domain?**	**5.3 Is the numerical result being assessed likely to have been selected, on the basis of the results, from...multiple eligible analyses of the data?**
**Y:** Outcomes in protocol are the same as those reported in the paper.	**N:** No evidence of this and clear reporting for different outcomes (e.g. clinical pregnancy vs ongoing pregnancy).	**N:** No evidence of this.

Weight loss

Collectively, participants across all intervention groups lost approximately 5 kg (weighted mean change -5.2 kg; range -9.1 – -0.6 kg), and comparator groups lost approximately 1 kg (weighted mean change -1.2 kg; range -7.0 – 1.2 kg), resulting in a mean difference of about -4 kg favoring the intervention, albeit with substantial heterogeneity (-4.10 kg, 95% CI -6.43 to -1.77; I^2=94.1%; 11 studies; 1,769 participants) (**Figure S1**).

Weighted mean change in diet and/or physical activity intervention groups was -4.5 kg (range -4.6 – -3.7 kg), compared with weight change of -0.6 kg (range -0.7 – 0.7 kg) in comparators receiving no/minimal intervention, resulting in a mean difference of -4 kg (-3.86kg, 95% CI -5.02 to -2.70; I_2=0.0%; 4 studies; 317 participants randomized). In one study, where the main intervention being tested was an exercise program, the intervention group lost -0.6 kg and the active comparator lost -0.7 kg (mean difference 0.10 kg, 95% CI -3.80 to 4.00; I^2=0.0%; 13 participants) (**Figure S1**).

In one study, the low-energy diet group lost -9.1 kg, and the comparator receiving no/minimal intervention gained 1.2 kg, producing a mean difference of -10.29 kg (95% CI -12.15 to -8.43; I^2=0.0%; kg; 305 participants). Low-energy diets also led to greater weight loss compared to an active comparator. The weighted mean change was -10.0 kg (range -13.0 – -6.6 kg) in the intervention groups and -2.4 kg (range -3.2 – -1.6 kg) in the active comparators, resulting in between-group difference of -7.54 kg (95% CI -37.98 to -22.91; I^2=89.1%; 2 studies; 167 participants) (**Figure S1**).

Pharmacotherapy interventions induced more weight loss (weighted mean change -4.9 kg; range -217 7.5 – -2.5 kg), than active comparators (weighted mean change -3.7 kg; range -7.0 – -1.2 kg), leading to about a kilogram difference (-1.33 kg, 95% CI -2.20 to -0.46; I^2=0.0%; 3 studies; 1,051 participants) (**Figure S1**).

Heterogeneity was substantial in the low-energy diet vs active comparator subgroup, perhaps due to only two included studies contributing data, and differences in comparator intensity. In Sim et al., it was the responsibility of participants in the comparator group to seek weight loss advice from their doctor, and those with a BMI ≥35 kg/m^2 were also referred to a weight loss service, whereas in Price et al., all comparator participants received the same dietary advice.

We did not observe a consistent pattern when sorting the weight change forest plot by the proportion of participants with PCOS in the sample (**Figure S2**). In 2 studies where all participants had pre-existing PCOS, weight loss was lower than the average for all studies, however, this could be because both of these studies used an active comparator also experiencing weight loss.

After excluding studies at high RoB, only 5 studies (1,109 participants) reporting weight outcomes remained, and the difference in weight change between groups – though of similar magnitude to the primary analysis – led to greater heterogeneity and wider confidence intervals (-4 kg, 95% CI -8.64 to 0.64; I^2 = 96.0%) (**Figure S3**).

Figure S1. Mean difference in weight change between intervention and comparator groups, grouped by intervention and comparator type

Study	Intervention (N)	Comparator (N)	Mean age (years)	Baseline BMI (kg/m2)	% PCOS	Mean difference in weight change, kg (95% CI)	% Weight
Diet and/or physical activity vs no/minimal intervention							
Becker 2015	11	12	31.3	28.7	15.4	-5.23 (-7.43, -3.03)	9.18
Moran 2011	18	20	33.1	34	0	-3.30 (-4.79, -1.81)	9.75
Mutsaerts 2016	58	79		35.5		-3.87 (-5.09, -2.65)	9.92
Wang 2023	17	18	30	27.8	0	-3.20 (-7.20, 0.80)	7.34
Subgroup, DL+HKSJ (I^2 = 0.0%, p = 0.543)						-3.86 (-5.02, -2.70)	36.19
Diet and/or physical activity vs active comparator							
Kiel 2018	6	7	32.3	30.2		0.10 (-3.80, 4.00)	7.45
Low-energy diet with/without physical activity advice vs no/minimal intervention							
Einarsson 2017	152	153	31.6	33.1	20.7	-10.29 (-12.15, -8.43)	9.47
Low-energy diet with/without physical activity advice vs active comparator							
Price 2020, 2021	71	53	32.3	38.7	39	-9.80 (-11.33, -8.27)	9.72
Sim 2014	26	17	32.9	36.4	30.6	-5.00 (-7.70, -2.30)	8.69
Subgroup, DL+HKSJ (I^2 = 89.1%, p = 0.002)						-7.54 (-37.98, 22.91)	18.41
Pharmacotherapy with some diet and physical activity advice vs active comparator							
Li 2022	72	75	28	29.1	100	-1.66 (-2.68, -0.64)	10.03
Salamun 2018	13	14	30.6	36.6	100	-0.52 (-3.58, 2.54)	8.34
Wang 2021	439	438	30.7	29.4		-1.18 (-2.00, -0.36)	10.12
Subgroup, DL+HKSJ (I^2 = 0.0%, p = 0.670)						-1.33 (-2.20, -0.46)	28.48
Heterogeneity between groups: p = 0.000							
Overall, DL+HKSJ (I^2 = 94.1%, p = 0.000)						-4.10 (-6.43, -1.77)	100.00

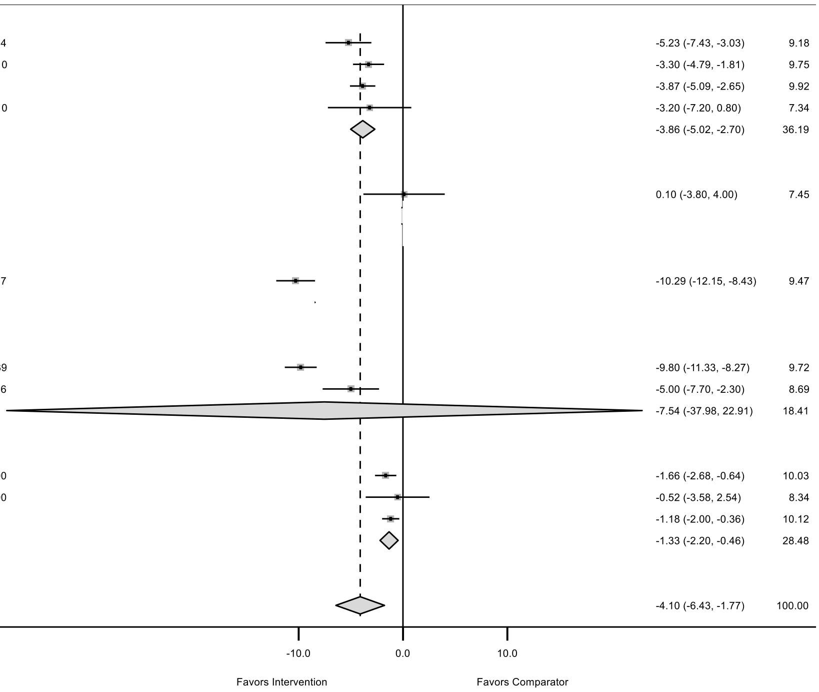

N: number of participants analyzed for weight change; BMI: body mass index; PCOS: polycystic ovarian syndrome; CI: confidence interval; DL: DerSimonian–Laird; HKSJ: Hartung–Knapp–Sidik–Jonkman

In Kiel 2018, the main intervention being tested was a physical activity program, but it was also accompanied by some dietary advice.

Mean age for women specifically seeking in vitro fertilization (IVF) was unknown from Mutsaerts 2016; % PCOS in the sample was unknown from Mutsaerts 2016, Wang 2021, and Kiel 2018, either because it was not reported, or because it was not reported specifically for women seeking IVF; Espinos 2017 did not provide weight data at follow-up for the comparator, therefore it was not possible to calculate the mean difference in weight change between groups; We were able to obtain weight data for women specifically seeking IVF from authors of Mutsaerts 2016; Li 2022 and Price 2020, 2021 reported weight data from per protocol analysis only.

Figure S2. Mean difference in weight change between intervention and comparator groups, sorted by % PCOS in the sample (A: all studies together; B: grouped by intervention and comparator type)

A

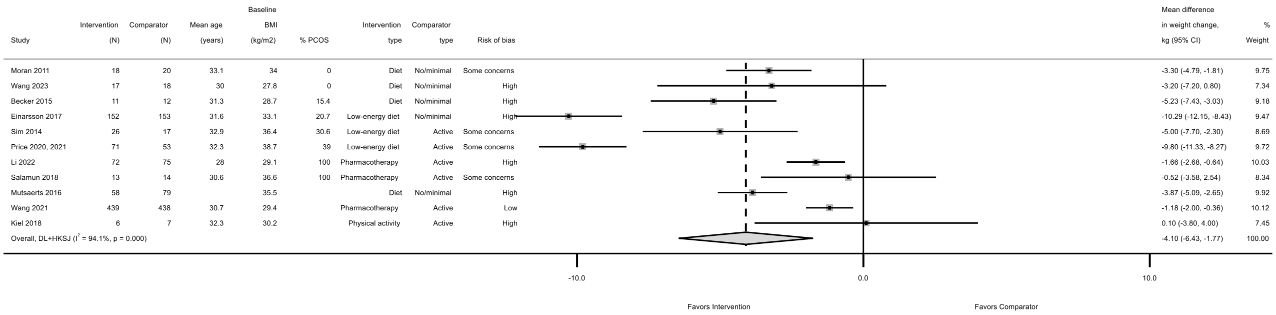

B

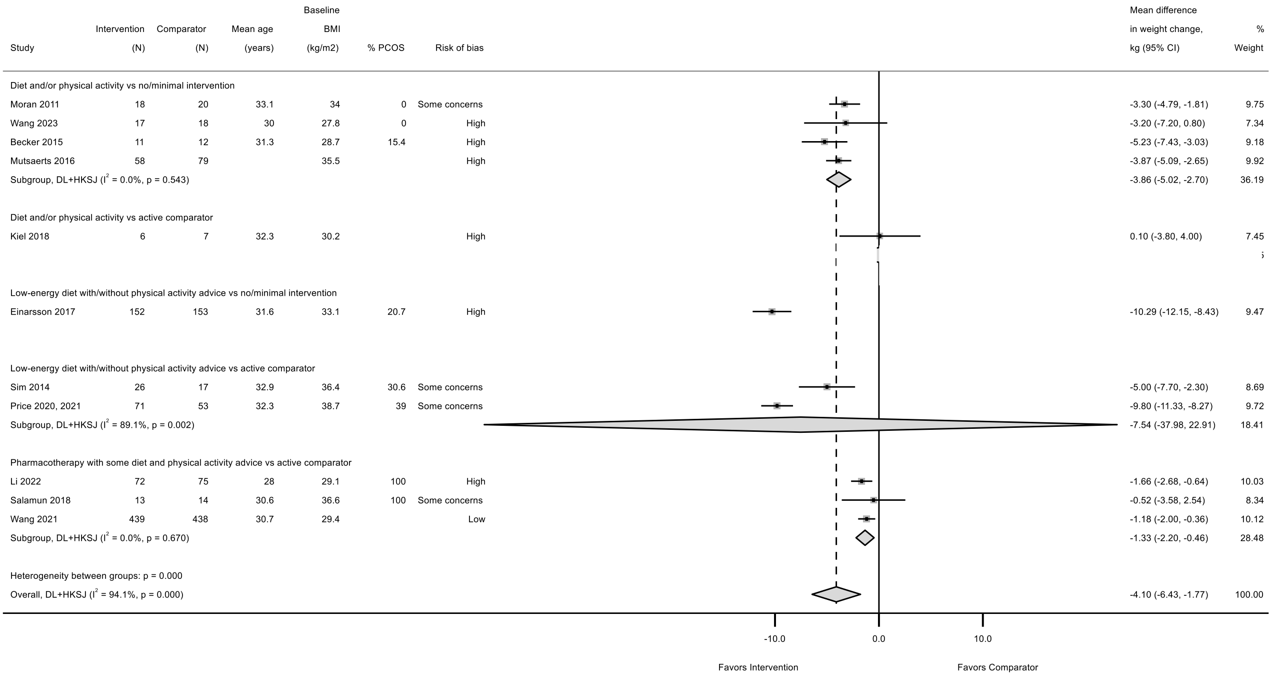

N: number of participants analyzed for weight change; BMI: body mass index; PCOS: polycystic ovarian syndrome; CI: confidence interval; DL: DerSimonian–Laird; HKSJ: Hartung–Knapp–Sidik–Jonkman

In Kiel 2018, the main intervention being tested was a physical activity program, but it was also accompanied by some dietary advice.

Mean age for women specifically seeking in vitro fertilization (IVF) was unknown from Mutsaerts 2016; % PCOS in the sample was unknown from Mutsaerts 2016, Wang 2021, and Kiel 2018, either because it was not reported, or because it was not reported specifically for women seeking IVF; Espinos 2017 did not provide weight data at follow-up for the comparator, therefore it was not possible to calculate the mean difference in weight change between groups; We were able to obtain weight data for women specifically seeking IVF from authors of Mutsaerts 2016; Li 2022 and Price 2020, 2021 reported weight data from per protocol analysis only.

Figure S3. Mean difference in weight change between intervention and comparator groups, grouped by intervention and comparator type, excluding studies at overall high risk of bias

Study	Intervention (N)	Comparator (N)	Mean age (years)	Baseline BMI (kg/m2)	% PCOS	Risk of bias	Mean difference in weight change, kg (95% CI)	% Weight
Diet and/or physical activity vs no/minimal intervention								
Moran 2011	18	20	33.1	34	0	Some concerns	-3.30 (-4.79, -1.81)	20.64
Low-energy diet with/without physical activity advice vs active comparator								
Sim 2014	26	17	32.9	36.4	30.6	Some concerns	-5.00 (-7.70, -2.30)	19.07
Price 2020, 2021	71	53	32.3	38.7	39	Some concerns	-9.80 (-11.33, -8.27)	20.60
Subgroup, DL+HKSJ (I^2 = 89.1%, p = 0.002)							-7.54 (-37.98, 22.91)	39.68
Pharmacotherapy with some diet and physical activity advice vs active comparator								
Salamun 2018	13	14	30.6	36.6	100	Some concerns	-0.52 (-3.58, 2.54)	18.51
Wang 2021	439	438	30.7	29.4		Low	-1.18 (-2.00, -0.36)	21.17
Subgroup, DL+HKSJ (I^2 = 0.0%, p = 0.683)							-1.14 (-3.24, 0.97)	39.68
Heterogeneity between groups: p = 0.001								
Overall, DL+HKSJ (I^2 = 96.0%, p = 0.000)							-4.00 (-8.64, 0.64)	100.00

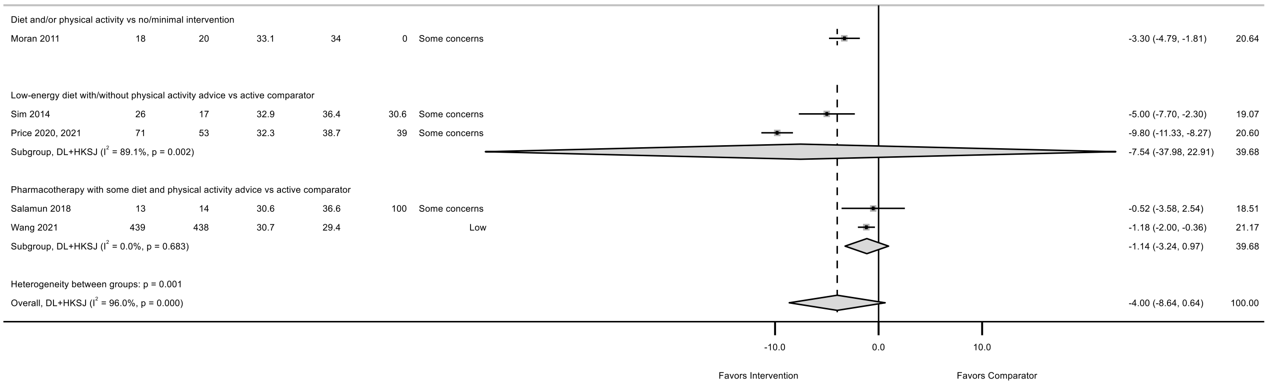

N: number of participants analyzed for weight change; BMI: body mass index; PCOS: polycystic ovarian syndrome; CI: confidence interval; DL: DerSimonian–Laird; HKSJ: Hartung–Knapp–Sidik–Jonkman

% PCOS in the sample was unknown from Wang 2021; Price 2020, 2021 reported weight data from per protocol analysis only.

Figure S4: Risk of bias of included randomized controlled trials

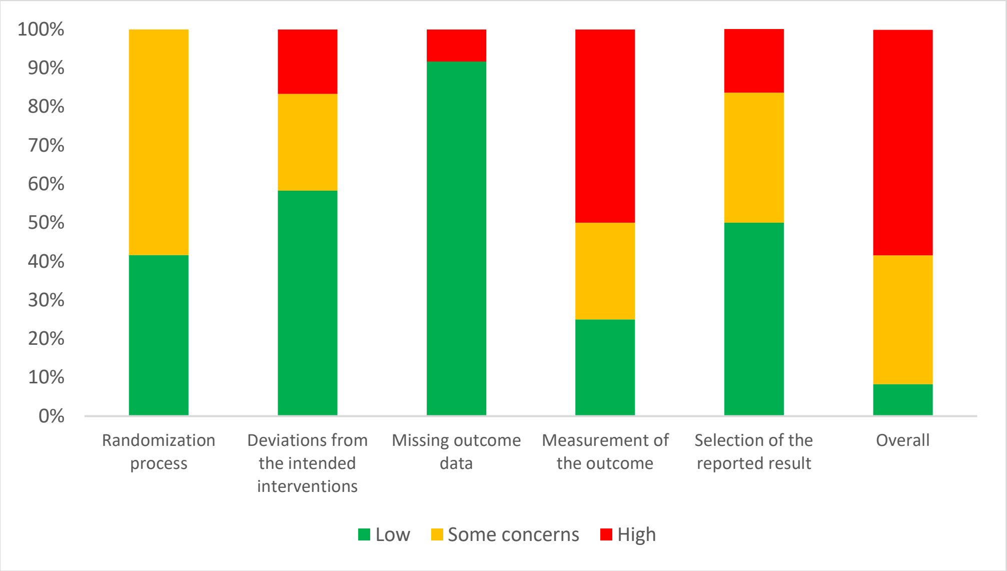

Figure S5. Unassisted pregnancy rates, intervention vs comparator groups, sorted by mean difference in weight change between groups (all studies together)

Study	Intervention (n / N)	Comparator (n / N)	Mean age (years)	Baseline BMI (kg/m2)	% PCOS	Intervention type	Comparator type	Risk of bias	Mean difference in weight change (kg)	Risk Ratio (95% CI)	% Weight, DL+HKSJ
Price 2020, 2021	49 / 85	29 / 79	32.3	38.7	39	Low-energy diet	Active	Some concerns	-9.80	1.50 (1.11, 2.03)	27.23
Becker 2015	3 / 16	0 / 19	31.3	28.7	15.4	Diet	No/minimal	High	-5.23	2.14 (1.23, 3.72)	8.13
Sim 2014	3 / 27	0 / 22	32.9	36.4	30.6	Low-energy diet	Active	Some concerns	-5.00	1.68 (1.06, 2.66)	11.70
Moran 2011	0 / 21	1 / 25	33.1	34	0	Diet	No/minimal	Some concerns	-3.30	0.53 (0.05, 6.02)	0.42
Wang 2023	2 / 18	0 / 20	30	27.8	0	Diet	No/minimal	High	-3.20	1.87 (1.00, 3.48)	6.45
Li 2022	21/ 80	11 / 80	28	29.1	100	Pharmacotherapy	Active	High	-1.66	1.42 (1.04, 1.95)	25.34
Wang 2021	11 / 445	9 / 443	30.7	29.4		Pharmacotherapy	Active	Low	-1.18	1.10 (0.74, 1.64)	15.36
Salamun 2018	3 / 13	3 / 14	30.6	36.6	100	Pharmacotherapy	Active	Some concerns	-0.52	1.05 (0.42, 2.63)	2.95
Kiel 2018	2 / 8	1 / 10	32.3	30.2		Physical activity	Active	High	0.10	1.67 (0.61, 4.59)	2.42
Overall, DL+HKSJ (I^2 = 0.0%, p = 0.651)										1.47 (1.26, 1.73)	100.00
Overall, MH										1.41 (1.19, 1.67)	

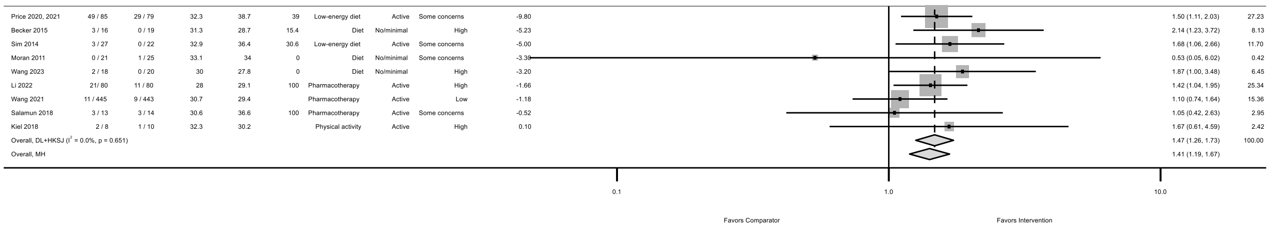

n: number of events; N: number of participants randomized; CI: confidence interval; BMI: body mass index; PCOS: polycystic ovary syndrome; CI: confidence interval; DL: DerSimonian–Laird; HKSJ: Hartung–Knapp–Sidik–Jonkman; MH: Mantel-Haenszel

All but one (Becker 2015) diet interventions were accompanied by physical activity advice; All low-energy diet interventions were accompanied by some physical activity advice; All pharmacotherapy interventions were accompanied by both diet and physical activity advice.

% PCOS in the sample was unknown from Wang 2021 and Kiel 2018; Einarsson 2017 reported only the unassisted pregnancies which led to a live birth (16 in intervention, 4 in comparator), not the total number of unassisted pregnancies, and was not included in this meta-analysis; Espinos 2017 reported 0 events in both groups and was omitted from the meta-analysis; Mutsaerts 2016 did not report unassisted pregnancies for women specifically seeking IVF; Li 2022 reported weight and event data from per protocol analysis only; Price 2020, 2021 reported weight data from per protocol analysis only.

Figure S6. Unassisted pregnancy rates, intervention vs comparator groups, sorted by mean age (A: all studies together; B: grouped by intervention and comparator type)

A

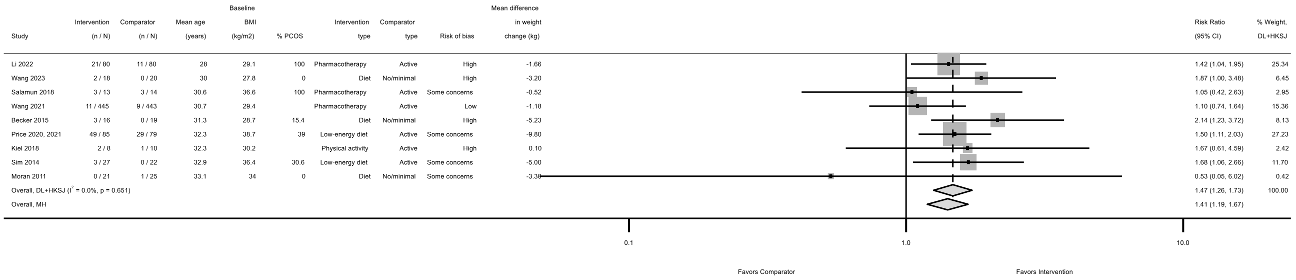

B

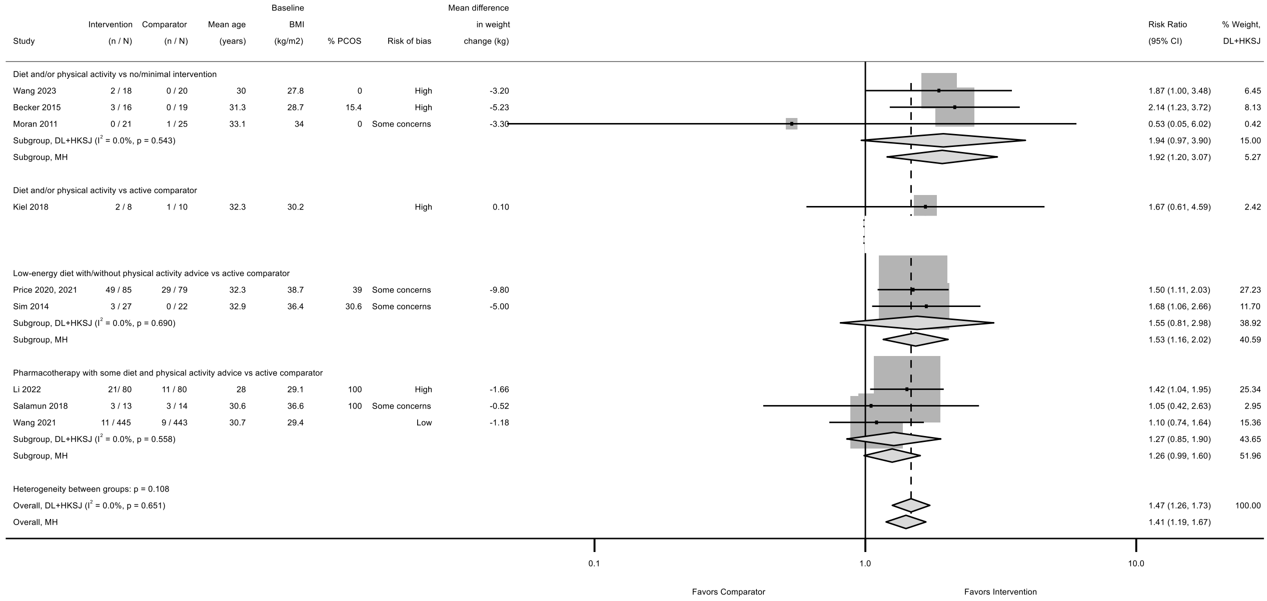

n: number of events; N: number of participants randomized; CI: confidence interval; BMI: body mass index; PCOS: polycystic ovary syndrome; CI: confidence interval; DL: DerSimonian–Laird; HKSJ: Hartung–Knapp–Sidik–Jonkman; MH: Mantel-Haenszel

In Kiel 2018, the main intervention being tested was a physical activity program, but it was also accompanied by some dietary advice.

% PCOS in the sample was unknown from Wang 2021 and Kiel 2018; Einarsson 2017 reported only the unassisted pregnancies which led to a live birth (16 in intervention, 4 in comparator), not the total number of unassisted pregnancies, and was not included in this meta-analysis; Espinos 2017 reported 0 events in both groups and was omitted from the meta-analysis; Mutsaerts 2016 did not report unassisted pregnancies for women specifically seeking IVF; Li 2022 reported weight and event data from per protocol analysis only; Price 2020, 2021 reported weight data from per protocol analysis only.

Figure S7. Unassisted pregnancy rates, intervention vs comparator groups, sorted by baseline BMI (A: all studies together; B: grouped by intervention and comparator type)

A

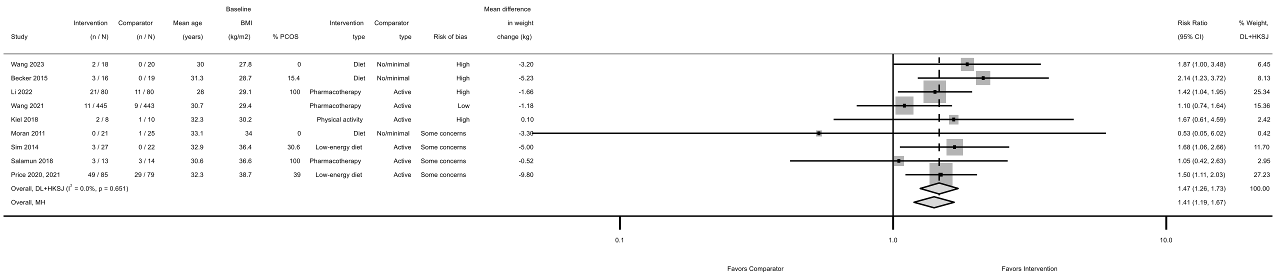

B

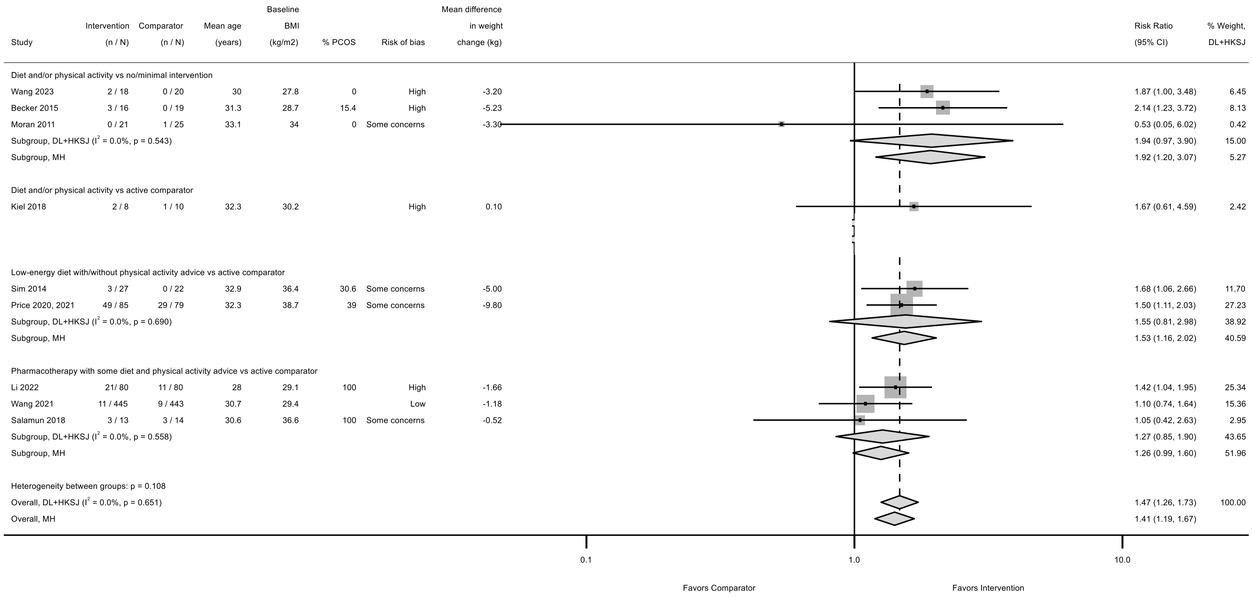

n: number of events; N: number of participants randomized; CI: confidence interval; BMI: body mass index; PCOS: polycystic ovary syndrome; CI: confidence interval; DL: DerSimonian–Laird; HKSJ: Hartung–Knapp–Sidik–Jonkman; MH: Mantel-Haenszel

In Kiel 2018, the main intervention being tested was a physical activity program, but it was also accompanied by some dietary advice.

% PCOS in the sample was unknown from Wang 2021 and Kiel 2018; Einarsson 2017 reported only the unassisted pregnancies which led to a live birth (16 in intervention, 4 in comparator), not the total number of unassisted pregnancies, and was not included in this meta-analysis; Espinos 2017 reported 0 events in both groups and was omitted from the meta-analysis; Mutsaerts 2016 did not report unassisted pregnancies for women specifically seeking IVF; Li 2022 reported weight and event data from per protocol analysis only; Price 2020, 2021 reported weight data from per protocol analysis only.

Figure S8. Unassisted pregnancy rates, intervention vs comparator groups, sorted by % PCOS in the sample (A: all studies together; B: grouped by intervention and comparator type)

A

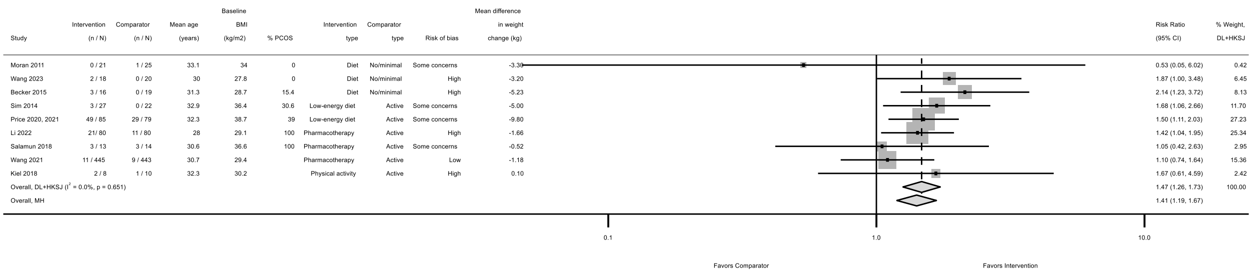

B

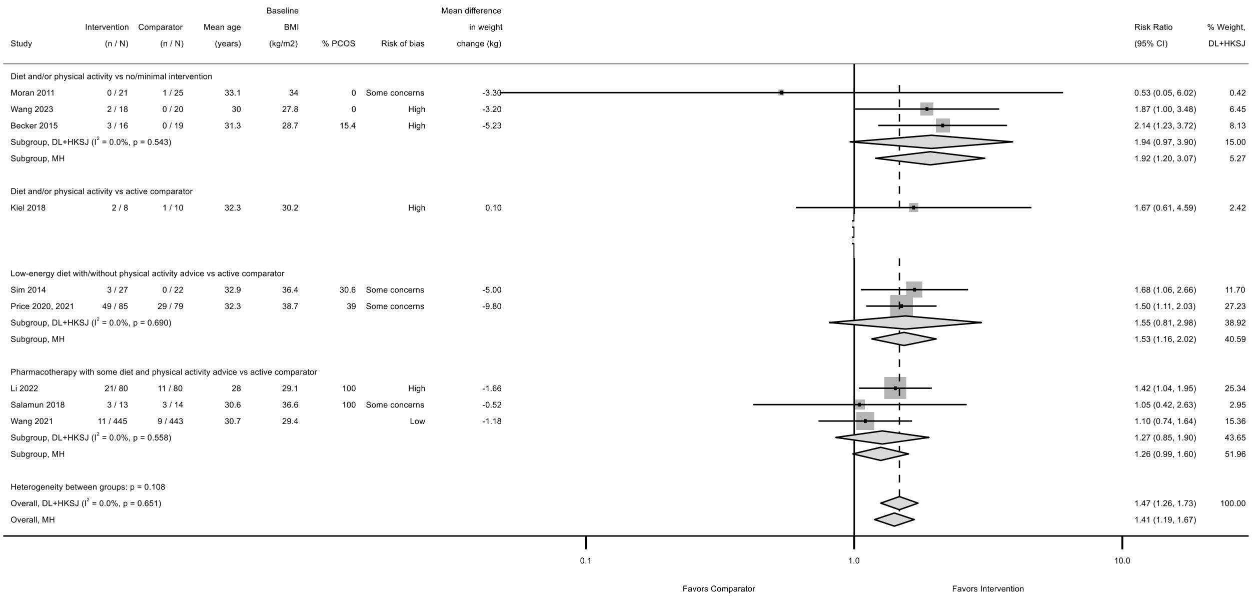

n: number of events; N: number of participants randomized; CI: confidence interval; BMI: body mass index; PCOS: polycystic ovary syndrome; CI: confidence interval; DL: DerSimonian–Laird; HKSJ: Hartung–Knapp–Sidik–Jonkman; MH: Mantel-Haenszel

In Kiel 2018, the main intervention being tested was a physical activity program, but it was also accompanied by some dietary advice.

% PCOS in the sample was unknown from Wang 2021 and Kiel 2018; Einarsson 2017 reported only the unassisted pregnancies which led to a live birth (16 in intervention, 4 in comparator), not the total number of unassisted pregnancies, and was not included in this meta-analysis; Espinos 2017 reported 0 events in both groups and was omitted from the meta-analysis; Mutsaerts 2016 did not report unassisted pregnancies for women specifically seeking IVF; Li 2022 reported weight and event data from per protocol analysis only; Price 2020, 2021 reported weight data from per protocol analysis only.

Figure S9. Unassisted pregnancy rates, intervention vs comparator groups, sorted by mean difference in weight change between groups, excluding studies at overall high risk of bias

Study	Intervention (n / N)	Comparator (n / N)	Mean age (years)	Baseline BMI (kg/m2)	% PCOS	Risk of bias	Mean difference in weight change (kg)	Risk Ratio (95% CI)	% Weight, DL+HKSJ
Diet and/or physical activity vs no/minimal intervention									
Moran 2011	0 / 21	1 / 25	33.1	34	0	Some concerns	-3.30	0.53 (0.05, 6.02)	0.74
Low-energy diet with/without physical activity advice vs active comparator									
Price 2020, 2021	49 / 85	29 / 79	32.3	38.7	39	Some concerns	-9.80	1.50 (1.11, 2.03)	47.22
Sim 2014	3 / 27	0 / 22	32.9	36.4	30.6	Some concerns	-5.00	1.68 (1.06, 2.66)	20.28
Subgroup, DL+HKSJ (I^2 = 0.0%, p = 0.690)								1.55 (0.81, 2.98)	67.50
Subgroup, MH								1.53 (1.16, 2.02)	59.89
Pharmacotherapy with some diet and physical activity advice vs active comparator									
Wang 2021	11 / 445	9 / 443	30.7	29.4		Low	-1.18	1.10 (0.74, 1.64)	26.65
Salamun 2018	3 / 13	3 / 14	30.6	36.6	100	Some concerns	-0.52	1.05 (0.42, 2.63)	5.12
Subgroup, DL+HKSJ (I^2 = 0.0%, p = 0.927)								1.09 (0.88, 1.36)	31.76
Subgroup, MH								1.09 (0.75, 1.58)	38.64
Heterogeneity between groups: p = 0.000									
Overall, DL+HKSJ (I^2 = 0.0%, p = 0.534)								1.38 (1.06, 1.79)	100.00
Overall, MH								1.34 (1.07, 1.67)	

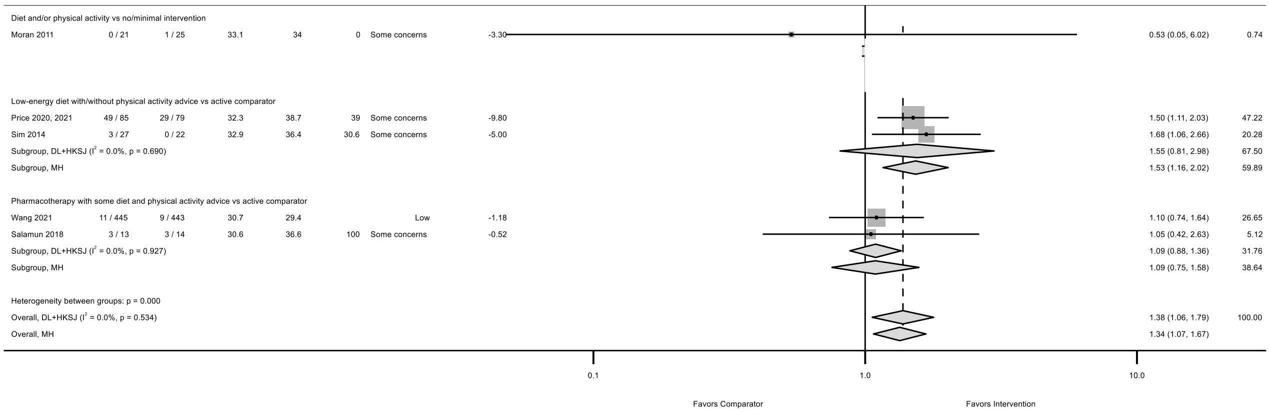

n: number of events; N: number of participants randomized; CI: confidence interval; BMI: body mass index; PCOS: polycystic ovary syndrome; CI: confidence interval; DL: DerSimonian–Laird; HKSJ: Hartung–Knapp–Sidik–Jonkman; MH: Mantel-Haenszel

% PCOS in the sample was unknown from Wang 2021; Price 2020, 2021 reported weight data from per protocol analysis only.

Figure S10. Treatment-induced pregnancy rates, intervention vs comparator groups, sorted by mean difference in weight change between groups (all studies together)

Study	Intervention (n / N)	Comparator (n / N)	Mean age (years)	Baseline BMI (kg/m2)	% PCOS	Intervention type	Comparator type	Risk of bias	Mean difference in weight change (kg)	Risk Ratio (95% CI)	% Weight, DL+HKSJ
Price 2020, 2021	5 / 85	4 / 79	32.3	38.7	39	Low-energy diet	Active	Some concerns	-9.80	1.08 (0.59, 1.97)	9.75
Sim 2014	10 / 27	3 / 22	32.9	36.4	30.6	Low-energy diet	Active	Some concerns	-5.00	1.63 (1.03, 2.57)	13.42
Moran 2011	12 / 21	8 / 25	33.1	34	0	Diet	No/minimal	Some concerns	-3.30	1.73 (0.92, 3.28)	9.08
Wang 2023	3 / 18	8 / 20	30	27.8	0	Diet	No/minimal	High	-3.20	0.49 (0.18, 1.36)	4.46
Li 2022	36 / 80	46 / 80	28	29.1	100	Pharmacotherapy	Active	High	-1.66	0.78 (0.57, 1.06)	18.22
Wang 2021	128 / 445	124 / 443	30.7	29.4		Pharmacotherapy	Active	Low	-1.18	1.02 (0.88, 1.18)	24.31
Salamun 2018	6 / 13	2 / 14	30.6	36.6	100	Pharmacotherapy	Active	Some concerns	-0.52	2.04 (1.00, 4.15)	7.82
Kiel 2018	2 / 8	3 / 10	32.3	30.2		Physical activity	Active	High	0.10	0.87 (0.25, 2.95)	3.28
Espinos 2017	12 / 21	7 / 20	32.4	34.3	41.5	Diet	No/minimal	High		1.54 (0.84, 2.84)	9.66

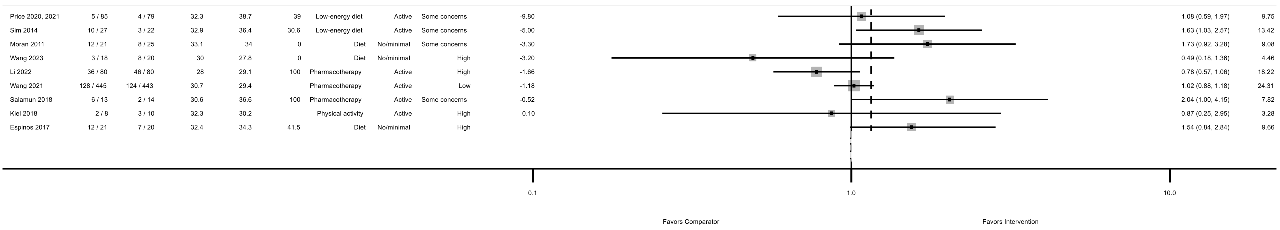

n: number of events; N: number of participants randomized; CI: confidence interval; BMI: body mass index; PCOS: polycystic ovary syndrome; CI: confidence interval; DL: DerSimonian–Laird; HKSJ: Hartung–Knapp–Sidik–Jonkman; MH: Mantel-Haenszel

All diet interventions were accompanied by physical activity advice; In Kiel 2018, the main intervention being tested was a physical activity program, but it was also accompanied by some dietary advice; All low-energy diet interventions were accompanied by some physical activity advice; All pharmacotherapy interventions were accompanied by both diet and physical activity advice.

% PCOS in the sample was unknown from Wang 2021 and Kiel 2018; Becker 2015 only reported unassisted pregnancy rates, therefore, total pregnancy, and by extent assisted pregnancy rates are not known; Espinos 2017 did not provide weight data at follow-up for the comparator, therefore it was not possible to calculate the mean difference in weight change between groups; Mutsaerts 2016 did not report unassisted pregnancies for women specifically seeking IVF, therefore assisted pregnancy rates are unknown; Li 2022 reported weight and event data from per protocol analysis only; Price 2020, 2021 reported weight data from per protocol analysis only.

Figure S11. Treatment-induced pregnancy rates, intervention vs comparator groups, sorted by mean age (A: all studies together; B: grouped by intervention and comparator type)

A

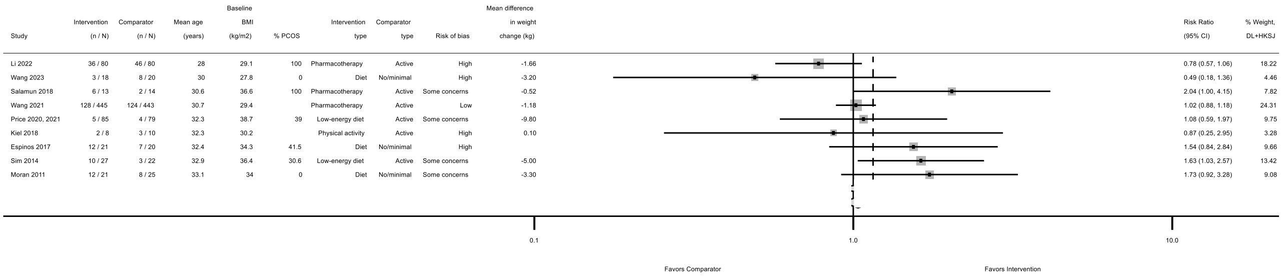

B

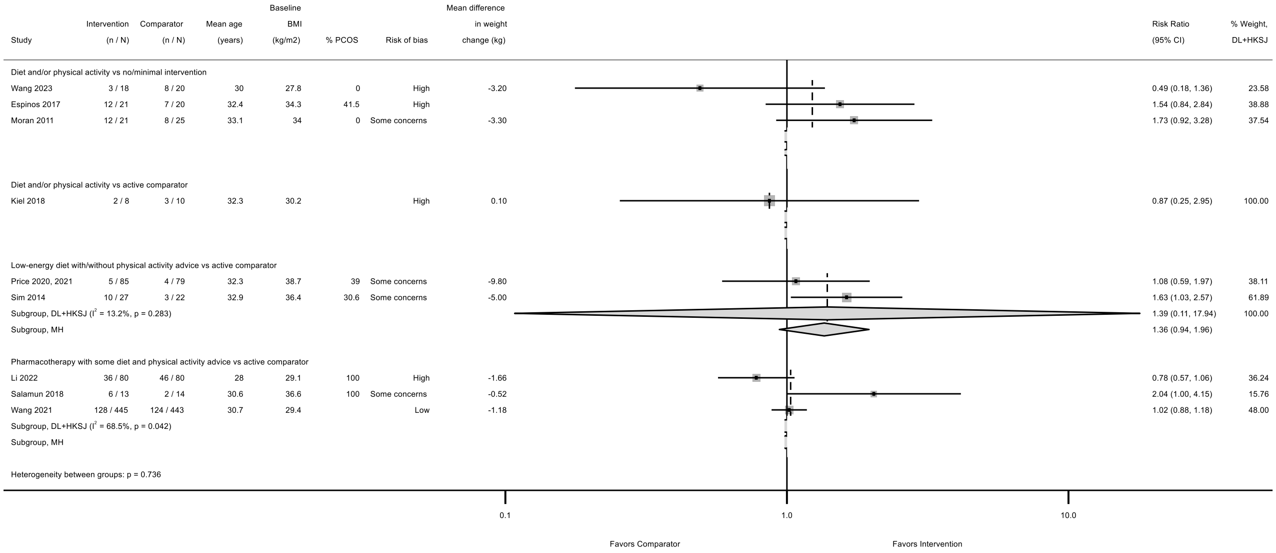

n: number of events; N: number of participants randomized; CI: confidence interval; BMI: body mass index; PCOS: polycystic ovary syndrome; CI: confidence interval; DL: DerSimonian–Laird; HKSJ: Hartung–Knapp–Sidik–Jonkman; MH: Mantel-Haenszel

In Kiel 2018, the main intervention being tested was a physical activity program, but it was also accompanied by some dietary advice.

% PCOS in the sample was unknown from Wang 2021 and Kiel 2018; Becker 2015 only reported unassisted pregnancy rates, therefore, total pregnancy, and by extent assisted pregnancy rates are not known; Espinos 2017 did not provide weight data at follow-up for the comparator, therefore it was not possible to calculate the mean difference in weight change between groups; Mutsaerts 2016 did not report unassisted pregnancies for women specifically seeking IVF, therefore assisted pregnancy rates are unknown; Li 2022 reported weight and event data from per protocol analysis only; Price 2020, 2021 reported weight data from per protocol analysis only.

Figure S12. Treatment-induced pregnancy rates, intervention vs comparator groups, sorted by baseline BMI (A: all studies together; B: grouped by intervention and comparator type)

A

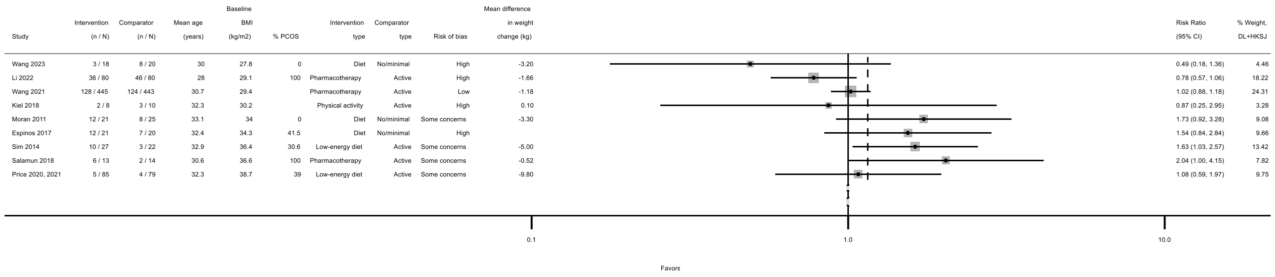

B

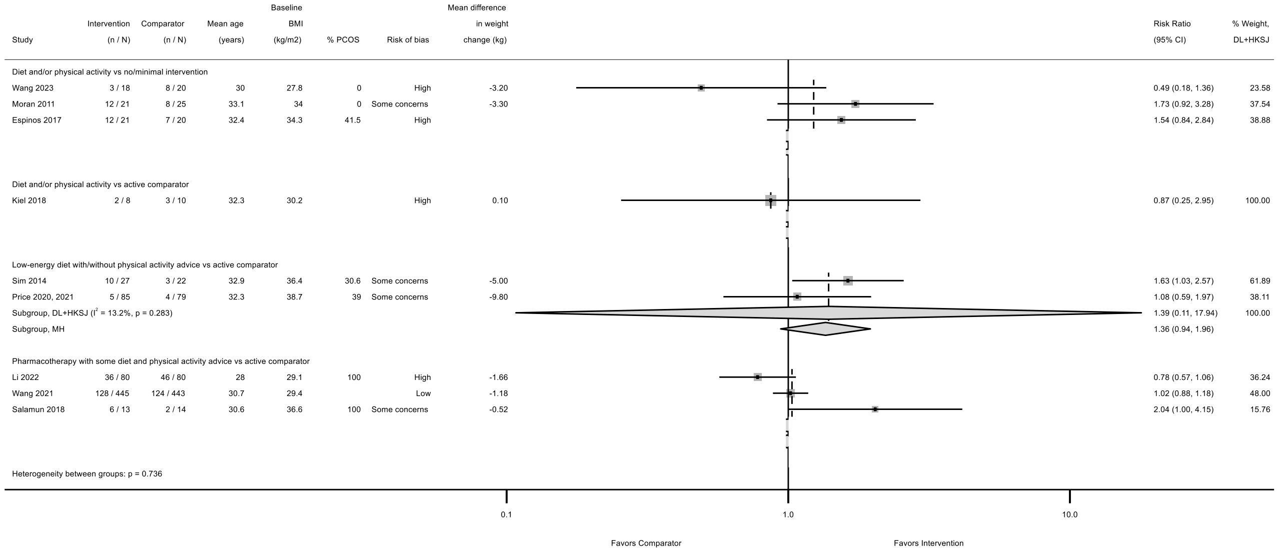

n: number of events; N: number of participants randomized; CI: confidence interval; BMI: body mass index; PCOS: polycystic ovary syndrome; CI: confidence interval; DL: DerSimonian–Laird; HKSJ: Hartung–Knapp–Sidik–Jonkman; MH: Mantel-Haenszel

In Kiel 2018, the main intervention being tested was a physical activity program, but it was also accompanied by some dietary advice.

% PCOS in the sample was unknown from Wang 2021 and Kiel 2018; Becker 2015 only reported unassisted pregnancy rates, therefore, total pregnancy, and by extent assisted pregnancy rates are not known; Espinos 2017 did not provide weight data at follow-up for the comparator, therefore it was not possible to calculate the mean difference in weight change between groups; Mutsaerts 2016 did not report unassisted pregnancies for women specifically seeking IVF, therefore assisted pregnancy rates are unknown; Li 2022 reported weight and event data from per protocol analysis only; Price 2020, 2021 reported weight data from per protocol analysis only.

Figure S13. Treatment-induced pregnancy rates, intervention vs comparator groups, sorted by % PCOS in the sample (A: all studies together; B: grouped by intervention and comparator type)

A

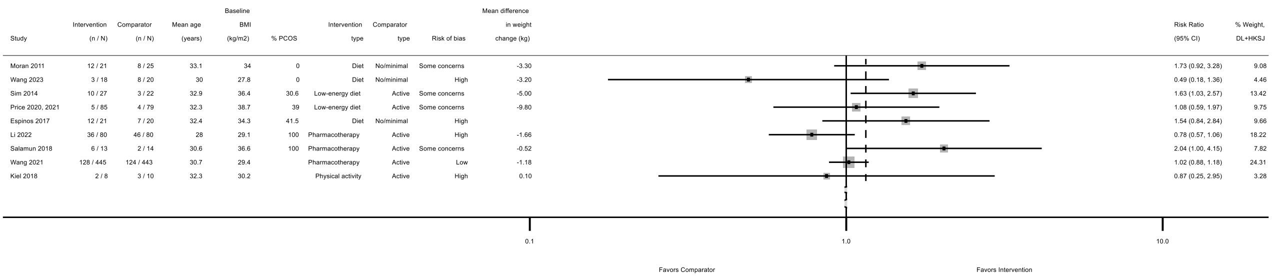

B

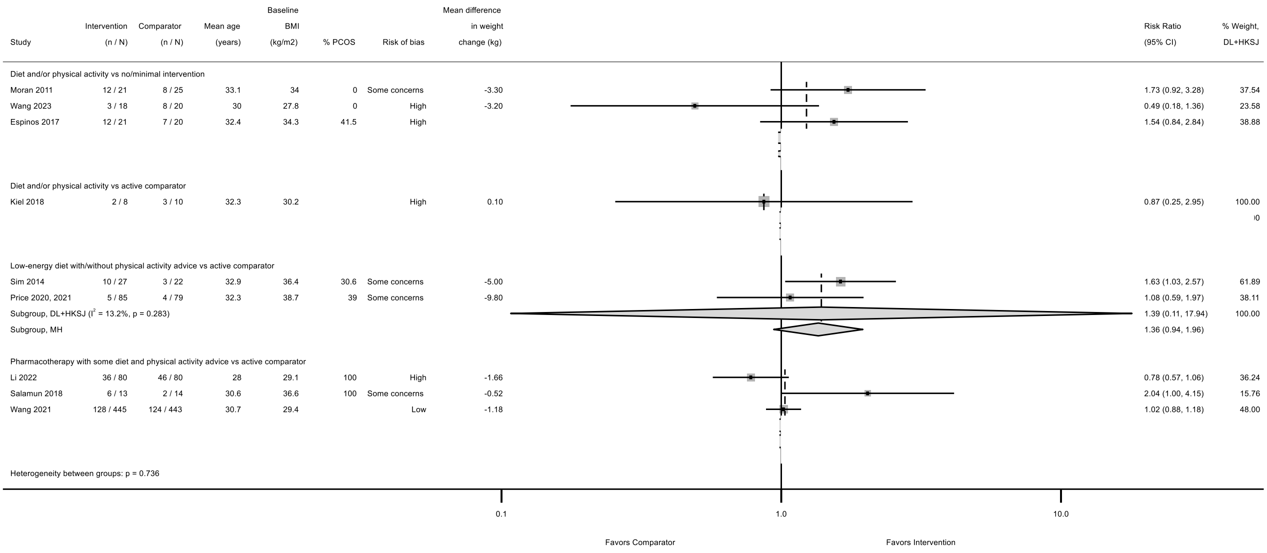

n: number of events; N: number of participants randomized; CI: confidence interval; BMI: body mass index; PCOS: polycystic ovary syndrome; CI: confidence interval; DL: DerSimonian–Laird; HKSJ: Hartung–Knapp–Sidik–Jonkman; MH: Mantel-Haenszel

In Kiel 2018, the main intervention being tested was a physical activity program, but it was also accompanied by some dietary advice.

Becker 2015 only reported unassisted pregnancy rates, therefore, total pregnancy, and by extent assisted pregnancy rates are not known; Espinos 2017 did not provide weight data at follow-up for the comparator, therefore it was not possible to calculate the mean difference in weight change between groups; Li 2022 reported weight and event data from per protocol analysis only; Price 2020, 2021 reported weight data from per protocol analysis only.

Figure S14. Treatment-induced pregnancy rates, intervention vs comparator groups, grouped by intervention and comparator type, sorted by mean difference in weight change between groups, excluding studies at overall high risk of bias

Study	Intervention (n / N)	Comparator (n / N)	Mean age (years)	Baseline BMI (kg/m2)	% PCOS	Risk of bias	Mean difference in weight change (kg)	Risk Ratio (95% CI)	% Weight, DL+HKSJ
Diet and/or physical activity vs no/minimal intervention									
Moran 2011	12 / 21	8 / 25	33.1	34	0	Some concerns	-3.30	1.73 (0.92, 3.28)	14.38
Low-energy diet with/without physical activity advice vs active comparator									
Price 2020, 2021	5 / 85	4 / 79	32.3	38.7	39	Some concerns	-9.80	1.08 (0.59, 1.97)	15.41
Sim 2014	10 / 27	3 / 22	32.9	36.4	30.6	Some concerns	-5.00	1.63 (1.03, 2.57)	20.97
Subgroup, DL+HKSJ (I^2 = 13.2%, p = 0.283)								1.39 (0.11, 17.94)	36.38
Subgroup, MH								1.36 (0.94, 1.96)	8.49
Pharmacotherapy with some diet and physical activity advice vs active comparator									
Wang 2021	128 / 445	124 / 443	30.7	29.4		Low	-1.18	1.02 (0.88, 1.18)	36.81
Salamun 2018	6 / 13	2 / 14	30.6	36.6	100	Some concerns	-0.52	2.04 (1.00, 4.15)	12.43
Heterogeneity between groups: p = 0.807									
Overall, DL+HKSJ (I^2 = 54.2%, p = 0.068)								1.33 (0.91, 1.95)	100.00
Overall, MH								1.09 (0.96, 1.25)	

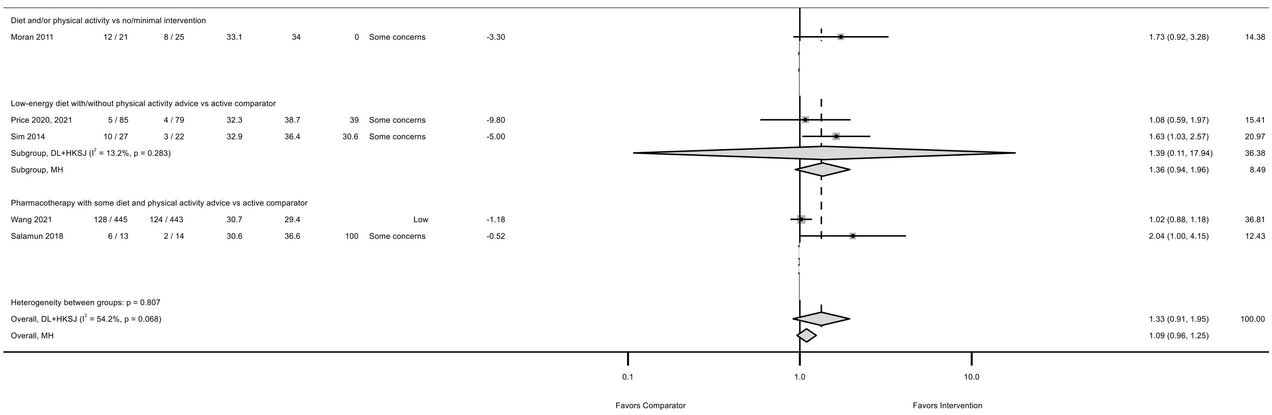

n: number of events; N: number of participants randomized; CI: confidence interval; BMI: body mass index; PCOS: polycystic ovary syndrome; CI: confidence interval; DL: DerSimonian–Laird; HKSJ: Hartung–Knapp–Sidik–Jonkman; MH: Mantel-Haenszel

% PCOS in the sample was unknown from Wang 2021; Price 2020, 2021 reported weight data from per protocol analysis only.

Figure S15. Treatment-induced pregnancy rates, intervention vs comparator groups, grouped by intervention and comparator type, using the number of treatment cycles undertaken in the denominator, and sorted by mean difference in weight change between groups

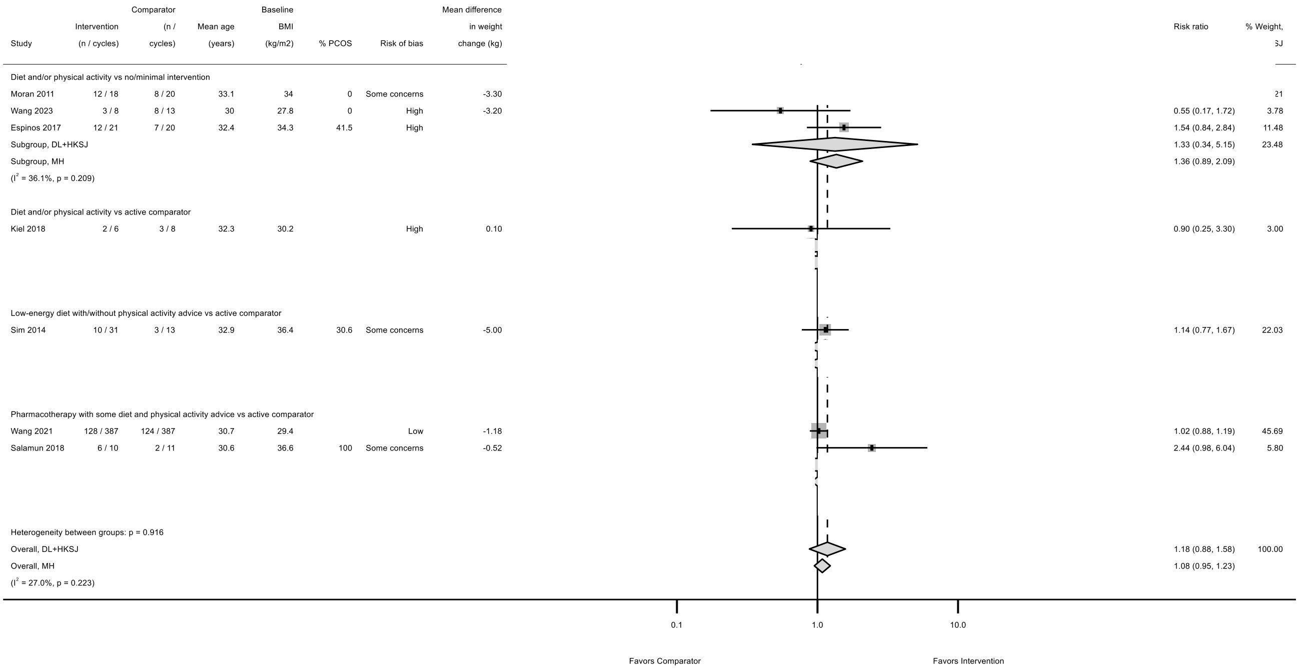

n: number of events; N: number of participants randomized; CI: confidence interval; BMI: body mass index; PCOS: polycystic ovary syndrome; CI: confidence interval; DL: DerSimonian–Laird; HKSJ: Hartung–Knapp–Sidik–Jonkman; MH: Mantel-Haenszel

In Kiel 2018, the main intervention being tested was a physical activity program, but it was also accompanied by some dietary advice.

% PCOS in the sample was unknown from Kiel 2018; Espinos 2017 did not provide weight data at follow-up for the comparator, therefore it was not possible to calculate the mean difference in weight change between groups; Li 2022, Price 2020, 2021 and Wang 2021 did not report the number of cycles undertaken, therefore were not included in this meta-analysis.

Figure S16. Live birth rates, intervention vs comparator groups, sorted by mean difference in weight change between groups (all studies together)

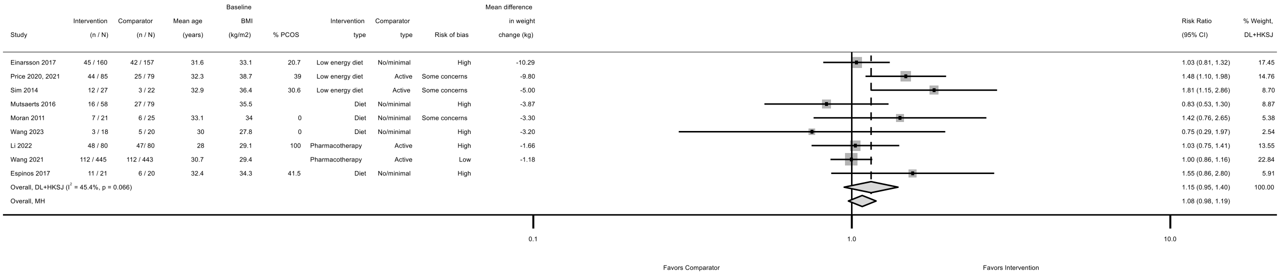

n: number of events; N: number of participants randomized; CI: confidence interval; BMI: body mass index; PCOS: polycystic ovary syndrome; CI: confidence interval; DL: DerSimonian–Laird; HKSJ: Hartung–Knapp–Sidik–Jonkman; MH: Mantel-Haenszel.

All diet interventions were accompanied by physical activity advice; In Kiel 2018, the main intervention being tested was a physical activity program, but it was also accompanied by some dietary advice; All but one (Einarsson 2017) low-energy diet interventions were accompanied by some physical activity advice; All pharmacotherapy interventions were accompanied by both diet and physical activity advice.

Mean age for women specifically seeking IVF was unknown from Mutsaerts 2016; % PCOS in the sample was unknown from Mutsaerts 2016, Wang 2021 and Kiel 2018, either because it was not reported, or it was not reported specifically for women seeking IVF; Espinos 2017 did not provide weight data at follow-up for the comparator, therefore it was not possible to calculate the mean difference in weight change between groups; Li 2022 reported events and weight data from per protocol analysis only; Price 2020, 2021 reported weight data from per protocol analysis only; We were able to obtain weight and total conception data for women specifically seeking IVF from authors of Mutsaerts 2016; Unassisted pregnancies seem to have been taken into account in live birth rates reported by Einarsson 2017, Sim 2014, Espinos 2017, Price 2020, 2021, Wang 2023, and Wang 2021; For Moran 2011 and Li 2022, it is suggestive that unassisted pregnancies were not taken into account in the reported live birth rates.

Figure S17. Live birth rates, intervention vs comparator groups, sorted by mean age (A: all studies together; B: grouped by intervention and comparator type)

A

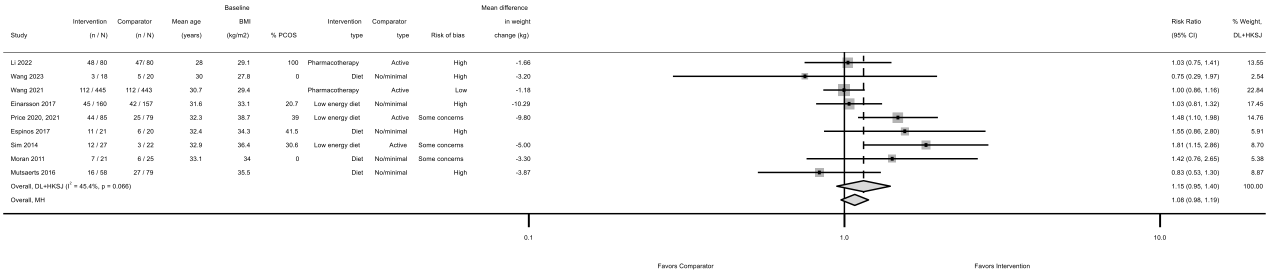

B

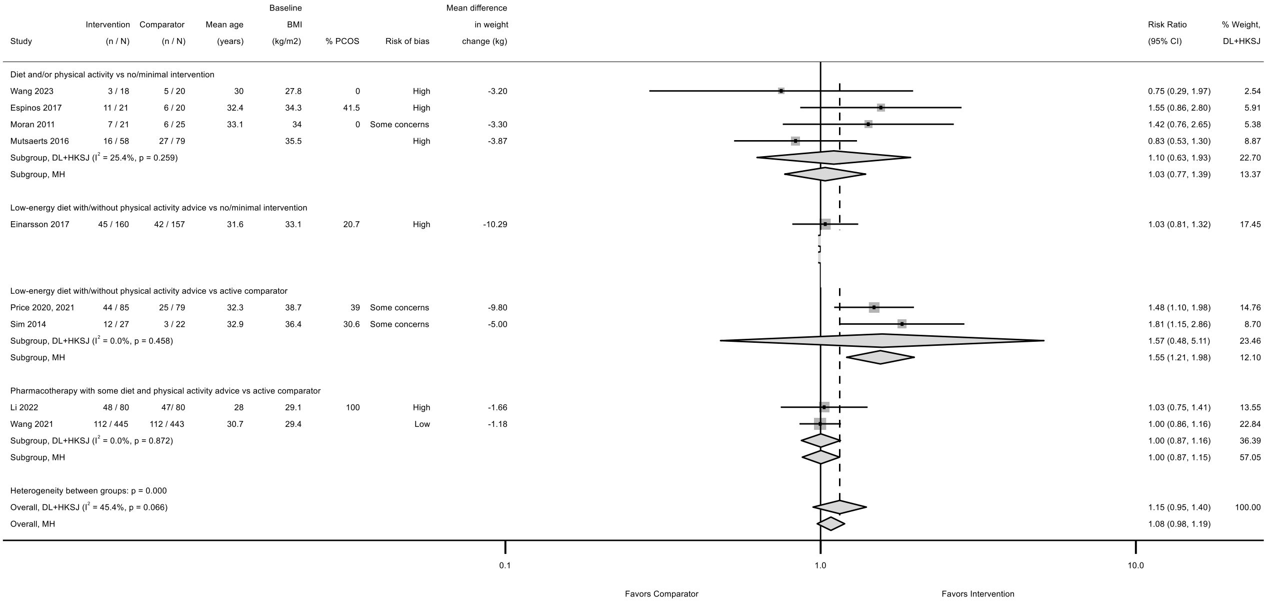

n: number of events; N: number of participants randomized; CI: confidence interval; BMI: body mass index; PCOS: polycystic ovary syndrome; CI: confidence interval; DL: DerSimonian–Laird; HKSJ: Hartung–Knapp–Sidik–Jonkman; MH: Mantel-Haenszel

Mean age for women specifically seeking IVF was unknown from Mutsaerts 2016; % PCOS in the sample was unknown from Mutsaerts 2016, Wang 2021 and Kiel 2018, either because it was not reported, or it was not reported specifically for women seeking IVF; Espinos 2017 did not provide weight data at follow-up for the comparator, therefore it was not possible to calculate the mean difference in weight change between groups; Li 2022 reported events and weight data from per protocol analysis only; Price 2020, 2021 reported weight data from per protocol analysis only; We were able to obtain weight and live birth data for women specifically seeking IVF from authors of Mutsaerts 2016; Unassisted pregnancies seem to have been taken into account in live birth rates reported by Einarsson 2017, Sim 2014, Espinos 2017, Price 2020, 2021, Wang 2023, and Wang 2021; For Moran 2011 and Li 2022, it is suggestive that unassisted pregnancies were not taken into account in the reported live birth rates.

Figure S18. Live birth rates, intervention vs comparator groups, sorted by baseline BMI (A: all studies together; B: grouped by intervention and comparator type)

A

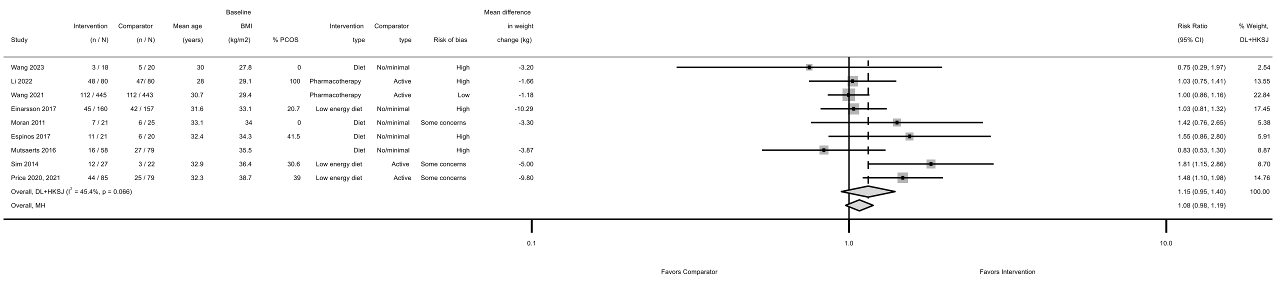

B

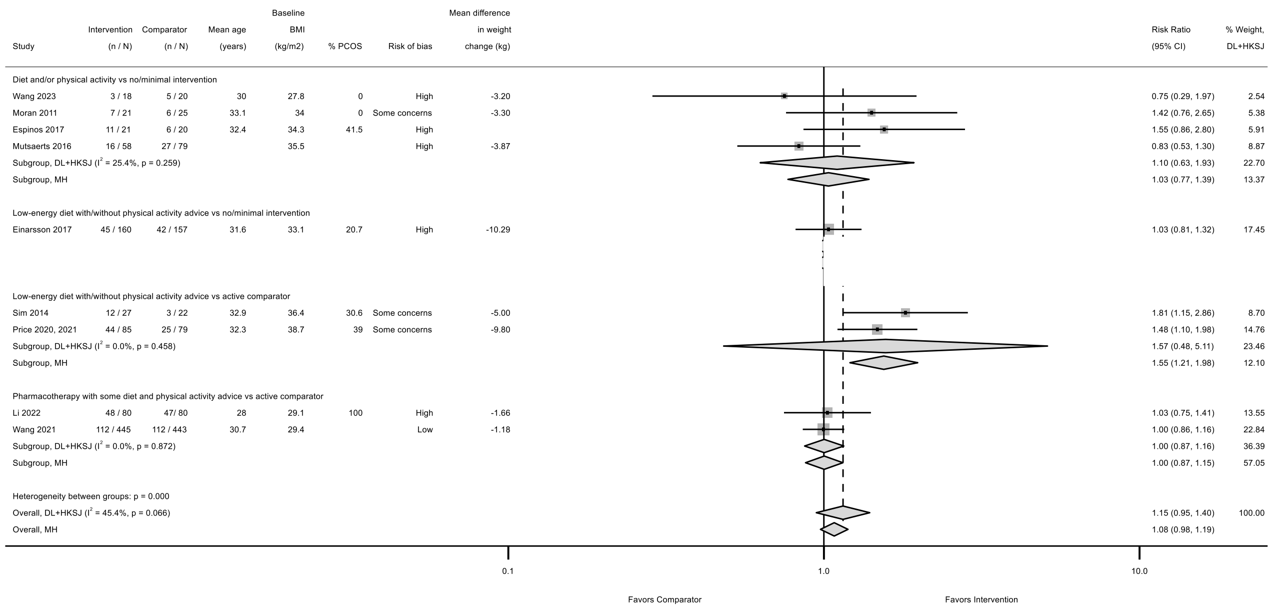

n: number of events; N: number of participants randomized; CI: confidence interval; BMI: body mass index; PCOS: polycystic ovary syndrome; CI: confidence interval; DL: DerSimonian–Laird; HKSJ: Hartung–Knapp–Sidik–Jonkman; MH: Mantel-Haenszel

Mean age for women specifically seeking IVF was unknown from Mutsaerts 2016; % PCOS in the sample was unknown from Mutsaerts 2016, Wang 2021 and Kiel 2018, either because it was not reported, or it was not reported specifically for women seeking IVF; Espinos 2017 did not provide weight data at follow-up for the comparator, therefore it was not possible to calculate the mean difference in weight change between groups; Li 2022 reported events and weight data from per protocol analysis only; Price 2020, 2021 reported weight data from per protocol analysis only; We were able to obtain weight and live birth data for women specifically seeking IVF from authors of Mutsaerts 2016; Unassisted pregnancies seem to have been taken into account in live birth rates reported by Einarsson 2017, Sim 2014, Espinos 2017, Price 2020, 2021, Wang 2023, and Wang 2021; For Moran 2011 and Li 2022, it is suggestive that unassisted pregnancies were not taken into account in the reported live birth rates.

Figure S19. Live birth rates, intervention vs comparator groups, sorted by % PCOS in the sample (A: all studies together; B: grouped by intervention and comparator type)

A

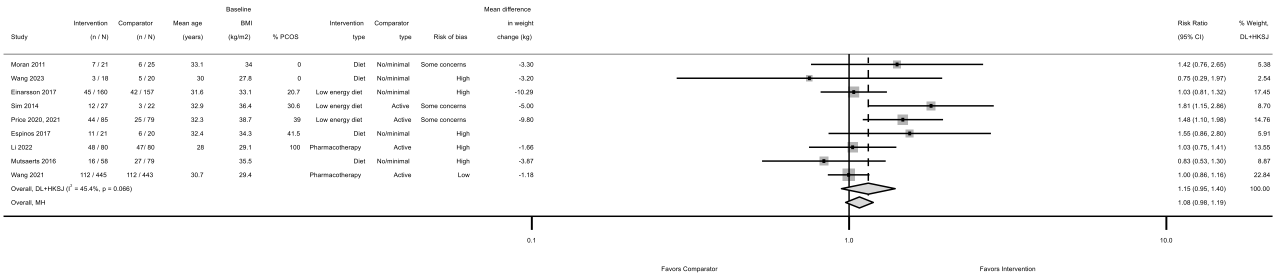

B

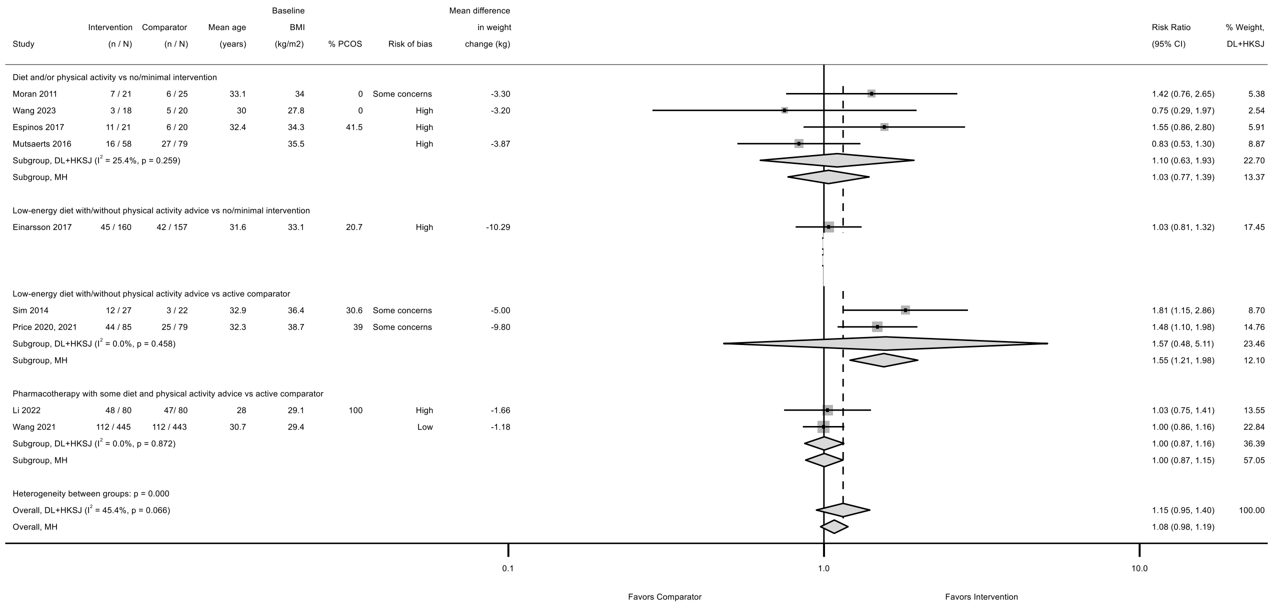

n: number of events; N: number of participants randomized; CI: confidence interval; BMI: body mass index; PCOS: polycystic ovary syndrome; CI: confidence interval; DL: DerSimonian–Laird; HKSJ: Hartung–Knapp–Sidik–Jonkman; MH: Mantel-Haenszel

Mean age for women specifically seeking IVF was unknown from Mutsaerts 2016; % PCOS in the sample was unknown from Mutsaerts 2016, Wang 2021 and Kiel 2018, either because it was not reported, or it was not reported specifically for women seeking IVF; Espinos 2017 did not provide weight data at follow-up for the comparator, therefore it was not possible to calculate the mean difference in weight change between groups; Li 2022 reported events and weight data from per protocol analysis only; Price 2020, 2021 reported weight data from per protocol analysis only; We were able to obtain weight and live birth data for women specifically seeking IVF from authors of Mutsaerts 2016; Unassisted pregnancies seem to have been taken into account in live birth rates reported by Einarsson 2017, Sim 2014, Espinos 2017, Price 2020, 2021, Wang 2023, and Wang 2021; For Moran 2011 and Li 2022, it is suggestive that unassisted pregnancies were not taken into account in the reported live birth rates.

Figure S20. Live birth rates, intervention vs comparator groups, grouped by intervention and comparator type, sorted by mean difference in weight change between groups, excluding studies at overall high risk of bias

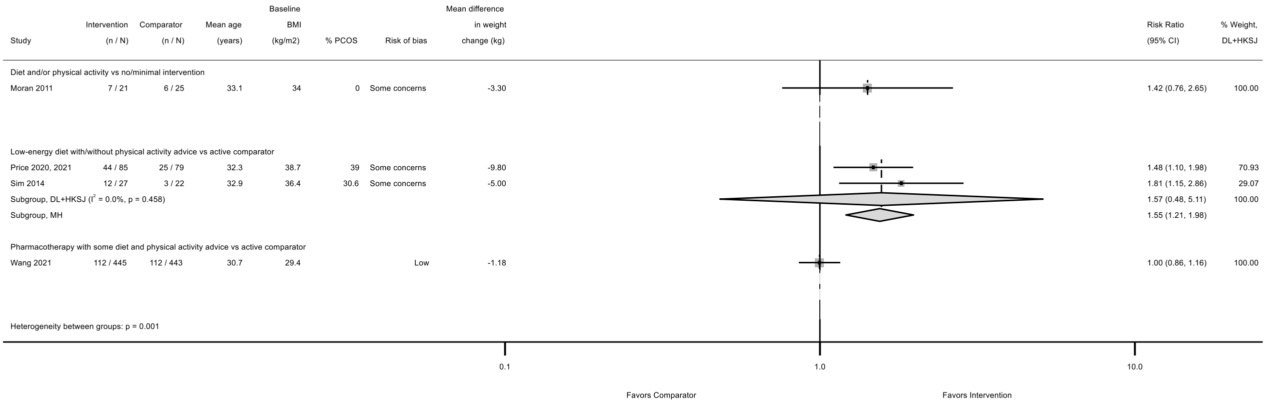

n: number of events; N: number of participants randomized; CI: confidence interval; BMI: body mass index; PCOS: polycystic ovary syndrome; CI: confidence interval; DL: DerSimonian–Laird; HKSJ: Hartung–Knapp–Sidik–Jonkman; MH: Mantel-Haenszel

% PCOS in the sample was unknown from Wang 2021; Unassisted pregnancies seem to have been taken into account in live birth rates reported by Sim 2014, Price 2020, 2021, and Wang 2021; For Moran 2011, it is suggestive that unassisted pregnancies were not taken into account in the reported live birth rates; Price 2020, 2021 reported weight data from per protocol analysis only.

Figure S21. Total pregnancy rates, intervention vs comparator groups, sorted by mean difference in weight change between groups (all studies together)

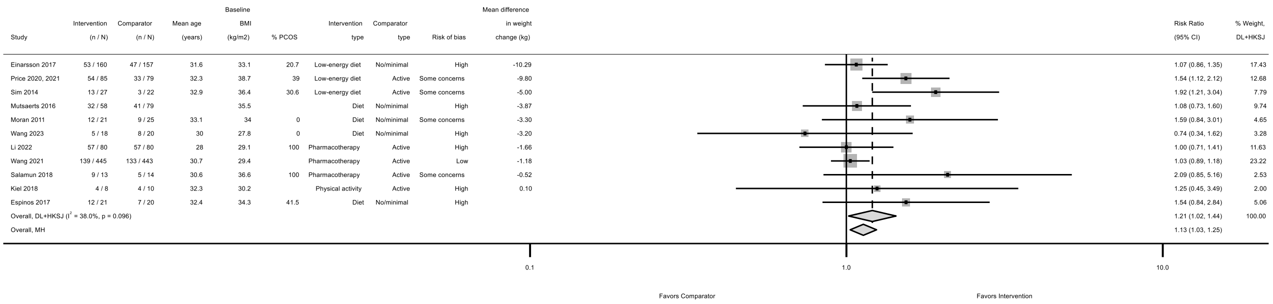

n: number of events; N: number of participants randomized; CI: confidence interval; BMI: body mass index; PCOS: polycystic ovary syndrome; CI: confidence interval; DL: DerSimonian–Laird; HKSJ: Hartung–Knapp–Sidik–Jonkman; MH: Mantel-Haenszel.

All diet interventions were accompanied by physical activity advice; In Kiel 2018, the main intervention being tested was a physical activity program, but it was also accompanied by some dietary advice; All but one (Einarsson 2017) low-energy diet interventions were accompanied by some physical activity advice; All pharmacotherapy interventions were accompanied by both diet and physical activity advice.

Mean age for women specifically seeking IVF was unknown from Mutsaerts 2016; % PCOS in the sample was unknown from Mutsaerts 2016, Wang 2021 and Kiel 2018, either because it was not reported, or it was not reported specifically for women seeking IVF; Espinos 2017 did not provide weight data at follow-up for the comparator, therefore it was not possible to calculate the mean difference in weight change between groups; Li 2022 reported events and weight data from per protocol analysis only; We were able to obtain weight and total conception data for women specifically seeking IVF from authors of Mutsaerts 2016; Becker 2015 only reported unassisted pregnancy rates, therefore, total pregnancy rates are not known; Price 2020, 2021 reported weight data from per protocol analysis only.

Figure S22. Total pregnancy rates, intervention vs comparator groups, sorted by mean age (A: all studies together; B: grouped by intervention and comparator type)

A

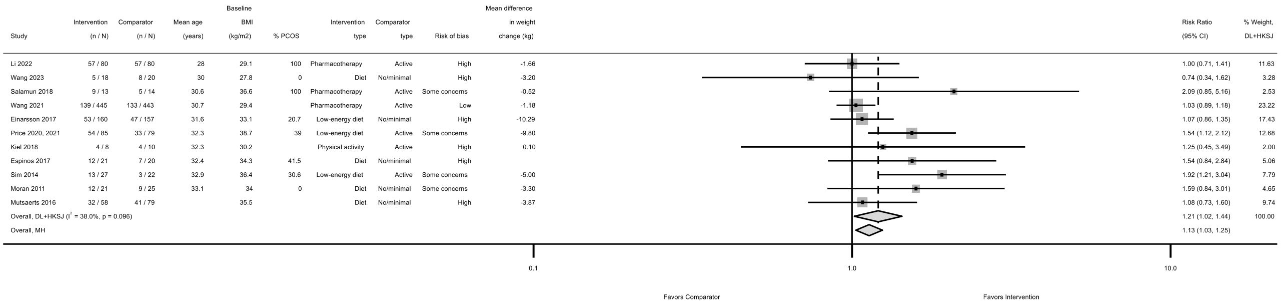

B

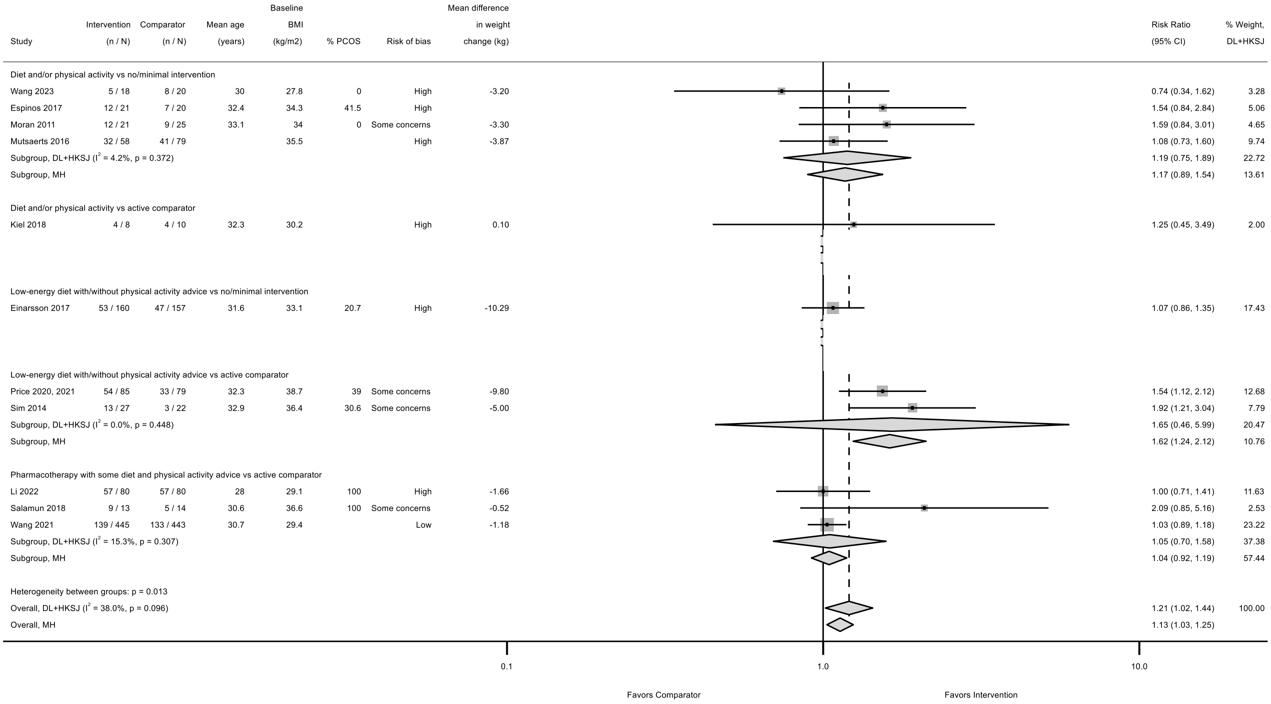

n: number of events; N: number of participants randomized; CI: confidence interval; BMI: body mass index; PCOS: polycystic ovary syndrome; CI: confidence interval; DL: DerSimonian–Laird; HKSJ: Hartung–Knapp–Sidik–Jonkman; MH: Mantel-Haenszel

In Kiel 2018, the main intervention being tested was a physical activity program, but it was also accompanied by some dietary advice.

Mean age for women specifically seeking IVF was unknown from Mutsaerts 2016; % PCOS in the sample was unknown from Mutsaerts 2016, Wang 2021 and Kiel 2018, either because it was not reported, or it was not reported specifically for women seeking IVF; Espinos 2017 did not provide weight data at follow-up for the comparator, therefore it was not possible to calculate the mean difference in weight change between groups; Becker 2015 only reported unassisted pregnancy rates, therefore, total pregnancy rates are not known; Li 2022 reported weight and event data from per protocol analysis only; Price 2020, 2021 reported weight data from per protocol analysis only; We were able to obtain weight and total pregnancy data for women specifically seeking IVF from authors of Mutsaerts 2016.

Figure S23. Total pregnancy rates, intervention vs comparator groups, sorted by baseline BMI (A: all studies together; B: grouped by intervention and comparator type)

A

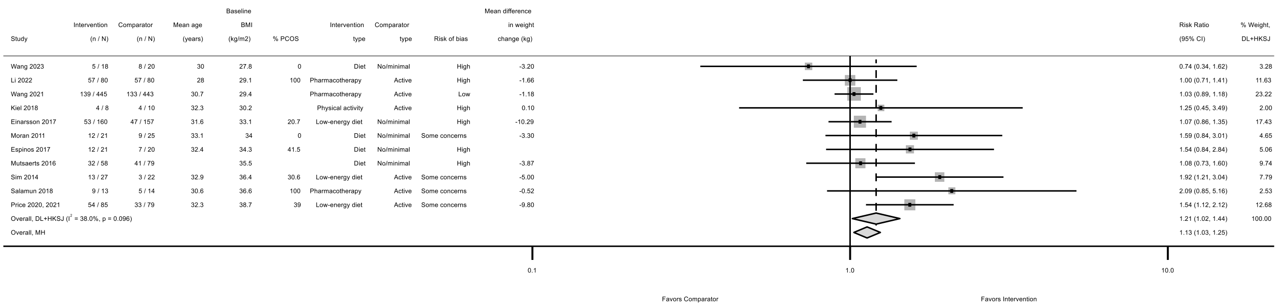

B

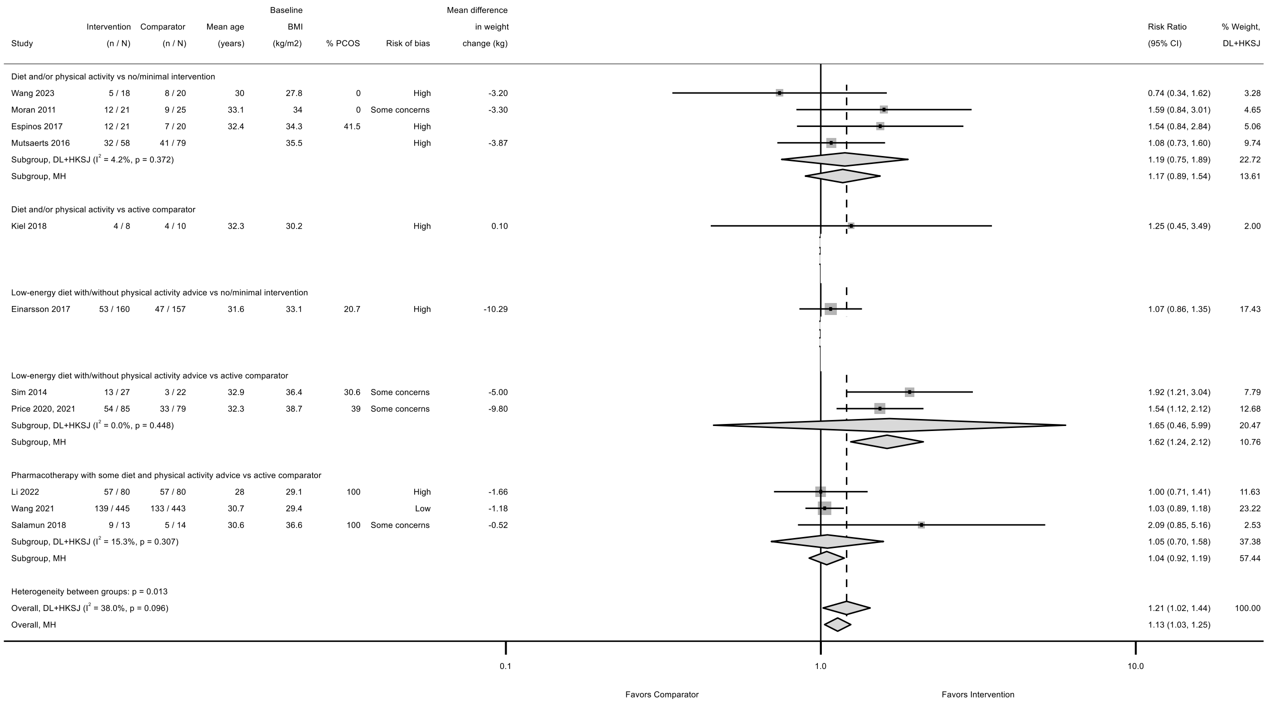

n: number of events; N: number of participants randomized; CI: confidence interval; BMI: body mass index; PCOS: polycystic ovary syndrome; CI: confidence interval; DL: DerSimonian–Laird; HKSJ: Hartung–Knapp–Sidik–Jonkman; MH: Mantel-Haenszel

In Kiel 2018, the main intervention being tested was a physical activity program, but it was also accompanied by some dietary advice.

Mean age for women specifically seeking IVF was unknown from Mutsaerts 2016; % PCOS in the sample was unknown from Mutsaerts 2016, Wang 2021 and Kiel 2018, either because it was not reported, or it was not reported specifically for women seeking IVF; Espinos 2017 did not provide weight data at follow-up for the comparator, therefore it was not possible to calculate the mean difference in weight change between groups; Becker 2015 only reported unassisted pregnancy rates, therefore, total pregnancy rates are not known; Li 2022 reported weight and event data from per protocol analysis only; Price 2020, 2021 reported weight data from per protocol analysis only; We were able to obtain weight and total pregnancy data for women specifically seeking IVF from authors of Mutsaerts 2016.

Figure S24. Total pregnancy rates, intervention vs comparator groups, sorted by % PCOS in the sample (A: all studies together; B: grouped by intervention and comparator type)

A

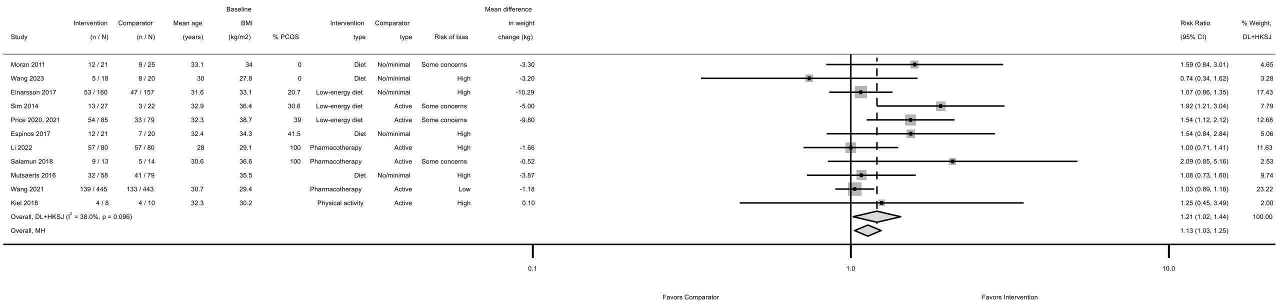

B

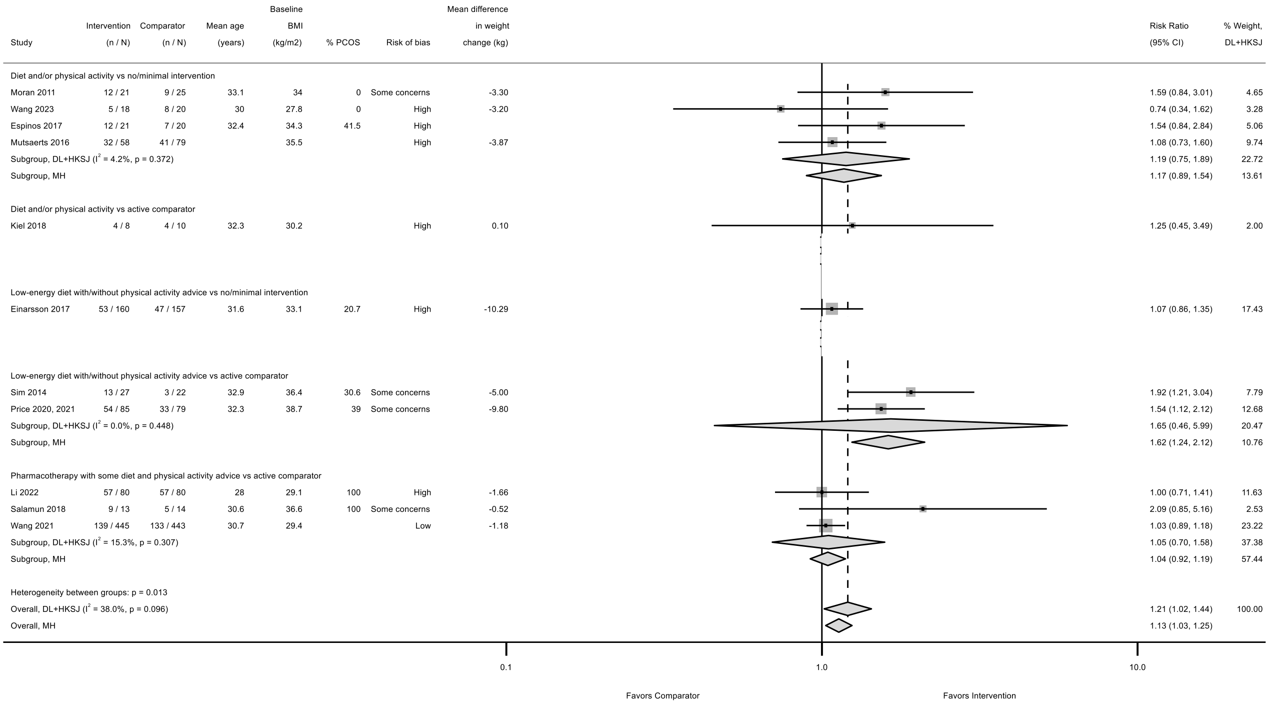

n: number of events; N: number of participants randomized; CI: confidence interval; BMI: body mass index; PCOS: polycystic ovary syndrome; CI: confidence interval; DL: DerSimonian–Laird; HKSJ: Hartung–Knapp–Sidik–Jonkman; MH: Mantel-Haenszel

In Kiel 2018, the main intervention being tested was a physical activity program, but it was also accompanied by some dietary advice.

Mean age for women specifically seeking IVF was unknown from Mutsaerts 2016; % PCOS in the sample was unknown from Mutsaerts 2016, Wang 2021 and Kiel 2018, either because it was not reported, or it was not reported specifically for women seeking IVF; Espinos 2017 did not provide weight data at follow-up for the comparator, therefore it was not possible to calculate the mean difference in weight change between groups; Becker 2015 only reported unassisted pregnancy rates, therefore, total pregnancy rates are not known; Li 2022 reported weight and event data from per protocol analysis only; Price 2020, 2021 reported weight data from per protocol analysis only; We were able to obtain weight and total pregnancy data for women specifically seeking IVF from authors of Mutsaerts 2016.

Figure S25. Total pregnancy rates, intervention vs comparator groups, grouped by intervention and comparator type, sorted by mean difference in weight change between groups, excluding studies at overall high risk of bias

Study	Intervention (n / N)	Comparator (n / N)	Mean age (years)	Baseline BMI (kg/m2)	% PCOS	Risk of bias	Mean difference in weight change (kg)	Risk Ratio (95% CI)	% Weight, DL+HKSJ
Diet and/or physical activity vs no/minimal intervention									
Moran 2011	12 / 21	9 / 25	33.1	34	0	Some concerns	-3.30	1.59 (0.84, 3.01)	14.26
Low-energy diet with/without physical activity advice vs active comparator									
Sim 2014	13 / 27	3 / 22	32.9	36.4	30.6	Some concerns	-5.00	1.92 (1.21, 3.04)	19.73
Price 2020, 2021	54 / 85	33 / 79	32.3	38.7	39	Some concerns	-9.80	1.54 (1.12, 2.12)	25.27
Subgroup, DL+HKSJ (I^2 = 0.0%, p = 0.448)								1.65 (0.46, 5.99)	45.00
Subgroup, MH								1.62 (1.24, 2.12)	17.38
Pharmacotherapy with some diet and physical activity advice vs active comparator									
Salamun 2018	9 / 13	5 / 14	30.6	36.6	100	Some concerns	-0.52	2.09 (0.85, 5.16)	9.06
Wang 2021	139 / 445	133 / 443	30.7	29.4		Low	-1.18	1.03 (0.89, 1.18)	31.69
Heterogeneity between groups: p = 0.732									
Overall, DL+HKSJ (I^2 = 69.1%, p = 0.012)								1.46 (1.02, 2.09)	100.00
Overall, MH								1.17 (1.04, 1.32)	

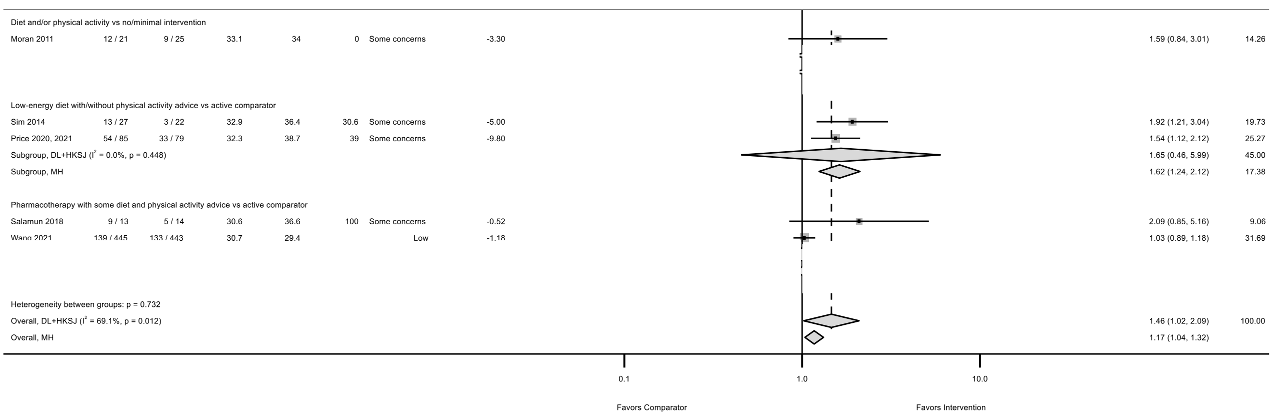

n: number of events; N: number of participants randomized; CI: confidence interval; BMI: body mass index; PCOS: polycystic ovary syndrome; CI: confidence interval; DL: DerSimonian–Laird; HKSJ: Hartung–Knapp–Sidik–Jonkman; MH: Mantel-Haenszel

% PCOS in the sample was unknown from Wang 2021; Price 2020, 2021 reported weight data from per protocol analysis only.

Figure S26. Pregnancy loss rates, intervention vs comparator groups, sorted by mean difference in weight change between groups (all studies together)

Study	Intervention (n / N)	Comparator (n / N)	Mean age (years)	Baseline BMI (kg/m2)	% PCOS	Intervention type	Comparator type	Risk of bias	Mean difference in weight change (kg)	Risk Ratio (95% CI)	% Weight, DL+HKSJ
Einarsson 2017	21 / 160	14 / 157	31.6	33.1	20.7	Low-energy diet	No/minimal	High	-10.29	1.22 (0.91, 1.64)	20.26
Price 2020, 2021	13 / 85	12 / 79	32.3	38.7	39	Low-energy diet	Active	Some concerns	-9.80	1.00 (0.67, 1.51)	10.54
Sim 2014	8 / 27	6 / 22	32.9	36.4	30.6	Low-energy diet	Active	Some concerns	-5.00	1.05 (0.61, 1.82)	5.92
Mutsaerts 2016	9 / 58	12 / 79		35.5		Diet	No/minimal	High	-3.87	1.01 (0.59, 1.74)	6.11
Wang 2023	2 / 18	3 / 20	30	27.8	0	Diet	No/minimal	High	-3.20	0.83 (0.27, 2.55)	1.38
Li 2022	19 / 80	19 / 80	28	29.1	100	Pharmacotherapy	Active	High	-1.66	1.00 (0.69, 1.44)	13.33
Wang 2021	50 / 445	48 / 443	30.7	29.4		Pharmacotherapy	Active	Low	-1.18	1.02 (0.83, 1.25)	41.57
Espinos 2017	1 / 21	1 / 20	32.4	34.3	41.5	Diet	No/minimal	High		0.97 (0.24, 4.03)	0.88
Overall, DL+HKSJ (I^2 = 0.0%, p = 0.987)										1.05 (0.98, 1.13)	100.00
Overall, MH										1.04 (0.91, 1.19)	

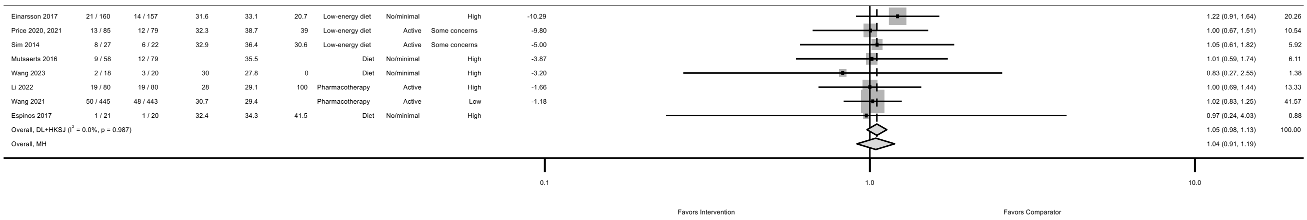

n: number of events; N: number of participants randomized; CI: confidence interval; BMI: body mass index; PCOS: polycystic ovary syndrome; CI: confidence interval; DL: DerSimonian–Laird; HKSJ: Hartung–Knapp–Sidik–Jonkman; MH: Mantel-Haenszel

All diet interventions were accompanied by physical activity advice; All but one (Einarsson 2017) low-energy diet interventions were accompanied by some physical activity advice; All pharmacotherapy interventions were accompanied by both diet and physical activity advice.

Mean age for women specifically seeking IVF was unknown from Mutsaerts 2016; % PCOS in the sample was unknown from Mutsaerts 2016 and Wang 2021, either because it was not reported, or it was not reported specifically for women seeking IVF; Espinos 2017 did not provide weight data at follow-up for the comparator, therefore it was not possible to calculate the mean difference in weight change between groups; Li 2022 reported weight and event data from per protocol analysis only; Price 2020, 2021 reported weight data from per protocol analysis only; We were able to obtain weight and pregnancy loss data for women specifically seeking IVF from authors of Mutsaerts 2016. Unassisted pregnancies seem to have been taken into account in pregnancy loss rates reported by Einarsson 2017, Price 2020, 2021, Sim 2014, Espinos 2017, Wang 2023, and Wang 2021; For Li 2022, it is suggestive that unassisted pregnancies were not taken into account in the reported pregnancy loss rates.

Figure S27. Pregnancy loss rates, intervention vs comparator groups, sorted by mean age (A: all studies together; B: grouped by intervention and comparator type)

A

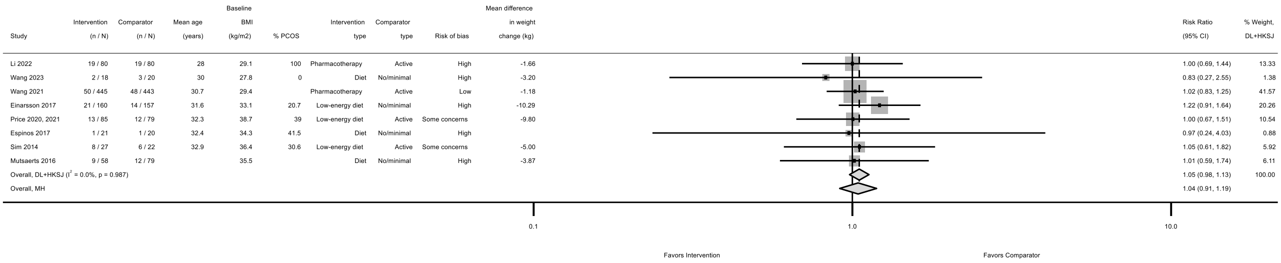

B

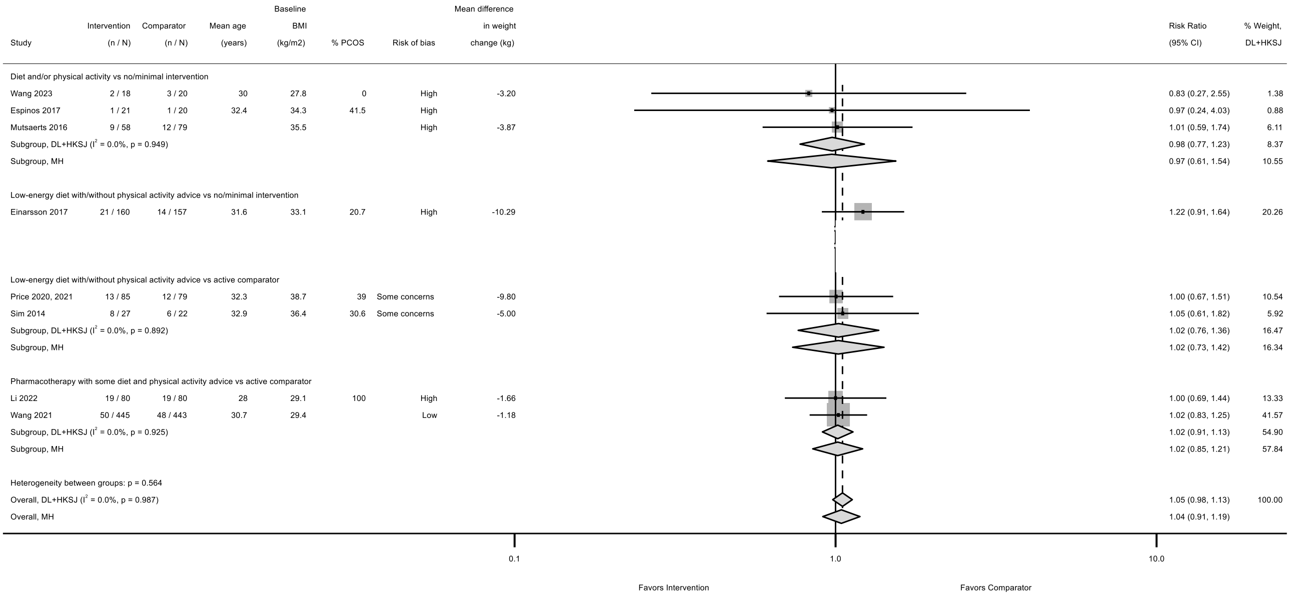

n: number of events; N: number of participants randomized; CI: confidence interval; BMI: body mass index; PCOS: polycystic ovary syndrome; CI: confidence interval; DL: DerSimonian–Laird; HKSJ: Hartung–Knapp–Sidik–Jonkman; MH: Mantel-Haenszel

Mean age for women specifically seeking IVF was unknown from Mutsaerts 2016; % PCOS in the sample was unknown from Mutsaerts 2016 and Wang 2021, either because it was not reported, or it was not reported specifically for women seeking IVF; Espinos 2017 did not provide weight data at follow-up for the comparator, therefore it was not possible to calculate the mean difference in weight change between groups; Li 2022 reported weight and event data from per protocol analysis only; Price 2020, 2021 reported weight data from per protocol analysis only; We were able to obtain weight and pregnancy loss data for women specifically seeking IVF from authors of Mutsaerts 2016. Unassisted pregnancies seem to have been taken into account in pregnancy loss rates reported by Einarsson 2017, Prcie 2020, 2021, Sim 2014, Espinos 2017, Wang 2023, and Wang 2021; For Li 2022, it is suggestive that unassisted pregnancies were not taken into account in the reported pregnancy loss rates.

Figure S28. Pregnancy loss rates, intervention vs comparator groups, sorted by baseline BMI (A: all studies together; B: grouped by intervention and comparator type)

A

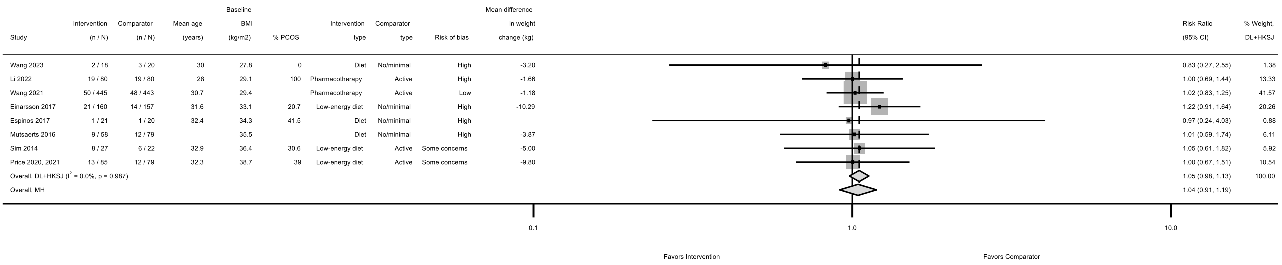

B

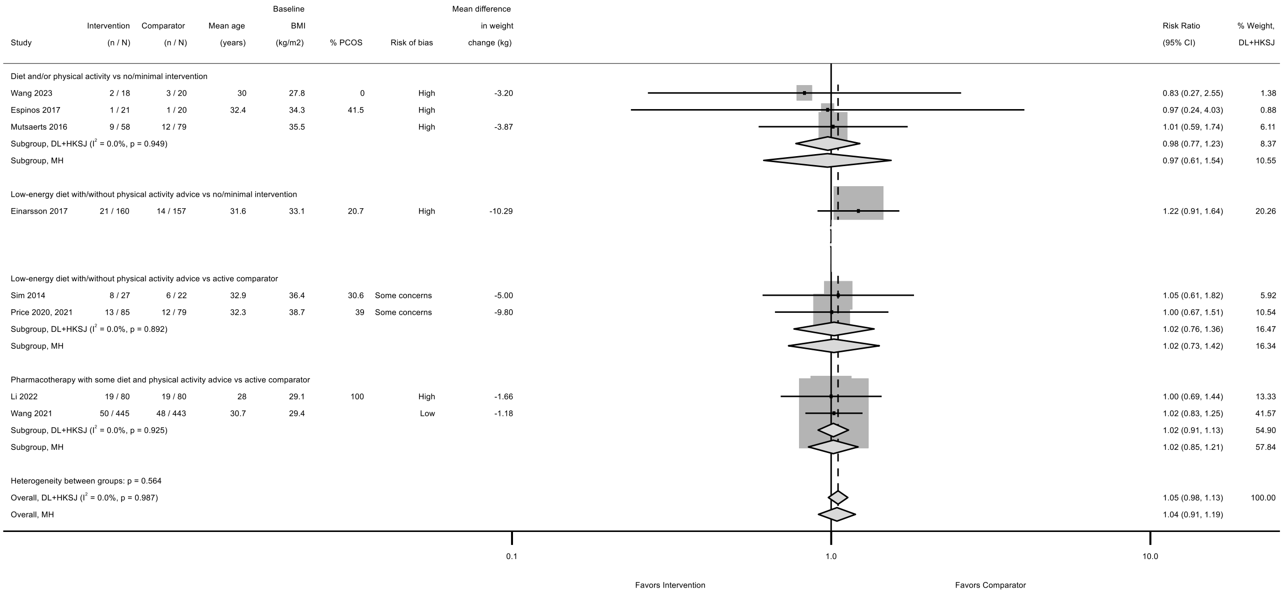

n: number of events; N: number of participants randomized; CI: confidence interval; BMI: body mass index; PCOS: polycystic ovary syndrome; CI: confidence interval; DL: DerSimonian–Laird; HKSJ: Hartung–Knapp–Sidik–Jonkman; MH: Mantel-Haenszel

Mean age for women specifically seeking IVF was unknown from Mutsaerts 2016; % PCOS in the sample was unknown from Mutsaerts 2016 and Wang 2021, either because it was not reported, or it was not reported specifically for women seeking IVF; Espinos 2017 did not provide weight data at follow-up for the comparator, therefore it was not possible to calculate the mean difference in weight change between groups; Li 2022 reported weight and event data from per protocol analysis only; Price 2020, 2021 reported weight data from per protocol analysis only; We were able to obtain weight and pregnancy loss data for women specifically seeking IVF from authors of Mutsaerts 2016. Unassisted pregnancies seem to have been taken into account in pregnancy loss rates reported by Einarsson 2017, Price 2020, 2021, Sim 2014, Espinos 2017, Wang 2023, and Wang 2021; For Li 2022, it is suggestive that unassisted pregnancies were not taken into account in the reported pregnancy loss rates.

Figure S29. Pregnancy loss rates, intervention vs comparator groups, sorted by % PCOS in the sample (A: all studies together; B: grouped by intervention and comparator type)

A

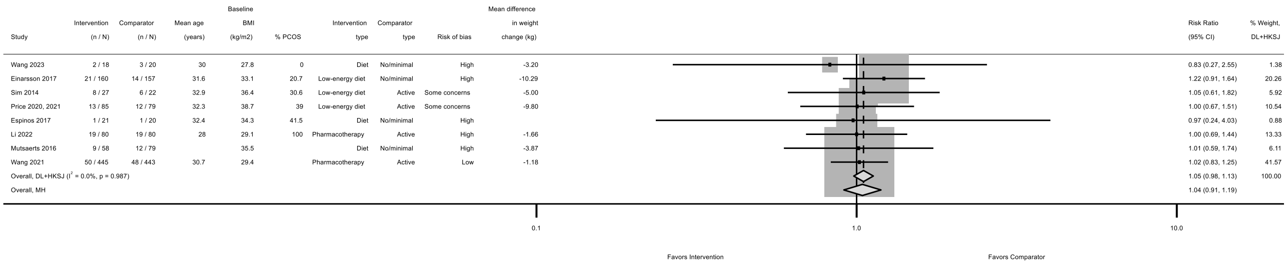

B

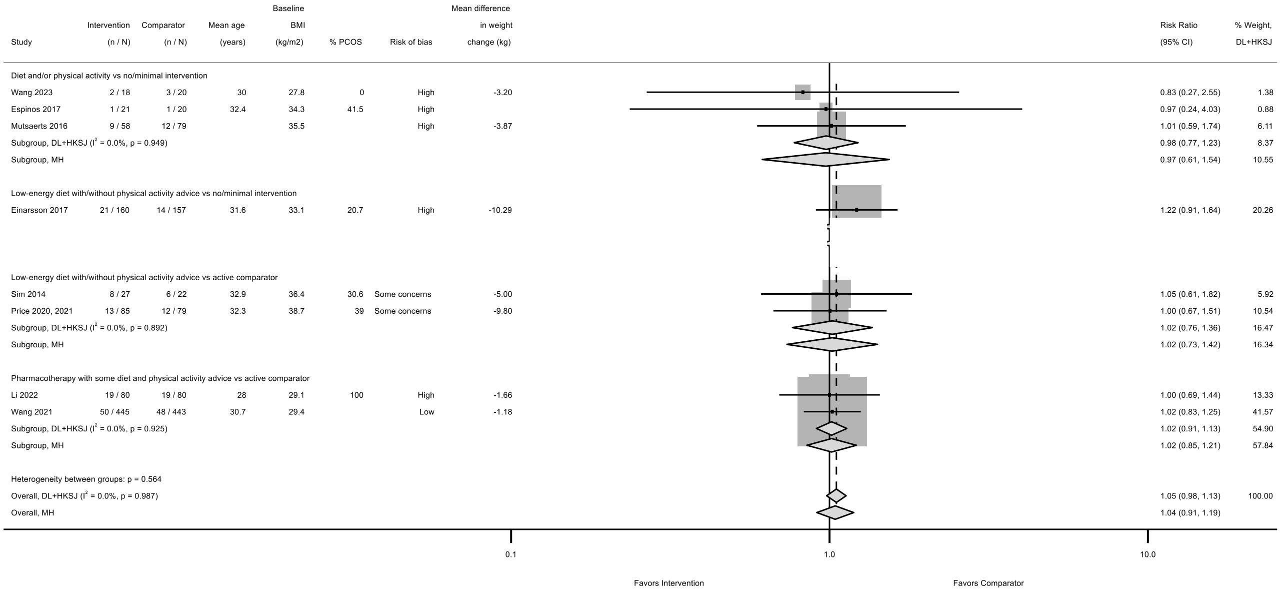

n: number of events; N: number of participants randomized; CI: confidence interval; BMI: body mass index; PCOS: polycystic ovary syndrome; CI: confidence interval; DL: DerSimonian–Laird; HKSJ: Hartung–Knapp–Sidik–Jonkman; MH: Mantel-Haenszel

Mean age for women specifically seeking IVF was unknown from Mutsaerts 2016; % PCOS in the sample was unknown from Mutsaerts 2016 and Wang 2021, either because it was not reported, or it was not reported specifically for women seeking IVF; Espinos 2017 did not provide weight data at follow-up for the comparator, therefore it was not possible to calculate the mean difference in weight change between groups; Li 2022 reported weight and event data from per protocol analysis only; Price 2020, 2021 reported weight data from per protocol analysis only; Unassisted pregnancies seem to have been taken into account in pregnancy loss rates reported by Einarsson 2017, Price 2020, 2021, Sim 2014, Espinos 2017, and Wang 2023,; For Li 2022, it is suggestive that unassisted pregnancies were not taken into account in the reported pregnancy loss rates.

Figure S30. Pregnancy loss rates, intervention vs comparator groups, grouped by intervention and comparator type, sorted by mean difference in weight change between groups, excluding studies at overall high RoB

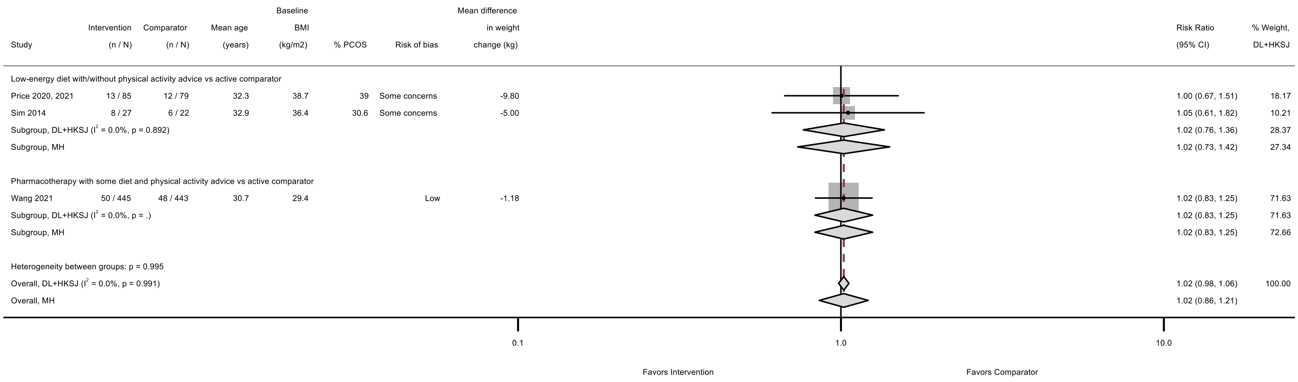

n: number of events; N: number of participants randomized; CI: confidence interval; BMI: body mass index; PCOS: polycystic ovary syndrome; CI: confidence interval; DL: DerSimonian–Laird; HKSJ: Hartung–Knapp–Sidik–Jonkman; MH: Mantel-Haenszel

% PCOS in the sample was unknown from Wang 2021; Unassisted pregnancies seem to have been taken into account in pregnancy loss rates reported by both Price 2020, 2021, Sim 2014 and Wang 2021; Price 2020, 2021 reported weight data from per protocol analysis only.

Other reproductive outcomes

Time to pregnancy was only reported by one study of low-energy diet vs active comparator, with the intervention group achieving pregnancy sooner than the comparator (34, 35). Two studies reported delivery method, with the intervention group being less likely to require caesarean section in one study of diet and physical activity intervention where the comparator commenced IVF immediately (29), but more likely in the other study where pharmacotherapy was compared to an active comparator (36). Three studies reported minimal rates of congenital abnormalities and neonatal deaths, with no difference between groups (29, 34, 35, 37).

Figure S31. Meta-regressions of weight change between intervention and comparator groups, and reproductive outcomes, by comparator type

A1. Unassisted pregnancies – no/minimal comparator

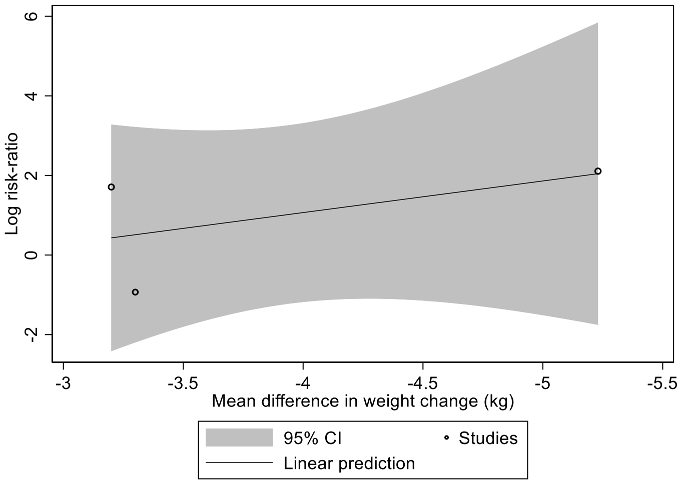

A2. Unassisted pregnancies – active comparator

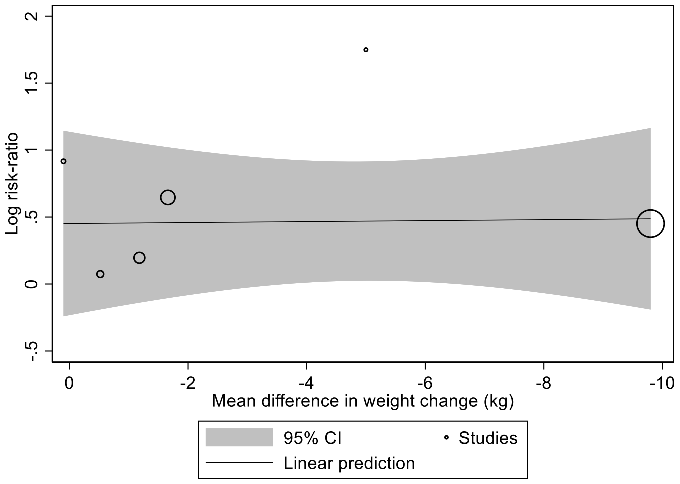

B1. IVF-induced pregnancies – no/minimal comparator

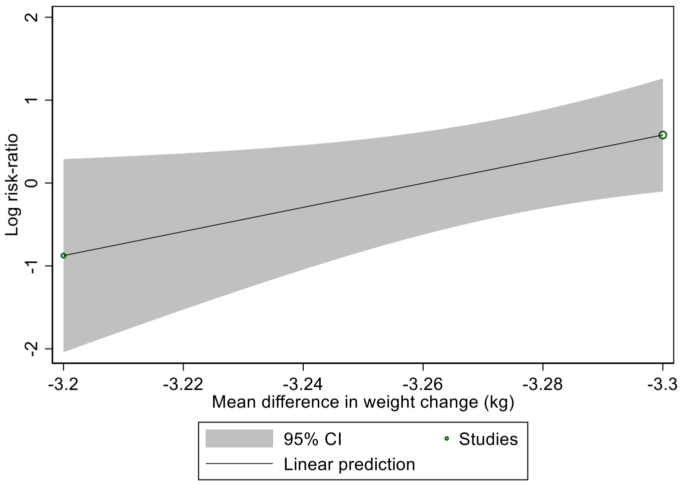

B2. IVF-induced pregnancies – active comparator

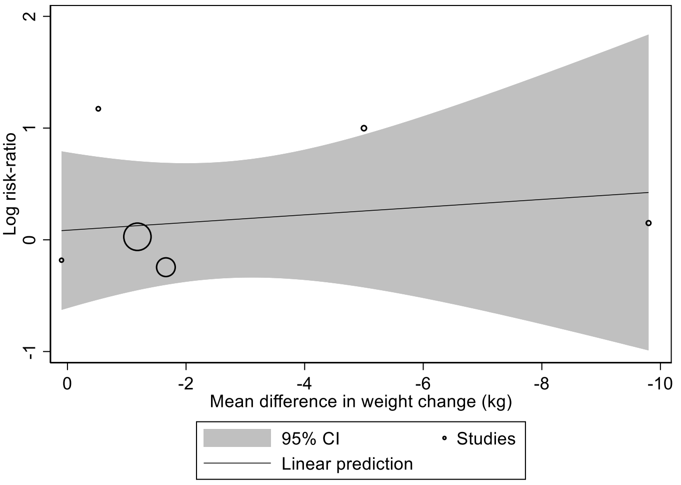

C1. Total pregnancies – no/minimal comparator

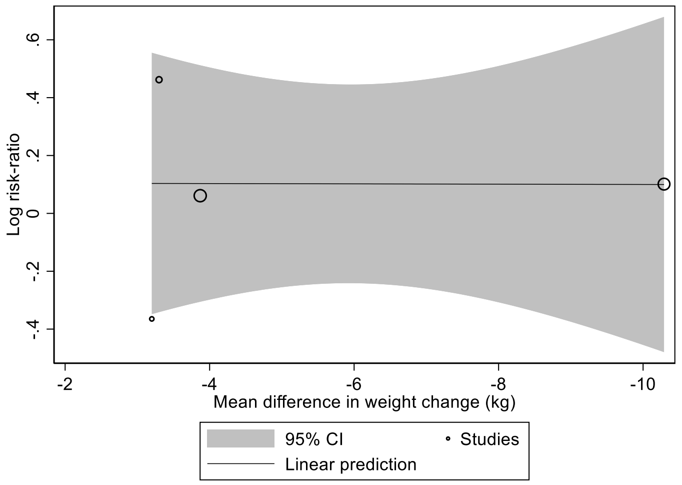

C2. Total pregnancies – active comparator

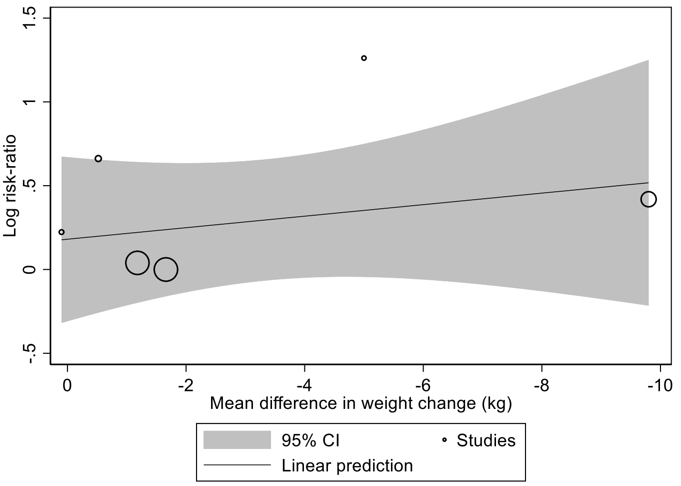

Figure S31 continued.

D1. Live birth – no/minimal comparator

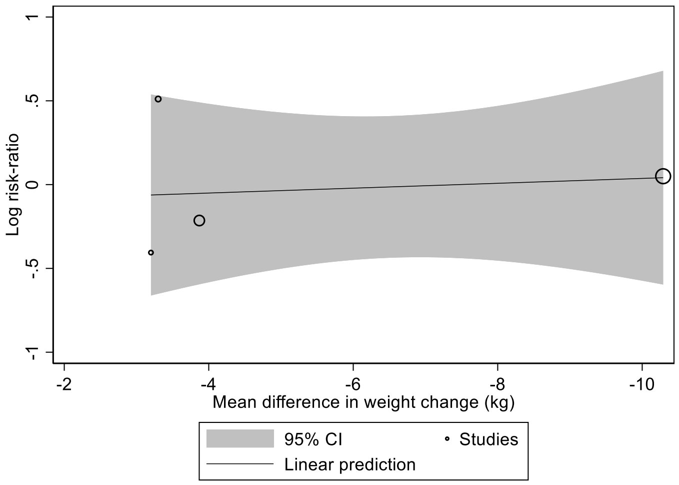

D2. Live birth – active comparator

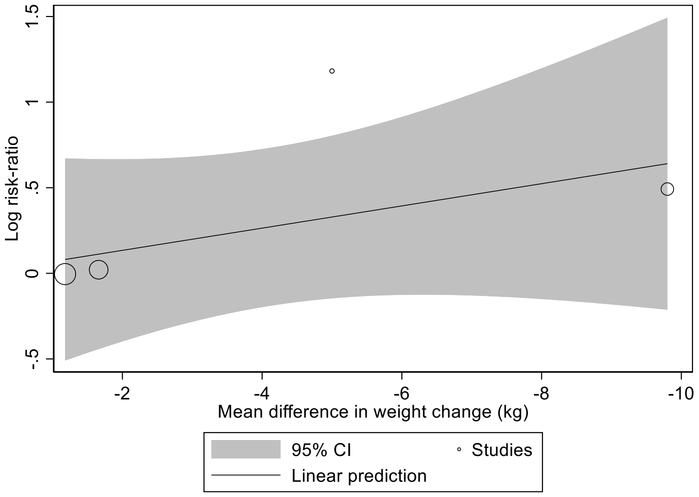

E1. Pregnancy loss – no/minimal comparator

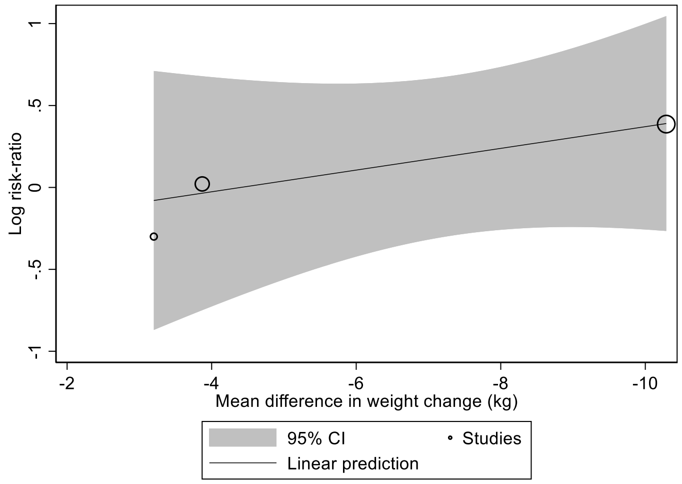

E2. Pregnancy loss – active comparator

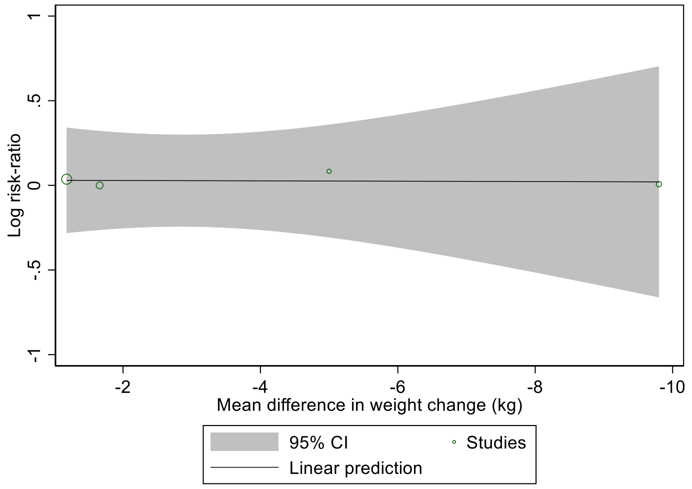

A. Random-effects meta-regression with the Sidik Jonkman method, adjusting for comparator type; 9 studies (26, 27, 30, 32-38), β= -0.01, P=0.866; CI: confidence interval; Weights from inverse variance; Einarsson 2017 reported only the unassisted pregnancies which led to a live birth (16 in intervention, 4 in comparator), not the total number of unassisted pregnancies, and was not included; Espinos 2017 reported 0 events in both groups and did not provide weight data at follow-up for the comparator, therefore it was not possible to calculate the mean difference in weight change between groups; Mutsaerts 2016 did not report unassisted pregnancies for women specifically seeking IVF; Li 2022 reported weight and event data from per protocol analysis only; Price 2020, 2021 reported weight data from per protocol analysis only.

B. Random-effects meta-regression with the Sidik Jonkman method, adjusting for comparator type; 8 studies (26, 30, 31, 33-38), β= -0.03, P=0.758; CI: confidence interval; Weights from inverse variance; Becker 2015 only reported unassisted pregnancy rates, therefore, total pregnancy, and by extent assisted pregnancy rates are not known; Espinos 2017 did not provide weight data at follow-up for the comparator, therefore it was not possible to calculate the mean difference in weight change between groups; Mutsaerts 2016 did not report unassisted pregnancies for women specifically seeking IVF, therefore assisted pregnancy rates are unknown; Li 2022

reported weight and event data from per protocol analysis only; Price 2020, 2021 reported weight data from per protocol analysis only.

C. Random-effects meta-regression with the Sidik Jonkman method, adjusting for comparator type; 10 studies (26, 29-37), β= -0.02, P=0.535; CI: confidence interval; Weights from inverse variance; Espinos 2017 did not provide weight data at follow-up for the comparator, therefore it was not possible to calculate the mean difference in weight change between groups; Becker 2015 only reported unassisted pregnancy rates, therefore, total pregnancy rates are not known; Li 2022 reported events and weight data from per protocol analysis only; Price 2020, 2021 reported weight data from per protocol analysis only; We were able to obtain weight and total pregnancy data for women specifically seeking IVF from authors of Mutsaerts 2016.

D. Random-effects meta-regression with the Sidik Jonkman method, adjusting for comparator type; 8 studies (26, 29, 30, 32-38), β= -0.05, P=0.304; CI: confidence interval; Weights from inverse variance; Espinos 2017 did not provide weight data at follow-up for the comparator, therefore it was not possible to calculate the mean difference in weight change between groups; Li 2022 reported weight and event data from per protocol analysis only; Price 2020, 2021 reported weight data from per protocol analysis only; We were able to obtain weight and live birth data for women specifically seeking IVF from authors of Mutsaerts 2016; Unassisted pregnancies have been taken into account in live birth rates reported by Einarsson 2017, Sim 2014, Wang 2023, and Wang 2021; For Moran 2011 and Li 2022, it is suggestive that unassisted pregnancies were not taken into account in the reported live birth rates.

E. Random-effects meta-regression with the Sidik Jonkman method, adjusting for comparator type; 7 studies (29, 30, 32-38), β= -0.02, P=0.659; CI: confidence interval; Weights from inverse variance; Espinos 2017 did not provide weight data at follow-up for the comparator, therefore it was not possible to calculate the mean difference in weight change between groups; Li 2022 reported weight and event data from per protocol analysis only; Price 2020, 2021 reported weight data from per protocol analysis only; We were able to obtain weight and pregnancy loss data for women specifically seeking IVF from authors of Mutsaerts 2016.

Figure S32. Funnel plots of included randomized controlled trials for reproductive outcomes

A. Unassisted pregnancies

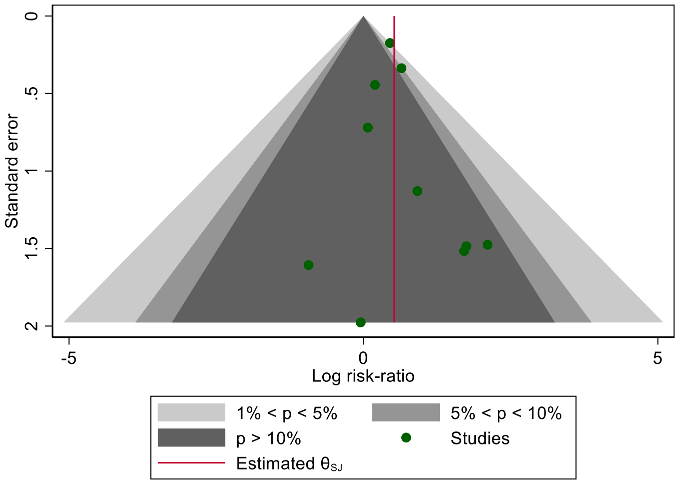

B. Treatment-induced pregnancies

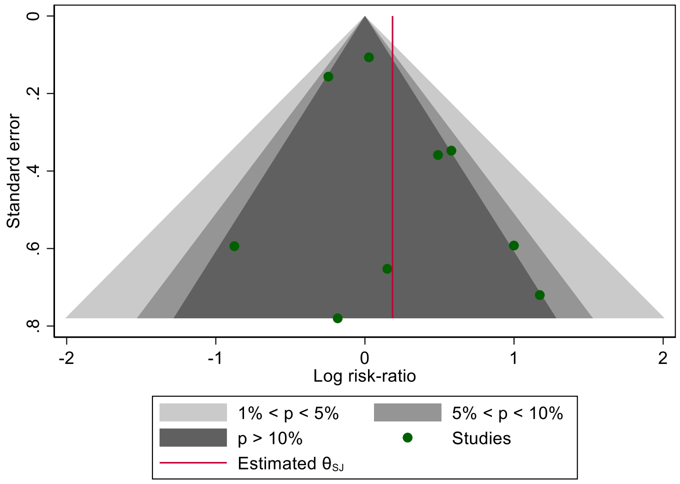

C. Total pregnancies

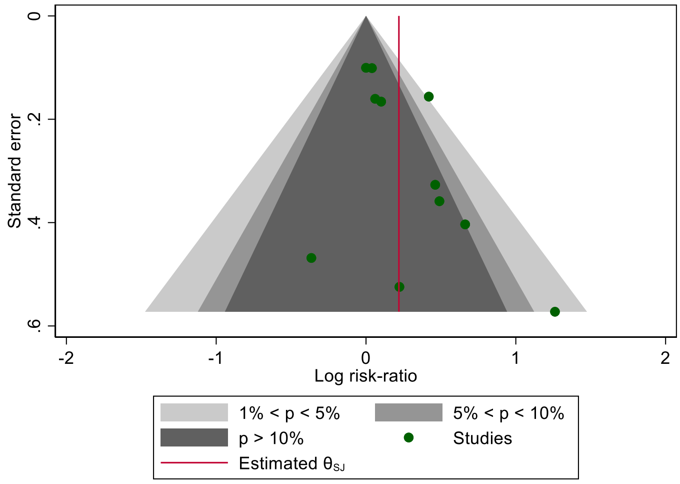

D. Live birth

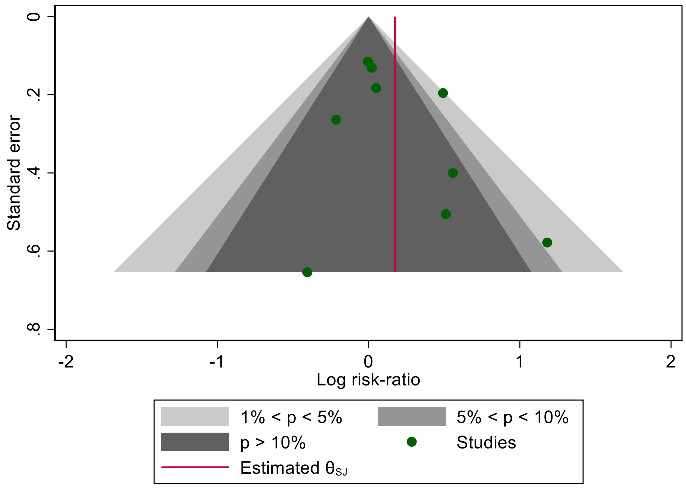

E. Pregnancy loss

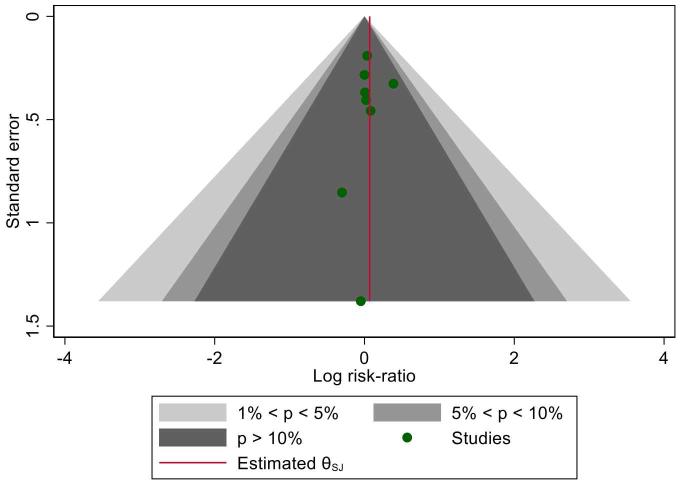

CI: confidence interval; P value from Egger's test: 0.582 (A), 0.535 (B), 0.480 (C), 0.341 (D), 0.859 (E)
